# The dynamic clustering of insulin receptor underlies its signaling and is disrupted in insulin resistance

**DOI:** 10.1038/s41467-022-35176-7

**Published:** 2022-12-06

**Authors:** Alessandra Dall’Agnese, Jesse M. Platt, Ming M. Zheng, Max Friesen, Giuseppe Dall’Agnese, Alyssa M. Blaise, Jessica B. Spinelli, Jonathan E. Henninger, Erin N. Tevonian, Nancy M. Hannett, Charalampos Lazaris, Hannah K. Drescher, Lea M. Bartsch, Henry R. Kilgore, Rudolf Jaenisch, Linda G. Griffith, Ibrahim I. Cisse, Jacob F. Jeppesen, Tong I. Lee, Richard A. Young

**Affiliations:** 1 Whitehead Institute for Biomedical Research, Cambridge, MA 02142 USA; 2 Division of Gastroenterology, Department of Medicine, Massachusetts General Hospital, Boston, MA 02114 USA; 3 Department of Physics, Massachusetts Institute of Technology, Cambridge, MA 02139 USA; 4 Department of Medicine, University of Udine, Udine, 33100 Italy; 5 Department of Biological Engineering, Massachusetts Institute of Technology, Cambridge, MA 02139 USA; 6 Department of Biology, Massachusetts Institute of Technology, Cambridge, MA 02139 USA; 7 Department of Mechanical Engineering, Massachusetts Institute of Technology, Cambridge, MA USA; 8 Center for Gynepathology Research, Massachusetts Institute of Technology, Cambridge, MA USA; 9 Global Drug Discovery, Novo Nordisk, Copenhagen, Denmark

**Keywords:** Insulin signalling, Supramolecular assembly, Type 2 diabetes

## Abstract

Insulin receptor (IR) signaling is central to normal metabolic control and is dysregulated in metabolic diseases such as type 2 diabetes. We report here that IR is incorporated into dynamic clusters at the plasma membrane, in the cytoplasm and in the nucleus of human hepatocytes and adipocytes. Insulin stimulation promotes further incorporation of IR into these dynamic clusters in insulin-sensitive cells but not in insulin-resistant cells, where both IR accumulation and dynamic behavior are reduced. Treatment of insulin-resistant cells with metformin, a first-line drug used to treat type 2 diabetes, can rescue IR accumulation and the dynamic behavior of these clusters. This rescue is associated with metformin’s role in reducing reactive oxygen species that interfere with normal dynamics. These results indicate that changes in the physico-mechanical features of IR clusters contribute to insulin resistance and have implications for improved therapeutic approaches.

## Abstract (web summary)

The authors find that the insulin receptor forms dynamic clusters during insulin signaling and that these clusters become dysfunctional in insulin resistance. This dysfunction is partially rescued by metformin, a drug used to treat type 2 diabetes.

## Introduction

Insulin signaling controls cell growth and metabolism, and dysregulation of this pathway is a common feature of type 2 diabetes (T2D), obesity, and metabolic syndrome^1,2^. Insulin binds at the cell surface to the insulin receptor (IR), a receptor tyrosine kinase (RTK)^3,4^. Insulin binding induces IR autophosphorylation and IR phosphorylation of IR substrate (IRS) and src homology 2 (SHC) proteins, which activate PI3K-AKT and ERK signaling, respectively^1–8^. These pathways regulate glucose uptake, lipogenesis, gluconeogenesis, glycogen synthesis, and cellular proliferation^1,2,9^. The active IR is internalized by endocytosis and is either degraded in lysosomes, recycled back to the plasma membrane, or transported into the nucleus where it becomes associated with insulin-responsive genes^10–16^.

Insulin resistance is a heterogeneous disorder common to type 2 diabetes (T2D), obesity, and metabolic syndrome^17,18^. Multiple cell-extrinsic and cell-intrinsic factors can blunt the cellular response to insulin and thus contribute to insulin resistance^1,2,19^. These include alterations in insulin signaling components as a consequence of chronic hyperinsulinemia, nutritional excess, inflammation, oxidative stress, ER stress, fatty acid accumulation, and mitochondrial dysfunction^1,2,17,18,20–23^.

Recent reports indicate that signaling factors can form dynamic clusters with properties and characteristics expected of biomolecular condensates^24–33^. Biomolecular condensates are cellular compartments wherein proteins and nucleic acids concentrate without being physically delimitated by a membrane^34^. Condensate formation and condensate properties have been shown to contribute to diverse types of cellular signaling^24–33^. For example, evidence suggests that T-cell receptor activation causes the formation of condensate compartments at the plasma membrane that incorporate signaling components and promote signaling^24,25^, and similar observations were recently reported for various RTKs^30^. The terminal components of the Wnt, Lif, and TGFb developmental signaling pathways are directed to key developmental genes through integration into transcriptional condensates at those genes^26^. In addition, the dynamic properties of condensates have been shown to correlate with the activity and function of the molecules within the condensates^28,31,35–38^. This previous evidence for dynamic clusters of signaling factors, coupled with the observation that insulin receptor can be seen as puncta when visualized in live cells, led us to investigate whether insulin signaling involves dynamic clustering and whether dysregulation of such clustering contributes to insulin resistance.

Here, we report that IR is incorporated into dynamic clusters at the plasma membrane, in the cytoplasm, and in the nucleus of human hepatocytes and adipocytes. Insulin stimulation promotes further incorporation of IR into these clusters in insulin-sensitive cells but not in insulin-resistant cells, where IR molecules within clusters exhibit less dynamic behavior. Metformin treatment of insulin-resistant cells rescues IR cluster dynamics and insulin responsiveness. Insulin-resistant cells are subjected to high levels of oxidative stress, which we find to cause reduced cluster dynamics, and treatment of these cells with metformin reduces levels of ROS and returns IR clusters to their normal dynamic behavior.

## Results

### Insulin receptor bodies in human liver cells

**Fig. 1:**
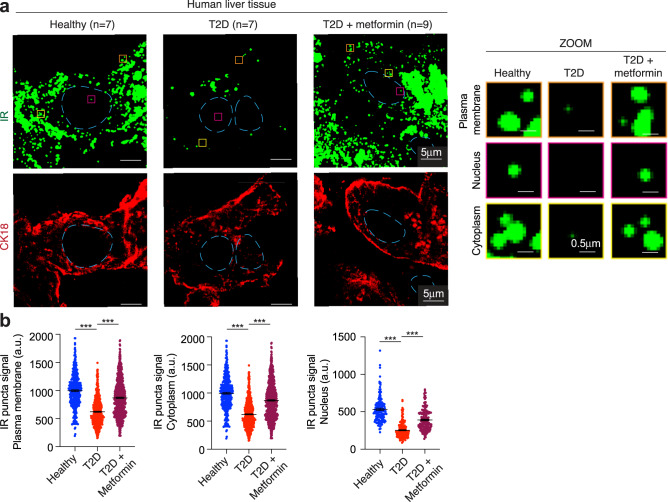
Insulin receptor bodies in human liver cells. **a** Representative immunofluorescence images for IR and CK18 in liver tissue from a healthy donor (Healthy), a donor with T2D (T2D), and a donor with T2D who had been treated with metformin (T2D + metformin). Dashed light blue lines represent the nuclear outline. Orange, magenta, and yellow boxes represent regions at the plasma membrane, nucleus, and cytoplasm, respectively, that are magnified on the right (ZOOM). Scale bars are indicated in the images. **b** Quantification of IR signal in puncta at the plasma membrane, cytoplasm, and nucleus for seven healthy donors (blue), seven donors with T2D (red), and nine donors with T2D who had been treated with metformin (purple). Data are represented as mean + /− standard error of the mean (SEM). The number of puncta analyzed: healthy plasma membrane 399 puncta, cytoplasm 304 puncta, nucleus 137 puncta; T2D plasma membrane 618 puncta, cytoplasm 283 puncta, nucleus 187 puncta; T2D metformin plasma membrane 716 puncta, cytoplasm 350 puncta, nucleus 173 puncta. Unpaired two-sided *t* test was used for statistical analysis.

Clusters of proteins can be visualized as punctate bodies in cells, and IR has previously been observed in punctate bodies in diverse cultured cells^16,39,40^. We investigated whether IR puncta occur in healthy human liver tissue and whether such puncta differ in T2D patients treated with and without metformin, the front-line drug for T2D. We examined 23 human liver tissue samples, comprising seven from healthy donors, seven from donors with T2D, and nine from donors with T2D under treatment with metformin (Fig. 1 and Supplementary Table 1). These liver tissues exhibited histologic and metabolic features, as well as redox states, expected for healthy donors, donors with T2D and donors with T2D under metformin treatment^18,41–45^ (Supplementary Fig. 1a–c and Supplementary Table 1). Immunofluorescence for CK18 was used to assess tissue quality, cell morphology and as a marker for hepatocytes (Fig. 1a). Imaging of these tissues with a validated antibody for IR (Supplementary Fig. 1d, e) revealed that IR occurs in punctate bodies in hepatocytes from healthy donors, but these signals were significantly reduced in tissues from T2D donors that were not treated with metformin (Fig. 1a, b and Supplementary Fig. 1f, g). It was notable that hepatocytes from metformin-treated donors with T2D had IR punctate signals similar to those observed in healthy tissues. These differences were evident in puncta formed in the plasma membrane, the cytoplasm, and the nucleus (Fig. 1 and Supplementary Fig. 1g). The total levels of IR protein spanned a similar range in donor tissues from healthy and T2D donors (Supplementary Fig. 1h), suggesting that the reduced punctate signal in the tissue of T2D donors lacking metformin treatment is not simply due to a difference in the overall level of IR protein. These results suggest that the incorporation of IR into puncta in human hepatocytes is attenuated in T2D and is rescued to some extent by metformin treatment.

### Insulin receptor bodies in HepG2 cells

**Fig. 2:**
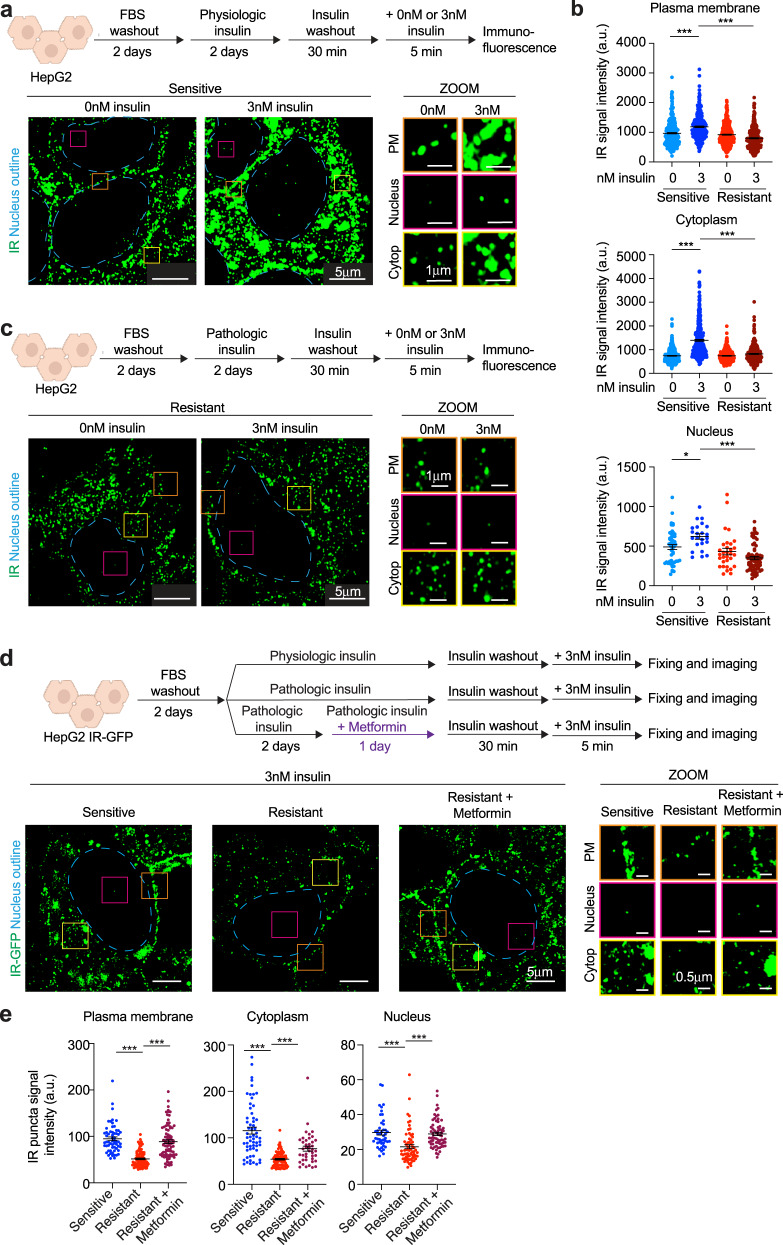
Insulin receptor bodies in HepG2 cells. **a** Schematic of cell treatments (top). Representative immunofluorescence images of IR (green) in cells stimulated with (3 nM) or without (0 nM) insulin for 5 min (bottom). Dashed light blue lines represent the outline of the nucleus. Orange, magenta, and yellow boxes represent regions at the plasma membrane (PM), nucleus, and cytoplasm (Cytop), respectively, that are magnified (ZOOM, middle). Scale bars are indicated. **b** Quantification of IR signal intensity in puncta at the plasma membrane (top), cytoplasm (middle), and nucleus (bottom) in insulin-sensitive cells stimulated (3 nM, blue) or not (0 nM, light blue) with insulin and in insulin-resistant cells stimulated (3 nM, dark red) or not (0 nM, red) with insulin. Data are represented as mean + /− SEM. The number of puncta analyzed: sensitive 0 nM insulin plasma membrane 342 puncta, cytoplasm 366 puncta, nucleus 48 puncta; sensitive 3 nM insulin plasma membrane 272 puncta, cytoplasm 378 puncta, nucleus 23 puncta; resistant 0 nM insulin plasma membrane 406 puncta, cytoplasm 295 puncta, nucleus 31 puncta; resistant 3 nM insulin plasma membrane 328 puncta, cytoplasm 283 puncta, nucleus 69 puncta. Unpaired two-sided *t* test was used for statistical analysis. **c** Schematic of cell treatments to model insulin resistance (top). Representative immunofluorescence images for IR (green) in insulin-resistant cells acutely stimulated with (3 nM) or without (0 nM) insulin (bottom). Orange, magenta, and yellow boxes represent regions at the plasma membrane (PM), nucleus, and cytoplasm (Cytop), respectively, that are magnified (ZOOM, middle). Scale bars are indicated. **d** Schematic of cell treatments (top). Cells expressing endogenous IR tagged with GFP (IR-GFP) were used. Metformin concentration used is 12.5 μM. Representative images for IR-GFP in insulin-sensitive, insulin-resistant, and metformin-treated insulin-resistant cells stimulated with insulin (3 nM) for 5 min (bottom). Orange, magenta, and yellow boxes represent regions at the plasma membrane (PM), cytoplasm (Cytop) and nucleus, respectively, that are magnified (ZOOM). Scale bars are indicated. **e** Quantification of IR-GFP signal intensity in IR puncta at the plasma membrane, cytoplasm, and nucleus of insulin-sensitive (blue), insulin-resistant (red) and metformin-treated insulin-resistant (purple) cells acutely stimulated with (3 nM) insulin. Data are represented as mean + /− SEM. The number of puncta analyzed: sensitive 3 nM insulin plasma membrane 60 puncta, cytoplasm 59 puncta, nucleus 45 puncta; resistant 3 nM insulin plasma membrane 90 puncta, cytoplasm 90 puncta, nucleus 65 puncta; resistant metformin 3 nM insulin plasma membrane 82 puncta, cytoplasm 41 puncta, nucleus 67 puncta. Unpaired two-sided *t* test was used for statistical analysis. Source data are provided as a Source Data file.

To further investigate the features of IR puncta in hepatocytes, we turned to HepG2 cells because of their demonstrated utility in the study of insulin signaling and resistance, and because they are amenable to genetic modification^11,46,47^. Cells were cultured in media containing physiologic concentrations of glucose (5 mM) and insulin (0.1 nM)^48–50^ (Fig. 2a). Cell culture conditions were selected to mimic those experienced by hepatocytes in situ and cell viability and the ability of cells to clear insulin remained high under these conditions (Supplementary Fig. 2a, b). To confirm that these cells were insulin-sensitive, conventional assays of insulin sensitivity were performed. Acute insulin stimulation induced IR phosphorylation (Supplementary Fig. 2c), AKT and ERK pathway activation (Supplementary Fig. 2c), upregulation of lipogenic genes and downregulation of gluconeogenesis genes (Supplementary Fig. 2d, e), increased lipogenesis (Supplementary Fig. 2f), decreased glucose production (Supplementary Fig. 2g, h), and increased GSK3 phosphorylation (Supplementary Fig. 2i). Thus, the HepG2 cells cultured in this fashion exhibit the conventional features associated with insulin sensitivity.

IR localization was monitored in HepG2 cells by immunofluorescence super-resolution microscopy and was found to be incorporated into puncta at the plasma membrane, cytoplasm and nucleus of HepG2 cells in the absence of insulin stimulation, and this signal was elevated with insulin stimulation (Fig. 2a, b and Supplementary Fig. 3). Western blot analysis indicated HepG2 cells contain ~300,000 IR molecules/cell (Supplementary Fig. 4a), consistent with estimates for human hepatocytes^51^, and showed that the unstimulated and insulin-stimulated cells contain similar levels of IR (Supplementary Fig. 4b). Given the similar levels of IR in unstimulated and insulin-stimulated cells, we infer from the imaging results that the increased IR signal in puncta reflects increased incorporation of IR molecules into these bodies from the surrounding intracellular environment, and not changes in the overall level of the protein.

Active IR has been reported to localize at plasma membrane microdomains or signalosomes with other signaling proteins, enter the cytoplasm via endocytosis and become associated with lysosomes or be recycled to the plasma membrane, and enter the nucleus and bind to insulin-responsive genes^10–16,39,40^. Our observations with IR bodies in the plasma membrane, cytoplasm and nucleus of HepG2 cells are consistent with these prior reports (Fig. 2a and Supplementary Fig. 5). Electron microscopy with IR-specific antibodies confirmed that IR can be found near the plasma membrane, in the cytoplasm (some associated with membranes and some not) and in the nucleus (Supplementary Fig. 5a). Super-resolution microscopy confirmed that IR puncta can colocalize with a portion of the insulin signaling proteins AKT and PI3K (Supplementary Fig. 5b), that IR puncta can colocalize with clathrin vesicles and lysosomes (Supplementary Fig. 5c), and that IR puncta can be found at the periphery of endosome vesicles (Supplementary Fig. 5d). These results suggest that IR puncta are not simply concentrations of IR constrained as a consequence of being fully enveloped by membranes, but instead can sometimes be partially associated with membranes, consistent with previously published results for IR^52^ and other protein assemblies associated with plasma membranes and endosomes such as those formed by other signaling factors and neuronal post-synaptic densities^27,30,53–56^. In the nucleus, IR puncta were found colocalized with markers of the transcriptional machinery (MED1 and RNA Polymerase II) at the insulin-responsive genes *FASN*, *SREBF1*, and *TIMM22* (Supplementary Fig. 5e), and this was confirmed by ChIP-seq analysis of these proteins at these genes (Supplementary Fig. 5f).

To investigate whether IR puncta are altered in insulin resistance, we compared the insulin-sensitive HepG2 cells to cells in which an insulin-resistant state was induced by hyperinsulinemia. HepG2 cells were exposed to either physiologic levels (0.1 nM) or pathologic levels (3 nM) of insulin^48–50,57^ for 2 days (Supplementary Fig. 6a). Cells exposed to pathologic levels of insulin showed hallmarks of insulin resistance that are observed after insulin stimulation: reduced phosphorylation of IR, IRS1, AKT, and ERK (Supplementary Fig. 6b–e), unchanged expression of the lipogenic gene *FASN* (Supplementary Fig. 6f), impaired suppression of glucose production and impaired promotion of lipogenesis (Supplementary Fig. 6g–i), and decreased phosphorylation of GSK3 (Supplementary Fig. 6j). These results are consistent with recent evidence that, in the liver of insulin-resistant patients, hepatocellular insulin signaling is blocked at the level of phosphorylation of insulin receptor and there is impaired insulin-mediated suppression of glucose production and impaired insulin-mediated promotion of lipogenesis^20^. Insulin-sensitive and resistant cells contained similar amounts of IR in whole cell extracts and at the cell surface (Supplementary Fig. 6k–m). Insulin binding was also similar between insulin-sensitive and resistant cells (Supplementary Fig. 6n). These results suggest that the attenuated IR signaling was not due to a substantial change in IR levels in cells and at the plasma membrane, nor due to changes in the ability of insulin receptor to bind insulin.

Immunofluorescence imaging of IR in insulin-resistant cells revealed that it is incorporated into puncta at the plasma membrane, cytoplasm, and nucleus in a manner similar to that observed for insulin-sensitive cells (compare Fig. 2c and a, 0 nM insulin). However, in these insulin-resistant cells, acute treatment with insulin (3 nM) did not promote incorporation of additional IR into puncta (Fig. 2b, c), in contrast to the effects observed in insulin-sensitive cells (Fig. 2a, b). If the observed IR puncta defects are common features of insulin-resistant cells, then cells treated with other conditions expected to induce insulin resistance, such as chronic inflammation and high-nutrient levels^17,18^, should exhibit IR puncta defects that phenocopy those caused by hyperinsulinemia. Treatment of cells with pathologic concentrations of TNFα or with high nutrients also caused a decrease in insulin-stimulated IR incorporation into puncta similar to that observed for hyperinsulinemia (Supplementary Fig. 7). These results suggest that IR puncta dysfunction, defined here with respect to accumulation of molecules in puncta, may be a common feature of insulin resistance induced by diverse factors.

To confirm these observations and enable imaging of IR in live cells, HepG2 cells were engineered to express endogenous IR as a fusion protein with a monomeric-enhanced green fluorescent protein (IR-GFP) (Supplementary Fig. 8a). IR-GFP was expressed in these homozygous cells at the same levels as WT IR and was functional, as cells expressing this fusion protein maintained insulin-induced phosphorylation of IR and insulin signaling proteins (Supplementary Fig. 8b, c). A time course of insulin stimulation using live-cell imaging provided further evidence that insulin stimulation promotes an increase in IR-GFP signal in IR puncta (Supplementary Fig. 9a, b), as well as an increase in the number of IR puncta in the nucleus and cytoplasm (Supplementary Fig. 9c).

Given our observation that hepatocytes in liver tissue from metformin-treated T2D patients have IR puncta that resemble those in healthy donors, we investigated whether metformin could rescue the reduction in IR punctate signal seen in insulin-resistant HepG2 cells. We again observed that IR-GFP HepG2 cells rendered insulin-resistant showed reduced insulin-promoted incorporation of IR into puncta (Fig. 2d, e) and found that treatment of these insulin-resistant cells with metformin partially restored IR signal in these puncta (Fig. 2d, e and Supplementary Fig. 10a). Treatment of insulin-sensitive HepG2 cells with metformin had little or no effect on IR puncta (Supplementary Fig. 10b). The rescue of IR puncta phenotype in insulin-resistant cells was not due to changes in IR levels (Supplementary Fig. 10c). IR puncta rescue was evident at 12.5 μM metformin (Supplementary Fig. 10a), which approximates the concentration of metformin in the plasma of T2D patients^58–60^. These results indicate that the insulin-resistant state in these cells is associated with reduced IR incorporation in puncta, and that this dysfunction can be reversed to some extent by metformin, as observed in human liver tissue (compare Fig. 1a with Fig. 2d).

### Insulin receptor bodies in primary hepatocytes and adipocytes

We next investigated whether similar IR puncta occur in human primary hepatocytes, whether these are altered in insulin resistance, and studied the effects of metformin on such puncta. Primary human hepatocytes can form three-dimensional spheroids and can be cultured for days with physiologic or pathologic concentrations of insulin while maintaining their cell identity and function (Supplementary Fig. 11a, b). These hepatocyte spheroids are insulin-sensitive if cultured with physiologic concentrations of insulin and insulin-resistant if subjected to insulin levels characteristic of chronic hyperinsulinemia (Supplementary Fig. 11c, d). In insulin-sensitive human liver spheroids, IR was found in puncta at the plasma membrane, cytoplasm, and nucleus (Supplementary Fig. 11e). As observed with HepG2 cells, insulin stimulation of hepatocyte spheroids produced an increase in IR signal intensity in cytoplasmic and nuclear puncta (Supplementary Fig. 11e). In insulin-resistant spheroids, by contrast, IR incorporation into cytoplasmic and nuclear puncta was diminished (Supplementary Fig. 11e), and metformin treatment partially rescued this attenuation of IR puncta signal (Supplementary Fig. 11e). These results show that the phenotypes observed for IR puncta in insulin-sensitive and insulin-resistant HepG2 cells also occur in primary human hepatocyte spheroids.

Adipocytes are among the cell types that exhibit insulin-resistant behavior, so we also investigated whether primary human adipocytes exhibit IR puncta phenotypes similar to those observed in hepatocytes (Supplementary Fig. 12). Primary human pre-adipocytes were first differentiated into adipocytes (Supplementary Fig. 12a) and then cultured for five days with either physiologic concentrations of insulin or pathologic concentrations of insulin known to induce insulin resistance in adipocytes^61^ (Supplementary Fig. 12b). As observed with insulin-sensitive hepatocytes, IR-associated puncta were found at the plasma membrane, in the cytoplasm, and in nuclei of insulin-sensitive adipocytes, and insulin stimulation promoted further IR incorporation into these puncta (Supplementary Fig. 12c). In the insulin-resistant adipocytes, insulin stimulation was less able to promote further IR incorporation into puncta and this reduction in signal was reversed by metformin (Supplementary Fig. 12c). These results show that primary human adipocytes exhibit IR puncta phenotypes similar to those observed in hepatocytes.

### Characterization of insulin receptor bodies

**Fig. 3:**
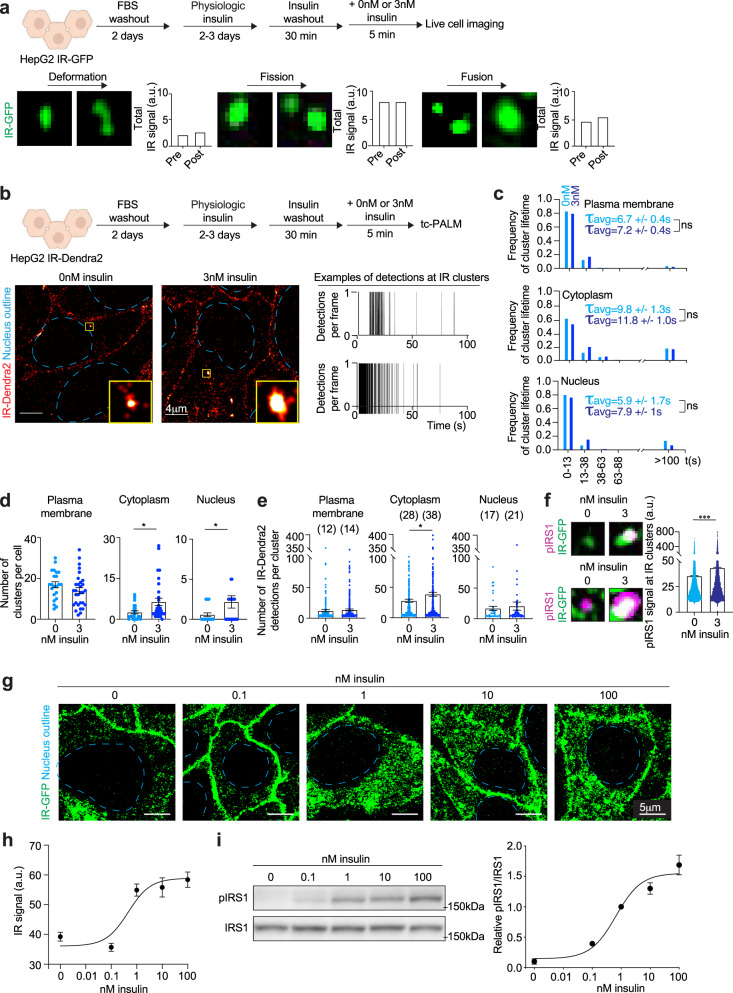
Characterization of insulin receptor clusters. **a** Schematic of cell treatments (top). Representative images of IR puncta undergoing deformation (bottom left), fission (bottom center), and fusion (bottom right). Quantification of total IR signal intensity over puncta pre- and post-deformation, fission or fusion. Images were taken 0.2 s or 0.5 s apart. **b** Schematic of cell treatments (top). Representative tc-PALM images of cells expressing IR-Dendra2 stimulated acutely with (3 nM) or without (0 nM) insulin for 5 min (bottom left). Scale bars are indicated. Representative tc-PALM traces (bottom right). **c** Frequency of IR cluster lifetime in cells not acutely stimulated with insulin (0 nM insulin, light blue) and acutely stimulated with insulin for 5 min (3 nM insulin, dark blue). The average lifetime (τ_avg_) of short-lived IR clusters + /− SEM is reported. The number of IR clusters analyzed: sensitive 0 nM insulin plasma membrane 385 puncta, cytoplasm 136 puncta, nucleus 15 puncta; sensitive 3 nM insulin plasma membrane 430 puncta, cytoplasm 231 puncta, nucleus 72 puncta. Unpaired two-sided *t* test was used for statistical analysis. **d** Number of IR clusters per cell in insulin-sensitive cells not acutely stimulated with insulin (light blue) and acutely stimulated with insulin (dark blue). Data are represented as mean + /− SEM. The number of cells analyzed: sensitive 0 nM insulin plasma membrane 22 cells, cytoplasm 22 cells, nucleus 22 cells; sensitive 3 nM insulin plasma membrane 30 cells, cytoplasm 30 cells, nucleus 30 cells. Unpaired two-sided t test was used for statistical analysis for the cytoplasm, and unpaired one-sided *t* test was used for the nucleus. **e** Number of IR-Dendra2 detections per IR cluster in insulin-sensitive cells not acutely stimulated with insulin (light blue) and acutely stimulated with insulin (dark blue). Average number of IR detections per IR cluster is reported on top of each histogram. Data are represented as mean + /− SEM. The number of clusters analyzed: sensitive 0 nM insulin plasma membrane 430 clusters, cytoplasm 499 clusters, nucleus 37 clusters; sensitive 3 nM insulin plasma membrane 573 clusters, cytoplasm 551 clusters, nucleus 57 clusters. Unpaired two-sided *t* test was used for statistical analysis. **f** Representative images of IR-GFP and phosphorylated IRS1 (pIRS1) in insulin-sensitive HepG2 cells stimulated acutely with (3 nM) or without (0 nM) insulin for 5 min (left). Quantification of pIRS1 signal in IR clusters in insulin-sensitive HepG2 cells stimulated acutely with (blue) or without (dark blue) insulin for 5 min (right). Data are represented as mean + /− SEM. The number of IR clusters analyzed: sensitive 0 nM insulin 7640 clusters; sensitive 3 nM insulin 10,979 clusters. Unpaired two-sided *t* test was used for statistical analysis. **g**, Representative images of insulin-sensitive cells stimulated with the reported concentrations of insulin for 5 min. **h**, Quantification of IR signal in clusters in the cytoplasm. Data are represented as mean + /− SEM. The number of IR clusters analyzed: 0 nM insulin 141 clusters, 0.1 nM insulin 164 clusters, 1 nM insulin 163 clusters, 10 nM insulin 101 clusters, 100 nM insulin 100 clusters. **i** Immunoblot and quantification of pIRS1 relative to total IRS1 in insulin-sensitive cells stimulated with the reported concentrations of insulin for 5 min. Data are represented as mean + /− SEM. The number of biologically independent samples analyzed: nine per condition. Source data are provided as a Source Data file.

The appearance of IR in punctate bodies suggests IR may be forming dynamic clusters similar to those seen with other signaling pathways, where such clusters can exhibit physical changes such as deformation, fission, and fusion^62–67^. Super-resolution microscopy of HepG2 IR-GFP cells revealed IR-GFP puncta in the plasma membrane, cytoplasm and nucleus do indeed undergo deformation, fission, and fusion (Fig. 3a). To investigate whether these clusters undergo dynamic formation and dissolution, we used time-correlated photoactivation localization microscopy (tc-PALM)^35,62,68^ with a HepG2 cell line engineered to express endogenous IR as a fusion protein with Dendra2 (IR-Dendra2) (Supplementary Fig. 8a). IR-Dendra2 was expressed at the same levels as WT IR and was functional, as this fusion protein maintained its kinase activity (Supplementary Fig. 8b, c). IR-Dendra2 cells were subjected to tc-PALM, and clusters of IR molecules were studied (Fig. 3b); several control analyses of the single-molecule photochemistry were performed to validate the statistics of the molecular clusters examined here (Supplementary Fig. 13). The results revealed that IR forms dynamic clusters at the plasma membrane, cytoplasm, and nucleus that exhibit various lifetimes, consistent with formation and dissolution times seen for other dynamic biomolecular assemblies (Fig. 2b, c). In cells with and without insulin stimulation, the majority of IR clusters (~85%) were short-lived (lifetime < 100 s) and had an average lifetime of 6–12 s (Fig. 3c), comparable to those measured for other dynamic biomolecular assemblies in various cell types^35,62,68^. A smaller fraction of clusters (~15%) were present for considerably longer lifetimes (>100 s) (Fig. 3c). Insulin stimulation resulted in an increase in the number of IR clusters in the cytoplasm and nucleus of these cells (Fig. 3d) and an increase in the number of IR detections in clusters in insulin-sensitive cells (Supplementary Fig. 14). The average number of IR-Dendra2 detections per cluster was estimated to be 22 (range 4–609) in unstimulated cells, and 27 (range 4–539) in insulin-stimulated cells (Supplementary Fig. 14). A similar trend was observed in IR clusters at the plasma membrane, in the cytoplasm and in the nucleus (Fig. 3e). These results suggest that multiple molecules of IR are incorporated into dynamic clusters, and that insulin stimulation leads to an increase in both the number of IR-containing clusters and the number of IR molecules per cluster in the cytoplasm and nucleus.

We next sought evidence that IR molecules present in clusters are functionally active. If IR kinase activity occurs in these clusters, then we would expect that the level of phosphorylated IR substrate IRS1 would increase in these IR-associated clusters upon insulin stimulation. Indeed, immunofluorescence microscopy with an antibody specific for phosphorylated IRS1 (pIRS1) showed that insulin stimulation increased the intensity of pIRS1 at IR clusters (Fig. 3f). IR incorporation into clusters positively correlated with signal intensity of pIRS1 in these clusters (Supplementary Fig. 15a) and pIRS1 was more concentrated inside IR clusters than outside (Supplementary Fig. 15b). In addition, acute stimulation of insulin-sensitive cells with a range of insulin concentrations produced a nonlinear transition in IR incorporation into clusters (Fig. 3g, h). The sharp increase in IR signal in clusters occurred coincident with insulin receptor activity and function measured by IRS1 phosphorylation (Fig. 3g–i), which is expected if the IR molecules incorporated into assemblies are functional.

### Altered insulin receptor dynamics in insulin-resistant cells and rescue by metformin

**Fig. 4:**
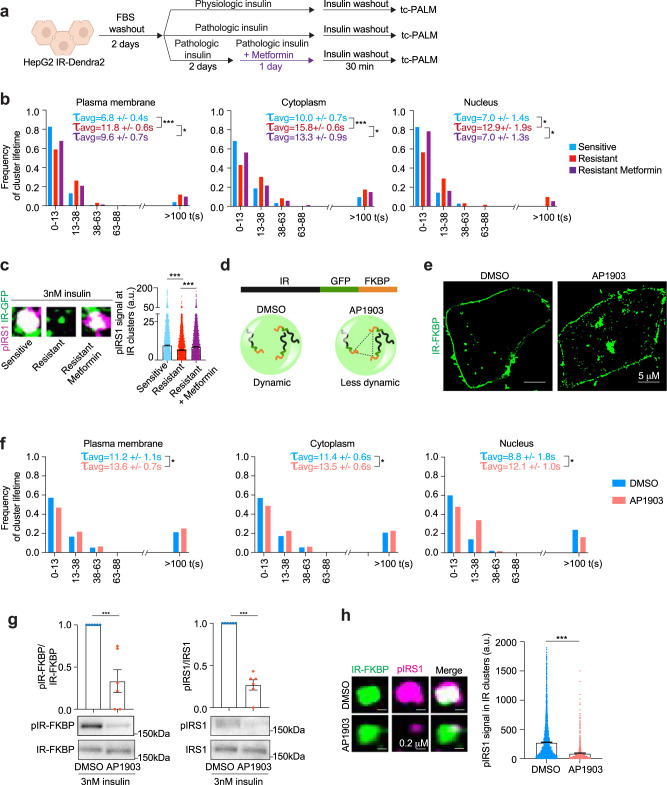
Altered insulin receptor dynamics in insulin-resistant cells and rescue by metformin. **a** Schematic of cell treatments. **b** Frequency of IR cluster lifetime in insulin-sensitive (light blue), insulin-resistant (red), and metformin-treated insulin-resistant (purple) cells. The concentration of metformin was 12.5 μM. Cells were imaged after insulin washout. The average lifetime (τ_avg_) of short-lived IR clusters + /− SEM is reported. The number of IR clusters analyzed: sensitive plasma membrane 294 clusters, cytoplasm 230 clusters, nucleus 35 clusters; resistant plasma membrane 491 clusters, cytoplasm 734 clusters, nucleus 62 clusters; resistant metformin plasma membrane 230 clusters, cytoplasm 309 clusters, nucleus 37 clusters. Unpaired two-sided *t* test was used for statistical analysis of the of short-lived clusters. **c** Example images of IR-GFP (green) and pIRS1 (magenta) in insulin-sensitive (sensitive, light blue), insulin-resistant (resistant, red), and metformin-treated insulin-resistant (resistant metformin, purple) cells stimulated acutely with 3 nM insulin for 5 min (left). Quantification of pIRS1 signal in IR clusters (right). Data are represented as mean + /− SEM. IR clusters analyzed: sensitive 4859 clusters, resistant 3557 clusters, and resistant metformin 8964 clusters. Unpaired two-sided *t* test was used for statistical analyses. **d** Schematic representation of IR-GFP-FKBP (IR-FKBP) construct and the effect of DMSO and AP1903 on IR-FKBP clusters. **e** Representative images of IR-FKBP in cells treated with DMSO or AP1903 for 16 h. **f** Frequency of IR-Dendra2-FKBP cluster lifetime in cells treated with DMSO (light blue) or AP1903 (red) for 16 h. Average lifetime (τ_avg_) of short-lived IR clusters + /− SEM is reported. The number of clusters analyzed: DMSO plasma membrane 194 clusters, cytoplasm 499 clusters, nucleus 50 clusters; AP1903 plasma membrane 544 clusters, cytoplasm 737 clusters, nucleus 129 clusters. Unpaired two-sided *t* test was used for statistical analysis for the cytoplasm, unpaired one-sided *t* test was used for statistical analysis for the plasma membrane and nucleus. **g** Immunoblot and quantification for phosphorylated IR-FKBP (pIR-FKBP) and pIRS1 over total protein. Cells expressing IR-FKBP were treated with DMSO (light blue) or AP1903 (red) for 16 h. Six biological replicates were analyzed for DMSO and AP1903-treated cells. Data are represented as mean + /− SEM. Unpaired two-sided *t* test was used for statistical analysis. **h** Representative images of IR-FKBP and pIRS1 in cells expressing IR-FKBP that were treated with DMSO (light blue) or AP1903 (red) for 16 h. Data are represented as mean + /− SEM. In total, 3181 IR-FKBP clusters were analyzed in the DMSO condition and 985 IR-FKBP clusters in the AP1903 condition. Unpaired two-sided *t* test was used for statistical analysis. Source data are provided as a Source Data file.

Chronic signaling was recently shown to reduce the dynamic properties of other dynamic clusters formed by signaling factors^36^, so we investigated whether the dynamics of IR clusters are altered in insulin-resistant cells (Fig. 4). HepG2 cells expressing IR-Dendra2 were exposed to physiologic levels of insulin (0.1 nM) to maintain insulin sensitivity, or pathologic levels of insulin (3 nM)^48–50,57^ to promote insulin resistance (Fig. 4a). tc-PALM was used to measure IR cluster dynamics in insulin-sensitive and resistant cells. The results showed that IR molecules remained in clusters for longer lifetimes in the cytoplasm and nucleus in insulin-resistant cells relative to insulin-sensitive cells (Fig. 4b). The average lifetime of short-lived IR clusters in sensitive versus insulin-resistant cells increased from 6.8 to 11.8 s at the plasma membrane, from 10.0 to 15.8 s in the cytoplasm and from 7.0 to 12.9 s in the nucleus. The percentage of long-lived IR clusters also increased in the plasma membrane, cytoplasm and nucleus (Fig. 4b). These results suggest that the insulin-resistant state is associated with reduced IR cluster dynamics, reflected in the longer lifetime of these clusters, which may account for the attenuated responses observed during insulin stimulation.

We wondered whether IR cluster dynamics are also decreased in other models of insulin resistance. Treatment of cells with pathologic concentrations of TNFα or with high nutrients decreased IR dynamics (Supplementary Fig. 16a, b). These results indicate that IR cluster dysfunction, defined here with respect to the accumulation and dynamics of molecules, may be a common feature of insulin resistance induced by diverse factors.

We next examined the effect of metformin treatment on IR cluster dynamics. Metformin treatment of insulin-resistant cells rescued IR cluster lifetimes in the plasma membrane, cytoplasm and nucleus to times that were similar to those in insulin-sensitive cells (Fig. 4b). For example, while ~40% of cytoplasmic IR clusters in insulin-resistant cells had a lifetime of 0 – 13 s, ~60% of IR clusters in the cytoplasm of insulin-sensitive and metformin-treated resistant cells had a lifetime of 0 – 13 s (Fig. 4b). Similarly, the frequency of plasma membrane and nuclear IR clusters with 0–13 s lifetimes, which was reduced in the resistant cells relative to sensitive cells, was increased by the metformin treatment (Fig. 4b). In contrast, metformin did not decrease IR cluster lifetime in insulin-sensitive cells (Supplementary Fig. 17). Thus, metformin treatment rescues the dynamic properties of IR-containing clusters that occur in insulin-resistant cells.

We next investigated whether IR kinase activity differs in clusters in insulin-resistant cells and in these cells treated with metformin. Imaging experiments revealed reduced levels of phosphorylated IRS1 in IR-containing clusters in insulin-resistant cells as compared to insulin-sensitive cells (Fig. 4c). Metformin treatment partially rescued the levels of phosphorylated IRS1 in IR-containing clusters in insulin-resistant cells (Fig. 4c). These results were further supported by western blot experiments that revealed a partial rescue of IRS1 phosphorylation in insulin-resistant cells by metformin treatment (Supplementary Fig. 18). Taken together, these results suggest that IR kinase activity is reduced in IR clusters in insulin-resistant cells and that metformin treatment can partially reverse this effect.

We next explored whether changes in the dynamics of IR-containing clusters might have a direct effect on IR kinase activity. To decrease IR molecule dynamics within clusters, we fused IR-GFP to four tandem repeats of FK506 binding protein (FKBP; IR-FKBP), which interact with each other only in the presence of the small molecule AP1903^69^. IR-FKBP was expressed in HepG2 cells and these cells were treated with AP1903 or control DMSO (Fig. 4d–h). Treating HepG2 cells expressing IR-FKBP with AP1903 significantly increased the lifetime of IR clusters, consistent with a reduction in IR molecule dynamics in these clusters (Fig. 4f). Western blot and imaging experiments revealed that IR was less functionally active in cells expressing IR-FKBP treated with AP1903 (Fig. 4g, h and Supplementary Fig. 19). Taken together, these results indicate that a decrease in insulin receptor cluster dynamics can produce a decrease in IR activity.

### High ROS levels promote IR cluster dysregulation

Several observations led us to test the hypothesis that high levels of reactive oxygen species (ROS) contribute to dysregulated IR clusters in insulin-resistant cells. Insulin stimulation causes a transient increase in H_2_O_2_ levels^70–75^. Many cell-extrinsic factors that promote insulin resistance, including hyperinsulinemia, TNFα, and high nutrients lead to excessive production of ROS^19,76–78^ (Supplementary Fig. 7c). Insulin-resistant cells and patients with T2D have been shown to have elevated levels of ROS^43,44^ and high ROS is a known cell-intrinsic factor that promotes insulin resistance^19,76,79,80^. Metformin has been proposed to decrease ROS levels by multiple mechanisms, including inhibition of the mitochondrial complex I respiratory chain^45^, inhibition of the redox shuttle enzyme mitochondrial glycerophosphate dehydrogenase^42^, and upregulation and activation of antioxidants^81^. Importantly, oxidative stress has previously been shown to affect the dynamic behaviors of other cluster-forming proteins^82–87^.

**Fig. 5:**
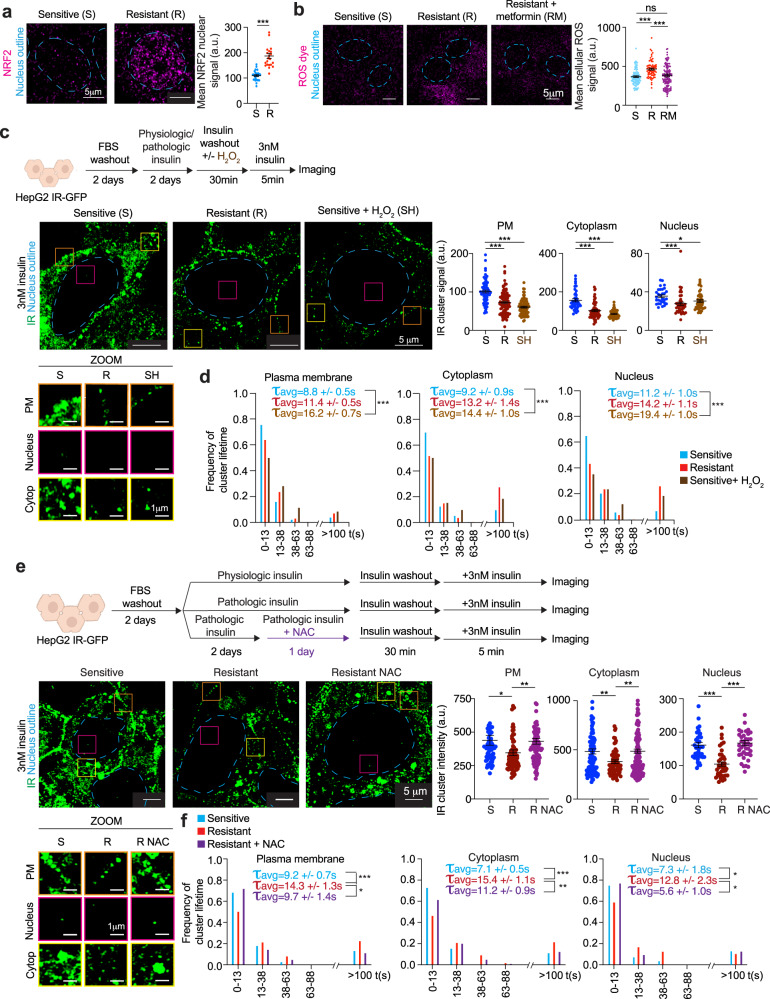
High ROS levels promote IR cluster dysregulation. **a** Representative immunofluorescence images for NRF2 (magenta) in insulin-sensitive or resistant cells (left). Dashed light blue lines represent the nuclear outline. Quantification of mean NRF2 signal intensity in nuclei of insulin-sensitive (sensitive, S) or resistant (resistant, R) cells (right). Data are represented as mean + /− SEM. The number of cells analyzed: insulin-sensitive 27 cells and insulin-resistant 22 cells. Unpaired two-sided *t* test was used for statistical analysis. **b** Representative images of cells treated with ROS-sensitive dye (left). Dashed light blue lines represent the nuclear outline. Quantification of mean ROS signal in insulin-sensitive (sensitive, S), insulin-resistant (resistant, R), and metformin-treated insulin-resistant cells (resistant + metformin, RM) (right). Metformin concentration used was 12.5 μM. Data are represented as mean + /− SEM. The number of cells analyzed: sensitive 107 cells, resistant 70 cells, resistant + metformin 134 cells. Unpaired two-sided *t* test was used for statistical analysis. **c** Schematic of cell treatments (top). Representative images of IR-GFP in insulin-sensitive cells (sensitive, S), insulin-resistant cells (resistant, R) or insulin-sensitive cells treated with H_2_O_2_ (sensitive + H_2_O_2_, SH) (middle left). Dashed light blue lines represent the nuclear outline. Orange, magenta, and yellow boxes represent regions at the plasma membrane (PM), nucleus, and cytoplasm (Cytop), respectively, that are magnified at the bottom (ZOOM). Scale bars are indicated. Quantification of IR-GFP signal intensity in IR clusters at the plasma membrane (PM), cytoplasm, and nucleus in insulin-sensitive (S), insulin-resistant (R), and H_2_O_2_-treated insulin-sensitive (SH) cells (right). Data are represented as mean + /− SEM. The number of clusters analyzed: sensitive plasma membrane 68 clusters, cytoplasm 40 clusters, nucleus 30 clusters; resistant plasma membrane 96 clusters, cytoplasm 60 clusters, nucleus 37 clusters; sensitive + H_2_O_2_ plasma membrane 79 clusters, cytoplasm 45 clusters, nucleus 44 clusters. Unpaired *t* test was used for statistical analysis. **d** Frequency of IR cluster lifetime in insulin-sensitive (sensitive, light blue), insulin-resistant (resistant, red), and H_2_O_2_-treated insulin-sensitive (sensitive + H_2_O_2_, brown) cells. The average lifetime (τ_avg_) of short-lived IR clusters + /− SEM is reported. The number of short-lived clusters analyzed: sensitive plasma membrane 432 clusters, cytoplasm 247 clusters, nucleus 181 clusters; resistant plasma membrane 547 clusters, cytoplasm 154 clusters, nucleus 222 clusters; sensitive + H_2_O_2_ plasma membrane 564 clusters, cytoplasm 379 clusters, nucleus 314 clusters. Unpaired two-sided *t* test was used for statistical analysis. **e** Schematic of cell treatments (top). Representative images of IR-GFP in insulin-sensitive cells (S, sensitive), insulin-resistant cells (R, resistant), or insulin-resistant cells treated with NAC (R NAC, resistant NAC) (middle left). Dashed light blue lines represent the nuclear outline. Orange, magenta, and yellow boxes represent regions at the plasma membrane (PM), nucleus, and cytoplasm (Cytop), respectively, that are magnified at the bottom (ZOOM). Scale bars are indicated. Quantification of IR-GFP signal intensity in IR clusters in insulin-sensitive (S), resistant (R), and NAC-treated insulin-resistant (R NAC) cells (middle right). Data are represented as mean + /- SEM. The number of IR clusters analyzed: sensitive plasma membrane 66 clusters, cytoplasm 109 clusters, nucleus 40 clusters; resistant plasma membrane 74 clusters, cytoplasm 73 clusters, nucleus 40 clusters; resistant NAC plasma membrane 91 clusters, cytoplasm 183 clusters, nucleus 41 clusters. Unpaired two-sided *t* test was used for statistical analysis. **f** Frequency of IR cluster lifetime in insulin-sensitive (sensitive, light blue), insulin-resistant (resistant, red), and NAC-treated insulin-resistant (resistant + NAC, purple) cells. The average lifetime (τ_avg_) of short-lived IR clusters + /− SEM is reported in the graphs. The number of short-lived clusters analyzed: sensitive plasma membrane 143 clusters, cytoplasm 168 clusters, nucleus 53 clusters; resistant plasma membrane 159 clusters, cytoplasm 309 clusters, nucleus 47 clusters; resistant + NAC plasma membrane 94 clusters, cytoplasm 232 clusters, nucleus 31 clusters. Unpaired two-sided *t* test was used for statistical analysis for all comparisons, except for nucleus sensitive vs resistant for which unpaired one-sided *t* test was used for statistical analysis. Source data are provided as a Source Data file.

To test this idea, we first determined if insulin-resistant cells are subjected to higher levels of oxidative stress than insulin-sensitive cells. Imaging of NRF2, a marker of oxidative stress^88^, revealed that insulin-resistant cells experienced higher levels of oxidative stress than insulin-sensitive cells (Fig. 5a). Quantification of ROS using a ROS-sensitive dye revealed that ROS levels were higher in insulin-resistant cells and, furthermore, that metformin treatment of these cells reduced ROS levels to those found in insulin-sensitive cells (Fig. 5b).

If oxidative stress causes IR cluster dysregulation, then treatment of insulin-sensitive cells with concentrations of an oxidizing agent known to cause oxidative stress might be expected to phenocopy the effects seen with insulin resistance. Similarly, if metformin acts by relieving the effects of oxidative stress, treatment of insulin-resistant cells with a reagent that reduces oxidative stress might phenocopy the effects of metformin. Indeed, we found that treating insulin-sensitive cells for 30 min with a concentration of H_2_O_2_ known to cause oxidative stress^89^ caused a reduction in the incorporation of IR into clusters with insulin stimulation and altered IR cluster dynamics, phenocopying the IR cluster dysregulation seen in insulin-resistant cells (Fig. 5c, d and Supplementary Fig. 20a). Furthermore, treatment of insulin-resistant cells with clinically relevant concentrations of N-acetyl cysteine (NAC)^90,91^ partially rescued the dynamic behavior of IR clusters (Fig. 5e, f and Supplementary Fig. 20b). Together, these results suggest that chronic hyperinsulinemia leads to excess levels of ROS in insulin-resistant HepG2 cells, that high levels of ROS alter IR incorporation into clusters, and that antioxidants can partially rescue the behavior of IR-clusters as a consequence of reducing ROS levels.

## Discussion

**Fig. 6:**
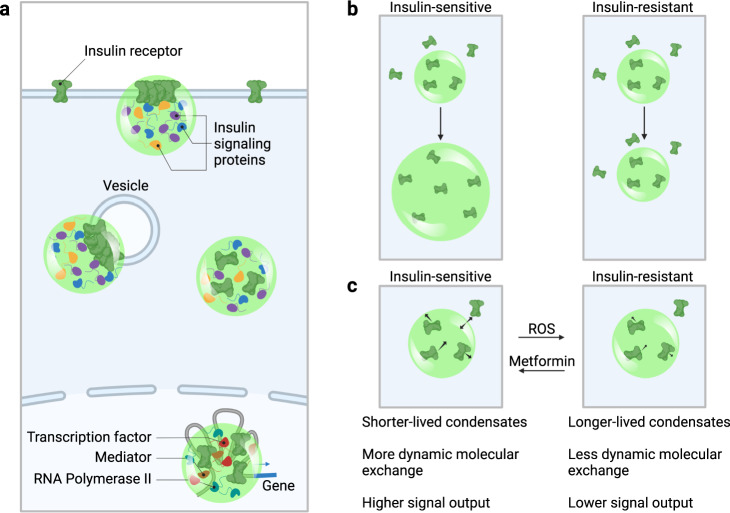
A proposed condensate model for insulin signaling and resistance. **a** Insulin receptor (green) is incorporated into condensates at the plasma membrane, at vesicle membranes, in the cytosol, and in the nucleus, together with other insulin signaling proteins and, in the nucleus, with proteins involved in transcription (Transcription factors, Mediator, RNA Polymerase II). **b** Insulin stimulation promotes IR incorporation into condensates in insulin-sensitive cells, and this effect is attenuated in insulin resistance. **c** In insulin-resistant cells, IR condensates are longer lived and have less dynamic molecular exchange than those in insulin-sensitive cells, and this difference in IR condensate dynamics correlates with signal output.

Recent studies have shown that the components of diverse signaling pathways, including those involving receptor tyrosine kinases, T-cell receptor, WNT, TGF-β, and JAK/STAT, involve the assembly of protein molecules into condensates at the plasma membrane, in the cytoplasm and nucleus^24–33^. Our evidence indicates that this is also the case for the insulin receptor, as the IR clusters observed here have characteristics expected of condensates. IR clusters form punctate bodies in cells^34,92^, undergo fusion, fission, and deformation^62–67^, and typically exhibit the short lifetimes described for other signaling condensates^62^, so we propose that these IR clusters are biomolecular condensates (Fig. 6).

Our results reveal that IR is incorporated into clusters at the plasma membrane, in the cytoplasm and in the nucleus. Acute insulin stimulation promotes further incorporation of IR into clusters in insulin-sensitive cells. In insulin-resistant cells, however, the ability of insulin stimulation to promote further IR incorporation into clusters is attenuated. Furthermore, IR cluster dynamics is altered in the insulin-resistant cells, but can be rescued with metformin treatment. In insulin-resistant cells, prolonged elevation of ROS levels appears to account for altered IR cluster dynamics because it can be phenocopied by H_2_O_2_ treatment of insulin-sensitive cells and rescued by NAC treatment of insulin-resistant cells. Metformin likely rescues IR cluster dynamics in insulin-resistant cells by reducing ROS levels.

We find that prolonged elevation of ROS levels in chronic hyperinsulinemia reduces the ability of insulin to promote further incorporation of IR molecules into clusters and extends the lifetime of IR molecules within the existing clusters. The known effects of ROS on proteins provides a mechanism to explain these findings. Transient insulin-induced H_2_O_2_ formation is essential for mediating insulin signaling^70–75^, but ROS can cause protein oxidation, which can alter protein conformation and change the ability of proteins to be incorporated into clusters^82,93^. It is possible that ROS-induced alteration of proteins may be a common mechanism in the pathogenesis of insulin resistance-associated diseases, including T2D, metabolic syndrome, non-alcoholic fatty liver disease (NAFLD), polycystic ovarian syndrome (PCOS), and Alzheimer’s disease. Indeed, metformin has been shown to decrease ROS production and improve patient outcomes in T2D and other diseases characterized by high ROS levels^42,81,94–96^. The mitochondrial respiratory chain complex 1 has been reported to be the primary target of metformin^45^, but it may be the reduction in ROS levels and the consequent benefit to protein cluster dynamics that is key to normal IR signaling.

The proposal that insulin resistance is associated with IR cluster dysfunction is consistent with prior evidence that implicates defects of insulin signaling pathways in hepatocytes in vivo^20^ and specific cellular stresses in both insulin resistance and cluster dysregulation. Some systemic and intracellular stresses that have been reported to induce insulin resistance, including oxidative stress and mitochondrial dysfunction, have independently been shown to influence the formation or behavior of other cellular condensates^93,97,98^. Further study of the molecular components of IR clusters and their oxidative modification should provide more detailed insights into the physicochemical properties that are altered in clusters by oxidative stress and mitochondrial dysfunction.

The work presented here proposes a mechanism that may explain, in part, how attenuation of insulin signaling occurs at a physicochemical level under conditions of insulin resistance in cells. Insulin resistance is a complex phenotype that can be considered at different scales—from cell, to tissue, to whole organism—and insulin resistance-related phenotypes will likely be best understood as the net result of cell and organism-level effects working together over time^1,2,19,99^. For example, while attenuated insulin signaling in insulin-resistant liver creates the expected effects on metabolic processes like gluconeogenesis and fat accumulation at the cellular level, cross-talk in the form of increased flux of metabolic substrates from insulin-resistant adipose tissue appears to have additive or compensatory effects, thus resulting in the final net phenotypes of hepatocyte gluconeogenesis and fat accumulation^20,100–107^. For these reasons, cell-based models may provide valuable insights into the mechanisms of dysregulated insulin signaling, but it will be important to integrate insights from these models with those obtained with organism-level studies for a more complete understanding of the insulin-resistant state.

The model described here for IR dysfunction has implications for the development of novel therapeutics for T2D. For example, the assays described here might be leveraged to develop new therapeutics that improve clinical outcomes for patients who cannot tolerate metformin or become resistant to the drug with prolonged use. Such therapeutics might also provide benefits to patients with other diseases where condensate dysregulation is also thought to play a role^97,108,109^.

## Methods

This research complies with all relevant ethical regulations: IRB 1999P004983 and IRB 2019P001245, COUHES E3272 and COUHES E3665. Informed consent was obtained by participants, and there was no participant compensation.

### Human liver donor samples

Samples of human livers were purchased from BioIVT or shared by collaborators at MGH (Hannah K. Drescher and Lea M. Bartsch). Informed consent was obtained by BioIVT or MGH from all human research participants. Sample ID numbers and donor information are obtained from either BioIVT or MGH and reported in Supplementary Table 1. Frozen samples were embedded in OCT compound (Tissue-Tek, 4583), re-frozen on dry ice, and stored at −80 °C. Embedded samples were sectioned using the cryostat at the W.M. Keck Microscopy Facility, MIT. Sectioning was performed at −21 °C to generate 10 μm-thick slices that were then placed on a Superfrost Plus VWR Micro Slides (VWR, 48311-703) and stored at −20 °C. Images of hematoxylin and eosin (H&E)-stained liver tissue were obtained from BioIVT.

IRB 1999P004983 and IRB 2019P001245, COUHES E3272 and COUHES E3665.

### Cell culture

*HepG2 cells* (ATCC HB-8065™) were used because of their demonstrated utility in the study of insulin signaling and resistance, and because they are amenable to genetic modification^11,46,47^. HepG2 cells were cultured in EMEM (ATCC 30-2003) supplemented with 10% FBS (Sigma Aldrich, F4135) at 37 °C with 5% CO_2_ in a humidified incubator. For passaging, cells were washed in PBS (Gibco, 10010-023) and TrypLE Express Enzyme (Life Technologies, 12604021) was used to detach cells from plates and dissociate cell clumps. To ensure proper cell dissociation, cells were incubated with TrypLE at 37 °C with 5% CO_2_ in a humidified incubator for 5 min; they were then mechanically dissociated by pipetting them up and down 8 times using a 5-ml serological pipette attached to an unfiltered 200-μl pipette tip. The 5-min incubation and mechanical dissociation were repeated one more time. TrypLE was quenched with EMEM supplemented with 10% FBS and cells were plated in new tissue culture-grade plates.

*HEK293T cells* (ATCC, CRL-3216) were used for the production of purified IRb protein. HEK293T cells were cultured in DMEM (GIBCO, 11995-073) supplemented with 10% FBS (Sigma Aldrich, F4135), 2 mM l-glutamine (Gibco, 25030), and 100 U/ml penicillin–streptomycin (Gibco, 15140), at 37 °C with 5% CO_2_ in a humidified incubator.

*Primary pre-adipocytes* from (ATCC, PCS-210-010) were cultured in in Fibroblast Growth Kit-Low Serum (ATCC PCS-201-041), as per the manufacturer’s instructions. Cells for experiments were dissociated and plated at 18,000 cells/cm^2^. Two days later, pre-adipocytes were differentiated in adipocyte differentiation media (ATCC PCS-500-050), as per the manufacturer’s instructions. Briefly, cells were washed, and medium was replaced with adipocyte differentiation initiation medium. After 48 h, half the medium was replaced. At day 4, medium was changed to adipocyte differentiation maintenance medium, and replaced every 3 days. At day 12, cells were rinsed and incubated in DMEM (Thermo Fisher, 11885084) with 0.1 or 3 nM of insulin for 5 days, replacing medium every other day. In the last 24 h, 12.5 μM metformin was added. On day 17, cells were prepared for the assays by rinsing, and 30 min of washing. Afterward, for imaging, cells were exposed to 3 nM insulin for 5 min, rinsed with PBS, and fixed in 4%PFA for 15 min at room temperature. For pAKT ELISAs cells were exposed to 3 nM insulin for 15 min and harvested in cell lysis buffer (Cell Signaling Technology, #9803) with phosphatase inhibitor (Thermo Fisher Scientific, 78442).

*For human liver spheroids*, primary human hepatocytes from a 50-year-old male donor (BioIVT; lot #SMC) were used. Cells were thawed in Cryopreserved Hepatocyte Recovery Media (CHRM, ThermoFisher), spun down at 100×*g* for 8 min, and resuspended in seeding medium (William’s E with 5.5 mM glucose, 2 mM GlutaMax, 15 mM HEPES, 5% FBS, 1% Pen/Strep, 100 nM hydrocortisone, and insulin 200 pM or 800 pM corresponding to the proper experimental group). Spheroids were formed using custom alginate microwells. In brief, 120,000 cells were seeded per well and spun at 50×*g* for 2 min to seed microwells, and cultured in a volume of 300 μl seeding medium. After 24 h, cells were switched to maintenance media for the remainder of the experiment. This maintenance media was composed of William’s E plus 6.25 µg/ml transferrin, 6.25 ng/ml selenium, 0.125% fatty acid-free BSA, 20 μM linoleic acid, 5.5 mM glucose, 2 mM GlutaMax, 15 mM HEPES, 0.5% Pen/Strep, and 100 nM hydrocortisone. Insulin was supplemented with concentrations adjusted to mimic healthy and disease-inducing states, either 200 pM for physiologic or 800 pM for pathologic insulin levels. Media was exchanged every 48 h throughout the experiment.

### Endogenously tagged cell line generation

A CRISPR/Cas9 system was used to generate genetically modified HepG2 cell lines. Target sequences were cloned into a plasmid containing sgRNA backbone, a codon-optimized version of Cas9 and mCherry. For IR targeting, two Cas9 gRNAs were used. For the generation of the IR-mEGFP, IR-Dendra2, and IR-Dendra2-FKBP endogenously tagged lines, homology-directed repair templates were cloned into pUC19 using NEBuilder HiFi DNA Assembly Master Mix (NEB, E2621S). For IR-mEGFP and IR-Dendra2 cell lines, the homology repair template consisted of mEGFP or Dendra2 cDNA sequence flanked on either side by 800 bp homology arms amplified from HepG2 genomic DNA using PCR (Supplementary Fig. 7a). For the IR-Dendra2-FKBP cell line, the homology repair template consisted of Dendra2 cDNA sequence followed by four FK506 binding protein (FKBP) binding domains^69^ flanked on either side by 800-bp homology arms amplified from HepG2 genomic DNA using PCR. The following sgRNA sequences with PAM sequence in parentheses were used for CRISPR/Cas9 targeting:

sgRNA_IR_C-term_1: CACGGTAGGCACTGTTAGGA(AGG)

sgRNA_IR_C-term_2: TAGGCACTGTTAGGAAGGAT(TGG)

To generate genetically modified cell lines, 2 × 10^6^ cells were transfected with 500 ng of Cas9 plasmid 1, 500 ng of Cas9 plasmid 2, and 1000 ng of non-linearized homology repair template using Lipofectamine 3000 (Invitrogen, L3000). Cells were sorted 48 h after transfection for the presence of the mCherry fluorescent protein encoded on the Cas9 plasmid to enrich transfected cells. This population of cells was allowed to expand for 1.5 to 2 weeks before sorting a second time for the presence of mEGFP or Dendra2, and single cells were plated into individual wells of a 96-well plate. The single cells were cultured in conditioned EMEM media (described below) for 1–1.5 months. In total, 20–30 colonies were screened for successful targeting using PCR genotyping to confirm insertion. PCR genotyping was performed using Phusion polymerase (Thermo Scientific, F531S). Using the following primers, PCR products were amplified according to the manufacturer’s specifications:

IR_fwd: GGAGAATGTGCCCCTGGAC

IR_rev: TTGGTAACCAAACGAGTCCACCT

To make conditioned media, we cultured HepG2 cells in fresh EMEM media (ATCC, 30-2003) supplemented with 10% FBS (Sigma Aldrich, F4135) for 3 days and saved the media (old EMEM media). The composition of conditioned EMEM media is as follows: 50% fresh EMEM media and 50% old EMEM media. The conditioned media was filter sterilized prior to use.

HepG2 cells expressing IR-mEGFP were used for super-resolution microscopy with LSM880 or LSM980 with an Airyscan detector.

HepG2 cells expressing IR-Dendra2 were used for single-molecule super-resolution microscopy, Dendra2 is a green-to-red photo-switchable protein that allows for single-molecule imaging.

### Constructs

For experiments with induced reduction in IR cluster dynamics, the vector used in this assay was modified from pJH135_pb_MCPx2_mCherry_rTTA vector^110^. IR-mEGFP-FKBP, which consists of the insulin receptor cDNA, flexible linker 1, mEGFP, flexible linker 2, and four copies of FKBP, was cloned into PmeI and NheI digested pJH135_pb_MCPx2_mCherry_rTTA using Gibson cloning by following the manufacturer’s instructions (NEB, E2621S). This vector is called pJP204_pb_TetON_INSR_2A_GFP_Dmbr4.

### Cell treatments

For insulin sensitivity and resistance experiments in HepG2 cells, cells were washed once with EMEM alone, without any supplements (ATCC, 30-2003), and cultured in EMEM for 2 days. Cells were then treated for two days with either physiologic (0.1 nM) or pathologic (3 nM) levels of insulin (Sigma Aldrich, I9278-5ML) in EMEM supplemented with 1.25% fatty acid-free bovine serum albumin (BSA; Sigma Aldrich, A8806-5G). Media was replenished every 12 h. To wash out insulin, cells were washed with EMEM seven times, including three quick washes, three 5 min washes, and a long 20 min wash in EMEM at 37 °C. In order to investigate insulin response, cells were acutely treated for 5 min with insulin diluted in EMEM supplemented with 1.25% fatty acid-free BSA at 37 °C with 5% CO_2_ in a humidified incubator. The concentration of insulin used varied and is reported in the figures.

For TNFα treatment, cells were cultured in EMEM BSA containing 10 pg/ml^111^ of Human TNF-alpha Recombinant Protein (Thermo Fisher Scientific, PHC3016) for 2 days. Media was replenished every 12 h. Insulin washout and insulin stimulation were performed as above.

For high-nutrient conditions, cells were cultured for 2 days in EMEM containing either: (1) 10 mM glucose, 45 μM oleic acid (Cayman Chemical, 29557), 30 μM palmitic acid (Cayman Chemical, 29558) and 3 nM insulin (called in the text “pathologic glucose, pathologic fat, and pathologic insulin (GFI)”) or (2) 10 mM glucose, 45 μM oleic acid, 30 μM palmitic acid and 0.1 nM insulin (called in the text “pathologic glucose, pathologic fat, and physiologic insulin (GF)”). Control cells were cultured for 2 days with EMEM containing BSA control (Cayman Chemical, 29556). Media was replaced every 12 h. Insulin washout and insulin stimulation were performed as above.

For metformin treatment, metformin (Sigma Aldrich, D150959-5G) was resuspended in sterile water to a concentration of 1 M and diluted in cell media to the reported concentrations. Insulin-resistant cells were treated with pathologic concentrations of insulin and metformin at various concentrations reported in the figures. Media was replenished every 12 h. Insulin washout and insulin stimulation were performed as above.

For N-acetyl cysteine treatment, insulin-resistant cells were treated with pathologic concentrations of insulin and 1 mM N-acetyl cysteine (Sigma Aldrich, A9165-25G) for 24 h. Media was replenished every 12 h. Insulin washout and insulin stimulation were performed as above.

For oxidative stress, insulin-sensitive cells were treated with 20 mM H_2_O_2_ (Sigma Aldrich, H1009) for 30 min. Insulin stimulation was performed as above.

For adipocytes, following differentiation, cells were cultured with EMEM for 2 days and with EMEM containing either physiologic (0.1 nM) or pathologic (3 nM) concentrations of insulin for 2 days. Cells were then cultured with EMEM containing either physiologic (0.1 nM) or pathologic (3 nM) of insulin or with pathologic (3 nM) concentrations of insulin and 12.5 μM of metformin for 1 day. Cells were then washed with EMEM and acutely stimulated with or without 3 nM insulin for 5 min prior to cell collection for immunofluorescence or ELISA.

For experiments that forced reduction in IR cluster dynamics, 1 × 10^5^ cells/cm^2^ HepG2 cells were transfected with 0.07 μg/cm^2^ pJP204_pb_TetON_INSR_2A_GFP_Dmbr4 using Lipofectamine 3000 (Invitrogen, L3000). On day 2, the cells were treated with 100 ng/ml doxycycline (Sigma, D9891-5G). On day 3, the cells were treated with EMEM, 100 ng/ml doxycycline containing either 5 μM AP1903 (MedChemExpress, NC1416062) or 5 μM DMSO (Sigma, D2650-100ML) for 16 h and then harvested for imaging and western blot.

### Cell viability

Cells were detached from plates and dissociated from clumps using TrypLE as described above. TrypLE was quenched with EMEM supplemented with 10% FBS. Dead cells were stained with trypan blue (Life Technologies, T10282), and the percentage of cell viability was then measured using the Countess II FL (Applied Biosystems, A27977) according to the manufacturer’s specifications.

### Insulin clearance

Insulin-sensitive HepG2 cells were cultured in EMEM for 30 min and then in 3 nM insulin for 0, 5, or 24 h. Culture media was collected and insulin concentration was measured at all timepoints using Human/Canine/Porcine Insulin DuoSet ELISA kit (R&D Systems, DY8056-05) according to the manufacturer’s specifications. Clearance fraction was calculated by dividing the measured insulin concentration in cultured media by the measured insulin concentration in the cell-free control wells.

Insulin clearance in human liver spheroids was evaluated by collecting media after 48 h in culture media was removed and insulin concentration was measured using Human/Canine/Porcine Insulin DuoSet ELISA kit (R&D Systems, DY8056-05) according to the manufacturer’s specifications. Clearance fraction was calculated by dividing the measured insulin concentration in cultured media by the measured insulin concentration in the cell-free control wells.

### Glucose production

Insulin-sensitive and -resistant cells were cultured in EMEM for 30 min, and then cells were treated with 0, 0.1, 1, and 10 nM insulin in glucose production media, containing DMEM (Thermo Fisher Scientific, A1443001), 15 mM HEPES (Gibco, 15630-080), 1 mM pyruvate (Sigma Aldrich, P5280), 20 mM lactate (Sigma Aldrich, L7022-5G) for 4–5 h. Media was removed and glucose production was measured using Amplex^TM^ Red Glucose/Glucose oxidase assay kit (Thermo Fisher Scientific, A22189) according to the manufacturer’s specifications.

Measurement of glucose production in human liver spheroids was performed as follows. At day 10 in culture, spheroids were washed five times with glucose-free William’s E media (Thermo Fisher, ME18082L1), followed by culture for 24 h in glucose-free William’s E maintenance media, supplemented 1 mM pyruvate, 20 mM lactate, and between 0 to 10 nM insulin stimulation. After 24 h, media was collected and glucose quantified with the Amplex Red Glucose Assay Kit (Thermo Fisher, A22189) according to the manufacturer’s instructions.

### Albumin quantification

To assess hepatocyte spheroid function, media was collected during every media exchange, and albumin secretion was assayed via ELISA kit (Bethyl Laboratories, E80-129) following the manufacturer’s instructions.

### siRNA experiments

HepG2 cells were reverse transfected using Lipofectamine^TM^ RNAiMAX Transfection reagent (Thermo Fisher Scientific, 13778100) following the manufacturer’s instructions. Cells were dissociated using TrypLE as previously described and then seeded in six multiwells in 1 ml EMEM supplemented with 10% FBS and the transfection reagent. Cells were cultured with the transfection reagent for 2–3 days prior to collection for western blot and immunofluorescence.

The INSR siRNA pool (Dharmacon Inc, L-003014-00-0005) and the ON-TARGETplus Non-targeting Control Pool (Horizon Discovery, D-001810-01-05) were used.

### Western blot

Cells were washed with ice-cold PBS (Life Technologies, AM9625) and lysed in Cell Lytic M (Sigma Aldrich C2978) supplemented with protease and phosphatase inhibitors (Sigma Aldrich, 11873580001 and 4906837001) directly on the wells. Lysates were placed into a 1.5-ml tube and mixed at 4 °C for 20 min, sonicated, and then centrifuged at 12,000×*g* for 15 min. Supernatant was collected, and protein concentration was determined using a BCA Protein Assay Kit (Life Technologies, 23250) according to the manufacturer’s instructions. Equal amounts of protein (5–50 µg per sample) were separated on 10% or 12% Bis-Tris gels in 5% XT MOPS running buffer (Bio-Rad Laboratories, 1610788) at 100 V until the dye front reached the end of the gel. Protein was then transferred to a 0.45-µm PVDF membrane (Millipore, IPVH00010) in ice-cold transfer buffer (25 mM Tris, 192 mM glycine, 20% methanol) at 300 mA for 1 h or 250 mA for 2 h at 4 °C. After the transfer, membranes were blocked in either 5% nonfat milk (LabScientific, M0842) dissolved in TBST (2% Tris-HCl pH 8.0, 1.3% 5 M NaCl, 0.05% Tween 20) or 5% BSA (VWR, 102643-516) in 1× TBST for 15 min to 1 h at room temperature with shaking. Membranes were then incubated overnight at 4 °C in 1:1000 primary antibody (specific antibodies listed below) in 5% nonfat milk in TBST or 5% BSA in TBST. BSA was used for immunoblotting phosphorylated proteins, otherwise milk was used. Membranes were then washed three times for 5 min in TBST shaking at room temperature prior to incubation in 1:10,000 secondary antibody (specific antibodies listed below) in 5% nonfat milk in TBST for 1 h at room temperature. This was followed by three 10 min washes in TBST. Membranes were developed with ECL substrate (Thermo Scientific, 34080) and imaged using a CCD camera (BIO RAD, 1708265). Immunoblot quantification was performed using the “analyze gel” tool on Fiji/ImageJ v2.1.0/153c.

The following primary antibodies were used for WB: anti-phosphorylated insulin receptor (Abcam, ab60946; Cell Signaling, 3026, dilution 1:1000), anti-insulin receptor beta (Cell Signaling, 23413 dilution 1:1000; Bethyl, A303-712A; Cell Signaling, 3025 dilution 1:1000), anti-insulin receptor alpha (Cell Signaling, 74118 dilution 1:1000), anti-phosphorylated IRS1 (Cell Signaling, 3070 dilution 1:1000), anti-IRS1 (Cell Signaling, 2382 dilution 1:1000), anti-phosphorylated AKT (Cell Signaling, 4056 dilution 1:1000), anti-AKT (Cell Signaling, 9272 dilution 1:1000), anti-phosphorylated ERK (Cell Signaling, 4377 dilution 1:1000), anti-ERK (Cell Signaling, 9102 dilution 1:1000), anti-pGSK a,β (Cell Signaling, 8566 dilution 1:1000), anti-GSK a,β (Cell Signaling, 4337 and 12456 dilution 1:1000), anti-beta Actin (Sigma Aldrich, A5441 dilution 1:10,000), and anti-GAPDH (Abcam, ab8245 dilution 1:1000). The following secondary antibodies were used: donkey anti-rabbit IgG (Cytiva Life Sciences, NA934-1ML, dilution 1:10,000) and sheep anti-mouse IgG (Sigma Aldrich, NXA931V, dilution 1:10,000).

For quantitative western blot analysis, equal numbers of cells were cultured in each well on a six-well plate. To estimate the number of cells per well, cells in two wells were dissociated with TrypLE (Life Technologies, 12604021) and counted using the Countess II (Applied Biosystems, A27977). Cells from another well were lysed on the plate as described above. A dilution series of purified IRb-mCherry and HepG2 cellular lysate was separated on 10% Bis-Tris gels in 5% XT MOPS running buffer. Immunoblotting was performed as above. Bands were quantitated using Fiji/ImageJ v2.1.0/153c, from which we calculated the estimated number of molecules of IRb per HepG2 cell.

### Proteolytic surface shaving experiment

To compare IR amounts in whole cells and at the plasma membrane, proteolytic surface shaving experiment was performed. Equal numbers of cells were cultured in each well of on a six-well plate and cultured with either 0.1 nM insulin or 3 nM insulin for 2 days. To estimate the relative IR amounts in the whole cell, cells were washed in PBS and lysed in ice-cold Cell Lytic M supplemented with protease and phosphatase inhibitors, as previously described. To estimate the relative IR amounts at the plasma membrane (labeled in the figure as “Digested”), cells were digested with TrypLE for 10 min at 37 °C and quenched with EMEM 10% FBS. Cells were spun down at 300×*g* for 5 min, washed in PBS and ice-cold Cell Lytic M supplemented with protease and phosphatase inhibitors. Samples were then processed for western blot as previously described and immunoblotted for insulin receptor alpha and beta-actin.

### Metabolomics

#### Metabolite isolation from liver tissue

Flash-frozen tissues were pulverized with a mortar and pestle in a liquid nitrogen bath. Tissue powder was transferred into Eppendorf tubes and resuspended in 800 µl ice-cold LC-MS grade 60:40 methanol:water (ThermoFisher). Samples were vortexed for 10 min at 4 °C. Then, 500 µl of ice-cold LC-MS grade chloroform (provided by the Metabolomics core) was added to the lysate and samples were vortexed for an additional 10 min at 4 °C. Samples were centrifuged at 16,000×*g* for 10 min at 4 °C, creating three layers: the top layer containing polar metabolites, the bottom layer containing non-polar metabolites, and the middle layer containing protein. The top layer was transferred to a new tube, dried down in a speedvac, and subsequently stored at −80 °C until they were analyzed by LC-MS.

#### Stable isotope tracing for lipogenesis in HepG2 cells

Cells were cultured with 0.1 nM or 3 nM insulin (Sigma Aldrich, I9278-5ML) for 2 days as detailed above. To wash out insulin, cells were washed with EMEM (ATCC 30-2003) seven times, including: three quick washes, three 5 min washes, and a long 20 min wash in EMEM at 37 °C. Cells were then cultured in EMEM supplemented with 1 mM sodium acetate-^13^C_2_ (Sigma Aldrich, 282014) and with either 0 nM insulin or 1 nM insulin for 36 h. Media was replenished after 24 h. Cells were then processed for metabolite isolation (see below).

#### Stable isotope tracing for gluconeogenesis in HepG2 cells

Cells were cultured with 0.1 nM or 3 nM insulin (Sigma Aldrich, I9278-5ML) for 2 days as detailed above. To wash out insulin, cells were washed with EMEM (ATCC 30-2003) seven times, including: three quick washes, three 5 min washes and a long 20 min wash in EMEM at 37 °C. Cells were washed with glucose-free RPMI (Gibco, 11879-020) then cultured in glucose-free RPMI for 3 h. Cells were then cultured in glucose-free RPMI supplemented with 5 mM sodium pyruvate-^13^C_3_ (Cambridge Isotope Laboratories, NC1345852) and 5 mM sodium l-lactate (Sigma Aldrich, L7022) and with either 0 nM, 1 nM, 10 nM, or 100 nM insulin (Sigma Aldrich, I9278-5ML) for 16 h. Cells were then processed for metabolite isolation (see below).

#### Metabolite isolation from HepG2 cells

cells were washed in ice-cold PBS (Life Technologies, AM9625), 500 µl of cold 80% MeOH (shared by the Metabolite Profiling Core Facility) was added per well of a six-well plate, and the plate was placed at −80 °C for at least 15 min. Following the −80 °C incubation, the plate was scraped on dry ice and the solution was transferred to a 1.5-ml tube and then vortexed for 5 min. To remove cellular debris, the samples were centrifuged at maximum speed for 10 min at 4 °C, and the supernatant was transferred to a new 1.5-ml tube on dry ice. To remove solvents, the samples were lyophilized using Refrigerated CentriVap Benchtop Vacuum Concentrator connected to a CentriVap-105 Cold Trap (Labconco). Metabolite pellets were resuspended in LC-MS grade water (ThermoFisher), and vortexed for 10 min at 4 °C. Samples were centrifuged at 16,000*×g* for 10 min at 4 °C and supernatant was moved into LC-MS vials. Liquid chromatography and mass spectrometry were performed by the Whitehead metabolomics core.

### Immunofluorescence

HepG2 cells, human liver spheroids, human primary adipocytes, and human tissue liver sections were fixed in 4% PFA (VWR, BT140770-10×10) in PBS (Life Technologies, AM9625) for 10 min at room temperature. Cells were washed three times for 5 min in PBS, permeabilized with 0.5% TritonX100 (Sigma Aldrich, X-100) in PBS, washed three times for 5 min in PBS, and then blocked with 4% IgG-free BSA (VWR, 102643-516) for 15–60 min at room temperature. Afterward, the cells were incubated with 1:500 or 1:1000 primary antibody (specific antibodies listed below) in 4% IgG-free BSA in PBS at 4 °C overnight. The next day, cells were washed three times with PBS and incubated with 1:500 or 1:1000 secondary antibodies (specific antibodies listed below) in 4% IgG-free BSA at room temperature for 1 hr covered in foil. Cells were washed three times with PBS for 5 min. DNA was stained using 1:5000 Hoechst (Thermo Fischer Scientific, 3258) in PBS for 5 min at RT. Cells were washed three times with PBS for 5 min, stored at 4 °C until imaging. For tissue sections, samples were mounted using Vectashield mounting media (Vector Laboratories, Inc, H-1000). LSM880 or LSM980 microscope with Airyscan detector (ZEISS) was used for image acquisition. Images were then processed using Fiji/ImageJ v2.1.0/153c.

Primary antibodies used were anti-insulin receptor beta (Cell Signaling, 23413), anti-NRF2 antibody (Abcam, ab62352, 1:500 dilution), anti-cytokeratin 18 (CK18) (Abcam, ab668, 1:500 dilution), anti-PI3K (Abcam, ab135253, ab62352, 1:500 dilution), anti-AKT (Cell Signaling, 2920, ab62352, 1:500 dilution), anti-clathrin (Abcam, ab24578, ab62352, 1:500 dilution), anti-LAMP1 (Abcam, ab25630, ab62352, 1:500 dilution), and anti-EEA1 (Abcam, ab70521, ab62352, 1:500 dilution), anti-pIRS1 (Abcam, ab4873, ab62352, 1:1000 dilution), anti-perilipin (Sigma, P1873, 1:500 dilution). Secondary antibodies used were Alexa Fluor 488 goat anti-rabbit IgG (Thermo Fischer Scientific, A11008), Alexa Fluor 647 goat anti-rabbit IgG (Thermo Fischer Scientific, A21244), Alexa Fluor 568 goat anti-mouse IgG (Thermo Fischer Scientific, A11031).

Images were acquired at LSM880 or LSM980 Microscope with Airyscan detector with ×63 objective using Zen Black software (ZEISS) at the W.M. Keck Microscopy Facility, MIT. Images were then processed using Fiji/ImageJ v2.1.0/153c. Scale bars were determined using Fiji/ImageJ v2.1.0/153c and, when scale bars were obscured by fluorescence intensity, a black background was added to improve visibility.

### Live-cell imaging

Cells expressing endogenous IR tagged with GFP were grown on 35-mm glass bottom dishes (MatTek Corporation, P35G-1.5-20-C). Cells were imaged at 37 °C using the LSM880 or LSM980 Microscope with Airyscan detector with 63x objective and Zen Black software (ZEISS) at the W.M. Keck Microscopy Facility, MIT. Images were then processed using Fiji/ImageJ v2.1.0/153c.

### ROS staining and live-cell imaging

After culturing the HepG2 cells with physiologic insulin concentrations or pathologic insulin concentrations or with TNFα or high nutrients for 2 days, media was removed, and cells were cultured with ROS Deep Red Stock Solution (Abcam, ab186029) diluted to 1× in Dulbecco’s PBS (Gibco, 14040-133). Cells were incubated at 37 °C with 5% CO_2_ in a humidified incubator for 30 min. Cells were imaged at 37 °C using the LSM880 Microscope with Airyscan detector with ×63 objective and Zen Black software (ZEISS) at the W.M. Keck Microscopy Facility, MIT. Images were then processed using Fiji/ImageJ v2.1.0/153c.

### RNA FISH

Pipettes and laboratory bench were treated with RNaseZap (Life Technologies, AM9780). Cells were fixed with 4% PFA (VWR, BT140770-10×10) in PBS (Life Technologies, AM9625) for 10 min at RT. Cells were washed three times with PBS for 5 min. Cells were permeabilized with 0.5% TritonX100 (Sigma Aldrich, X-100) in 1× RNase-free PBS (Invitrogen, AM9625) for 10 min at room temperature. Cells were washed three times with RNase-free PBS for 5 min. Cells were washed once with 20% Stellaris RNA FISH Wash Buffer A (Biosearch Technologies, Inc., SMF-WA1-60), 10% Deionized Formamide (EMD Millipore, S4117) in RNase-free water (Life Technologies, AM9932) for 5 min at room temperature. Cells were then hybridized with 90% Stellaris RNA FISH Hybridization Buffer (Biosearch Technologies, SMF-HB1-10), 10% Deionized Formamide, 12.5 µM Stellaris RNA FISH probes designed to hybridize intronic regions of each transcript (*FASN, SREBF1*, and *TIMM22*; probes listed below*)*. Hybridization was performed overnight at 37 °C. Cells were then washed twice with Wash Buffer A for 30 min at 37 °C and once with Stellaris RNA FISH Wash Buffer B (Biosearch Technologies, SMF-WB1-20) for 5 min at room temperature. Images were acquired at LSM880 or LSM980 Microscope with Airyscan detector with ×63 objective using Zen Black software (ZEISS) at the W.M. Keck Microscopy Facility, MIT.

Stellaris® FISH Probes, Custom Assay with TAMRA Dye (LGC Bioserch, SMF-1001-5).

*FASN* RNA FISH probe sequence:

cgagagcggaggatgaggag

aaggggcacgaacaccgaga

gaaatggggatagcctatgc

cattcagttcagggtattgg

acaaaggtggagatggagct

gtgcaatgtccaggaaggag

ctccgtaacaagcagatggg

gagcacatctggtatgcaac

tagagctgctctgtggaaga

tctaccatgctgactcacag

gactggtacgaccagatctg

caaacaatggagcaggctcc

aactgaggactctctgctat

gacaggcgggtgtttaaatc

tggggtgcagttgggaaact

atcagaagcgaggagactgg

ctcaagaacctcctgctttg

tggcgggagagggttgaaat

aggtcaaatgccagtcagag

gagcacctggatcttaagaa

gagcataaccttagtcttgg

aggagacaaaggccaagtgt

agaacaggccgtgataagga

aacaggaaccaggtcacaga

tgccacaaacagtggtcaag

cacactcacctgcaaggaag

tagtgggtcaggaagctgta

tgtgcctggtctctctaaag

gtgagtgatcatgggtgttg

ctgtggcctgaacactaagg

ctaattctgccatggcacag

caaggctctgcctagcaaag

gataggaaagaagcccctat

ggaacaggacactcaggttg

tctgagggaaccctgatgac

agagatgggtgccaacagag

gtggaggtgtcattacagag

gagccttcatgagaaaggtt

acaagtgtggtaatggcagc

aagggacctgaggacaaacc

caagggaaccgagagggaat

tattagtccacctggacatc

ctacggagaagggaggcatg

tgtggggagggaagagtctg

gggagagttggagatcagag

tggaaagggaggtgcggaag

agaagcaagtctggggtcaa

actgaggaacagtgaccagg

*SREBF1* RNA FISH probe sequence:

ttggctgtaagctgtgtgtc

ctaaataaacgaggctggcc

acctttattgaagaggcctg

gatgcagacagcagggtctg

cgggcatagggttagaatgt

tggagctcaataaagccagg

aaggctagagaagaggccag

gactagagctgaatgcaggg

ctttcaggaccagaggtaac

ctttgatggggctgggttag

ggactactgtagctcctaaa

aaaggctggatatgtgaccc

cgaaaggaacagagccagga

atgaggctcagaggatatgg

taggatctgttagggtcttc

cttacctatggacagaggga

agcaggaacaagggttgaca

gaggacgggacagattcatg

cagtcctacctgtggatgag

gagagagctgcagggataag

taagagagcacctgtagggg

ctgcatgtgcttctgaaagc

tcacttctccaattagccat

gagaagatgccattgttggc

gcttccaaaggaaaaccgac

ggtggacataactatcacca

ctgccaaggacaggggaaag

gagggcacaacgacacttac

gagctattctcagaatcccg

gaggaatgaagcgtgcatgg

ctgtcggaacagatggcagg

tcacctgtggaaggagagag

tgagaagggagccaggacag

aacaaaggctgagtgaggca

ccctgaggaaaaaaggtggt

cagaagagtgccagtcagac

ccagggaatggaaagctgaa

gaagccttagccaaaaagca

gcaatgcaacagcaatgcac

tgctgagcagacagcacatc

ttggtatcacatcccatgtg

ccactgattccttgtgaaag

gtatcccacaaatgacagtc

cacagactgagtcacgcacg

gtctcagcccacacacaaag

tgtacctggcacacaggtac

acgaggatgtgtcagggatg

*TIMM22* RNA FISH probe sequence:

tggtcttctcggcagagatc

agggtgtggtcaaggtcaag

acgcccgattcacgaacgag

attttcatcaggaaagccgg

cacagcaccgattctctaac

atgtaataactttcaggccc

tcacctggcacgtgatattg

tgaagaccaggctcttgtct

acatagtataatacaggccc

ttcctgctcaacattcttct

tgcatgtgcatttccatatg

aacaactgctgccctagagt

tctttcagtttttcaaacct

cggggctgtctagtcacaat

acaagtgcagacttcgtctc

ataaaatcatgggcacctcc

acctcatggtcaatatgagt

actgagcacatgccagtatg

gtgacattcacagtaatgct

ttgcaaatcactctcttggc

ccctcaacttcagctatcaa

attctctctacattctctca

aagattccatctttaaccct

ctgtcattccctaacatttc

ctccgtctctaagatttctt

gggtctatgttactgacatc

ttgcctggagacttgcaatc

ctcttgccactcaaactctc

atgcttttggatgaccaccg

tgggatcatcctggaaggga

catgttttctgcattactct

acagagatgaaggtgtcttt

agacacttacctagaagcaa

gcaaggatttcttagaaggc

aagtaatgaaatggtggccc

tgcttctgatttgctttcta

ctctccaataagtctcgttt

ccagcatttggaatgtaatc

caccagagtgctgaaaccaa

ttcatagatgcttcctgcag.

### Imaging analyses

Fiji/ImageJ v2.1.0/153c was used to quantify IR fluorescence intensity per cell for the IR antibody validation experiment. With the polygon selection tool, a polygon was drawn around a cell outline. The average fluorescence intensity in the polygon (= in the cell) was determined using the measure tool on Fiji/ImageJ v2.1.0/153c. The background was then subtracted by a threshold determined by averaging the background intensity in a rectangular region outside of the cells.

To manually quantify IR fluorescent signal in puncta and clusters, Fiji/ImageJ v2.1.0/153c was used. A circle or an oval was drawn around IR puncta using the oval selection tool, and the average fluorescence intensity in the circle or oval (= in the puncta) was determined using the measure tool on Fiji. The background was then subtracted as previously described. To quantify IR fluorescent signal in puncta and clusters in various cellular compartments, we identified the location of the plasma membrane, cytoplasm, and nucleus as follows. The plasma membrane location was identified based on IR immunofluorescence signal, IR-GFP fluorescent signal or CK18 immunofluorescence signal or cell edge. The nucleus was determined by the Hoechst stain for immunofluorescence and tc-PALM experiments. For IR-GFP experiments, Hoechst dye could not be used, because of bleed-through of the Hoechst fluorescence into the GFP channel confounded the identification of IR-GFP puncta. In these cases, the nuclear outline was inferred based on the very clear IR signal difference between the nucleus and the cytoplasm.

To computationally measure IR fluorescent signal in puncta and clusters, Airyscan images from all conditions were maximally-projected in the z-plane and background-subtracted by a threshold determined by averaging the background intensity in a rectangular region outside of the cells. For segmenting IR puncta, the images were first subtracted by a median-filtered image (10 px) and then subjected to a Laplace of Gaussian filter (sigma = 1). Filtered images were then thresholded on signal intensity (intensity > mean image intensity + 2*standard deviation of image intensity). Thresholded binary images were then subjected to a morphological opening operation with a 3 × 3 filled structuring element to remove small objects. The mean intensity of the background-subtracted raw image was then measured for each segmented puncta (c-in), and background intensity (c-out) was calculated from the mean intensity of an inverted mask of the called puncta.

To quantify pIRS1 fluorescent signal in IR puncta/clusters manually, Airyscan images were opened on Fiji as composite images. A circle or an oval was drawn around IR puncta using the oval selection tool, and the average fluorescence intensity of pIRS1 in the circle or oval (= in the puncta/clusters) was determined using the measure tool on Fiji.

To quantify pIRS1 fluorescent signal in IR puncta/clusters computationally using Fiji, 3D object counter tool was used. Briefly, images were thresholded on signal intensity and IR puncta/clusters were identified. 3D object counter tool then determined the intensity pIRS1 channel in the identified IR puncta. pIRS1 signal intensity was then background-subtracted by a threshold determined by averaging the background intensity in a rectangular region outside of the cells.

To estimate the number of IR puncta at the plasma membrane, cytoplasm, nucleus in an entire cell, IR puncta were initially counted at various cellular locations in a cell slice using Fiji as described above. The number obtained from the cell slice was then multiplied based on the estimated surface area of the plasma membrane, the volume of the cytoplasm, the volume of the nucleus or volume of the entire cell, which were obtained considering the length and width of the cell under investigation and the estimated height (~5 μm).

Quantification of the ROS dye fluorescence intensity per cell was performed using Fiji. Using the polygon selection tool on Fiji/ImageJ v2.1.0/153c, a polygon was drawn around a cell outline, which was identified by looking at the IR-GFP channel. The average ROS dye fluorescence intensity in the polygon (= in the cell) was determined using the measure tool on Fiji/ImageJ v2.1.0/153c.

Fusion, fission, or deformation events were identified in time-lapse images of the endogenously tagged IR-GFP HepG2 line. To confirm bona fide deformation, fusion or fission events, we quantified the total IR intensity before and after the event as a product of IR fluorescence intensity and area of the IR puncta. The total intensity was conserved in bona fide deformation, fusion, and fission events.

### ELISA

PathScan® Total Insulin Receptor β Sandwich ELISA kit (Cell Signaling, 7069) was used to quantify insulin receptor levels, PathScan Phospho-Akt2 (Ser474) and Total Akt2 Sandwich ELISA kits were used to quantify AKT2 levels (Cell Signaling Technology, #7048 and #7046, respectively) as per the manufacturer’s instructions, by colorimetric reading at 450 nm on a Thermo Fisher Multiskan Go plate reader.

### Chromatin immunoprecipitation-sequencing (ChIP-seq)

ChIP-seq experiments were performed by the Center for Functional Cancer Epigenetics (CFCE) at the Dana-Farber Cancer Institute. For ChIP-seq analysis, cells were cross-linked with 2 mM DSG (VWR, PI20593) for 45 min at room temperature, followed by fixation for 10 min with 1% formaldehyde (Tousimis Research Corporation, 1008A) at room temperature on a shaker at 850 rpm. Cross-linked nuclei were quenched with 0.125 M glycine (Sigma Aldrich, G7126) for 5 min at room temperature and washed with PBS (Life Technologies, AM9625) that contained protease inhibitor (Roche, 11836170001) and HDAC inhibitor sodium butyrate. After fixation, pellets were resuspended in 200 μl of 1% SDS, 50 mM Tris-HCl pH 8, 10 mM EDTA and sonicated in 1 ml AFA fiber millitubes (Covaris, 520135) for 25 min using a Covaris E220 instrument (setting: 140 peak incident power, 5% duty factor and 200 cycles per burst) 600 s per sample. Chromatin was diluted 5 times with ChIP Dilution buffer (1%Triton X-100, 2 mM EDTA pH 8, 150 mM NaCl, 20 mM Tris-HCl pH 8) and was immunoprecipitated with 10 μg of primary antibody against IR (Bethyl, A303-712A) and Dynabeads® Protein A/G (Thermo Fisher, 10015D). ChIP-seq libraries were constructed using NEBNext UltraTM II kit (NEB, E7645S) according to the manufacturer’s specifications. 75-bp paired-end reads were sequenced on a NextSeq instrument. In all, 75-bp single-end reads were sequenced on an Illumina NextSeq instrument.

MED1 ChIP-seq was used from GEO: GSM2040029

RPB1 ChIP-seq was used from GEO: GSM2864931 *(14)*.

### ChIP-seq analysis

ChIP-seq bioinformatics analysis for insulin receptor was performed on the Whitehead High-Performance Computing Facility using the nf-core ChIP-seq pipeline v1.2.1^112^ with Nextflow v20.04.1. Quality control of.fastq files was performed with FastQC v0.11.9. Trim Galore! v0.6.4_dev was used to trim low-quality reads. Alignment was performed against the hg19 genome assembly using BWA v0.7.17-r1188^113^. Peak calling was performed using MACS2 v2.2.7.1^114^. Preseq v2.0.3^115^ and MultiQC v1.9 were used for quality control. Browser tracks were prepared to represent reads per million per base pair (rpm/bp).

### RT-qPCR and RNA sequencing

RNA was extracted using TRIzol^TM^ reagent (Thermo Fisher Scientific, 15596026) following the manufacturer’s instructions. cDNA synthesis was performed using qScript cDNA Supermix (QuantaBio, 95048-500) according to the manufacturer’s instructions, using 1000 ng RNA as starting material.

qPCR was performed on a Thermo Fisher Scientific QuantStudio 6 machine using Fast SYBR™ Green Master Mix (Thermo Fisher, 4385618) and primers (listed below) according to the manufacturer’s instructions. Expression data are presented after calculating the relative expression compared with the housekeeping gene RPLP0, using the equation Relative Quantification (RQ) = 100/(2ˆ(Target Gene Ct – RPLP0 Ct). When data are reported relative to a sample condition, the condition of reference was set as 1 and the data of the other conditions were reported as a ratio (condition/condition of reference).

RNA sequencing was performed by the Whitehead Institute Genome Technology Core. Libraries were prepared using the KAPA HyperPrep stranded RNA kit (Roche, KK8540) following the manufacturer’s instructions. Samples were sequenced on a HiSeq2500 in High-Output mode generating 50 bases, single-end reads.

### RT-qPCR primers

RPLP0_qFGCAGCATCTACAACCCTGAAG

RPLP0_qRGCAGACAGACACTGGCAACA

FASN_qF CCGAGACACTCGTGGGCTA

FASN_qR CTTCAGCAGGACATTGATGCC

PCK1_qFGCTGGTGTCCCTCTAGTCTATG

PCK1_qRGGTATTTGCCGAAGTTGTAG.

### RNA-sequencing analysis

RNA-sequencing (RNA-seq) bioinformatics analysis was performed on the Whitehead High-Performance Computing Facility using the nf-core RNA-seq pipeline v1.4.2^112^ with Nextflow v20.04.1. Quality control of.fastq files was performed with FastQC v0.11.8. The reads were single-end, and the strandedness was set to reverse. Low-quality sequences were trimmed using Trim Galore! v0.6.4. Alignment was performed against the hg19 genome assembly using STAR v2.6.1d^116^ and duplicates were marked using Picard MarkDuplicates v2.21.1. Quantification of transcripts was performed using featureCounts v1.6.4^117^. Differential expression analysis was performed using edgeR v3.26.5^118^. deepTools v3.3.1^119^, dupRadar v1.14.0^120^, Qualimap v.2.2.2-dev^121^, and MultiQC v1.7 were used for quality control.

### Functional profiling of RNA sequencing

For functional profiling of RNA sequencing, differentially expressed genes (based on adjusted *P* value <0.05 and no log2FC cut-off) from the RNA-seq experiment were uploaded to the online version of g:Profiler^122^. Over-representation analysis was performed using g:GOSt selecting Homo sapiens (Human) as organism and treating the query set as unordered. The selected statistical domain scope was “Only annotated genes” and the significance threshold (g:SCS) was set to 0.05. Significant KEGG pathways were selected for visual representation.

### Time-correlated photoactivation localization microscopy (tc-PALM)

Widefield, live-cell, super-resolution imaging was performed in a photoactivation localization microscopy (PALM) approach using a Nikon Eclipse Ti microscope with a ×100 oil immersion objective. The 405 nm and 561 nm laser beams were combined in an external platform with customized power densities to image Dendra2-tagged molecules as previously reported^62^. Cells were cultured on imaging dishes (MatTek, P35G-1.5-20-C) and then imaged while maintaining both the temperature at 37 °C with a temperature-controlled platform and the level of CO_2_ at 5% with Leibovitz’s L-15 Medium with no phenol red (Thermo Fisher, 21083027). During each imaging cycle, a 2400-frame video stream including a (256 pixel)^2^ region of interest (ROI) was recorded in 20 Hz acquisition rate with the EM-gain setting as 1000 on an Andor iXon Ultra 897 EMCCD. Each pixel conjugates with a (160 nm)^2^ area on the sample side. After PALM imaging, the Hoechst-stained nuclei of the same ROI were imaged using a stronger 405 nm excitation through DAPI filter. For insulin stimulation, cells were first imaged in 1.5 ml insulin-free L-15 medium for 15–20 min. Afterward, the cells were stimulated with insulin by adding 1.5 ml of prewarmed and freshly made L-15 medium, 6 nM insulin to the same dish containing the original 1.5 ml of insulin-free L-15 medium. Following a 5 min wait, the cells were imaged for 15–20 min. For the insulin-unstimulated condition, cells were imaged in 1.5 ml insulin-free L-15 Medium for 15–20 min. For the insulin-treated condition, 1.5 ml fresh-made, prewarmed L-15 medium containing 2× insulin (6 nM) was directly added to the same dish while it was still on the platform, followed by a 5 min wait, then cells were imaged for 15–20 min.

### tc-PALM analysis

#### Detection localization

For each frame of a raw image, Gaussian particles were identified by pixelwise test of hypotheses, whose peak positions were individually fitted at subpixel resolution by maximum-likelihood regression with Gauss-Newton method^123^. An additional deflation loop was performed to avoid missing dimmer particles when they were overshadowed by neighboring brighter ones. This multi-particle detection localization procedure has been integrated in a published, open-source MATLAB software called MTT^123^.

#### Spatial clustering

DBSCAN and “manual selection” hybridized approach was applied to group spatially clustered detections via the qSR software^124^. Firstly, DBSCAN was performed to generalize a proposal map of spatially clustered detections. Given that IR clusters can be tiny and transient, a “loose” parameter setting was used when performing the DBSCAN (length-scale = 120 nm, N_min = 4). This parameter combination was determined by comparing the rendered, super-resolved reconstructions with the color-coded cluster maps until the clustering results visually make sense for most ROIs. Second, individual clusters were manually selected based on the clustering proposal map from the previous step. Custom MATLAB code was used to reconstruct the IR distribution of each ROI superposed with the corresponding nuclei image, which was further cross-compared with the corresponding cluster map to determine which region each cluster belongs to (i.e., plasma membrane, cytoplasm, or nucleus).

#### Temporal clustering

For each spatial cluster, time-correlated PALM (tc-PALM) analysis was performed along the time axis to extract the truly colocalized, time-correlated multi-molecule bursting events. The lifetime of a burst is simply defined as the timespan from the first to the last detections. More details about the quantitative validations and statistics of tc-PALM analysis can be found in Supplemental Text and Supplementary Fig. 13.

### Cryo-immunoelectron microscopy (EM)^125^

The cells were fixed using PLP (paraformaldehyde/lysine/sodium periodate) fixative for 4 h. Cells were pelleted and infused with a cryo-protectant for at least 1 h (PVP/ sucrose). Blocks were mounted onto cryo-pins, and snap-frozen in liquid nitrogen-cooled ethane. Ultrathin sections were cut at −140 °C with a Leica UC7 equipped with a FC7 cryo-stage using a glass knife, and immunolabeled, stained and embedded using the Tokuyasu technique. The material was examined using a Hitachi 7800.

Antibody used were anti-insulin receptor beta antibody (Cell Signaling, 23413) for WT HepG2 cells or anti-GFP (Abcam, ab6556) for HepG2 cells expressing endogenous IR tagged with GFP).

### Protein purification

Human cDNA encoding the beta subunit of the insulin receptor (IRb; residues 763–1382) was cloned into a mammalian expression vector. The base vector was engineered to include sequences encoding an N-terminal FLAG tag followed by mCherry and a 14 amino acid linker sequence “GAPGSAGSAAGGSG.” cDNA sequences were inserted in-frame following the linker sequence using NEBuilder HiFi DNA Assembly Master Mix (NEB, E2621S). The expression construct was subjected to Sanger sequencing to confirm the sequence.

For protein expression, IRb-mcherry plasmid was transfected into HEK293T cells (ATCC, CRL-3216) using Polyethylenimine (Fisher Scientific, NC1014320). Cells were cultured for 72 h, scraped off the plate, and washed with ice-cold PBS (Life Technologies, AM9625). Cells were centrifuged at 500×*g* for 5 min, and the cell pellet was stored at −80 °C.

The cell pellet was resuspended in 35 ml Lysis Buffer (20 mM HEPES pH7.4, 150 mM NaCl, 1 mM EDTA, 0.5% NP40, with fresh inhibitors and 1 mM DTT). Cell lysate was rocked for 30 min at 4 °C and spun down at 12,000×*g* 15 min. In all, 35 ml of supernatant was removed to a fresh tube and centrifuged again if cloudy. In total, 300 μl of washed Anti-Flag M2 magnetic beads (Sigma Aldrich, M8823) was added to the lysate, which was then rotated overnight at 4 °C. The next day beads were pelleted at 500 rpm for 5 min, washed with 35 ml BD Buffer (10 mM HEPES, 450 mM NaCl, 5% glycerol with fresh inhibitors), and transferred to an Eppendorf tube. Tubes were then placed in a magnetic rack to pellet beads and washed 3–5 times with BD Buffer, with resuspension of the pellet for each wash. Elution was performed overnight with 500 μl Dialysis Buffer (50 mM HEPES, 150 mM NaCl, 5 mM MgCl_2_, 5% glycerol) plus 50 μl Flag peptide (5 mg/ml stock solution). The next day, the sample was eluted with the magnetic rack and washed with 250 μl Dialysis buffer with no peptide. The sample was dialyzed with 500 ml buffer, which was changed 1 to 2 times at 4 °C.

### Insulin-binding assay

Insulin-sensitive and insulin-resistant cells were washed in EMEM for 30 min as previously described, incubated on ice at 4 °C for 30 min, and treated with 3 nM insulin for 60 min on ice at 4 °C with gentle agitation. Cells were washed five times with ice-cold PBS. TrypLE was added to the wells, and cells were gently detached from the wells and added to a 1.5-ml tube. Cells were incubated at 37 °C for 10 min with gentle shaking. Cells were then pelleted, and the supernatant was collected into the new 1.5-ml tube and incubated O/N at 37 °C with gentle shaking. Samples were then used for proteomics.

### Proteomics

#### SDB-RPS extraction of tryptic peptides from cell cultures

For the preparation of tryptic peptides for mass spectrometry, 50 μl protein extract was reduced by adding 2 μl 250 mM TCEP and the solution was incubated for 15 min at 55 °C. Then 5 μl 500 mM IAA was added, and the proteins were alkylated for 30 min at RT. The extracts were acidified (0.5% w/v TFA) and extracted by using custom-made SDB-RPS tips (CDS Analytical, Oxford, PA, USA) following the descriptions by Rappsilber et al.^126^. Peptides were eluted from SBD-RPS filters with 80% (v/v) ACN and 5% (v/v) ammonium hydroxide, dried in a lyophilizer, and taken up in 20 µL 0.2% (v/v) formic acid. Insoluble material was removed from reconstituted peptide solutions by centrifugation for 10 min at 20,000×*g* at 4 °C prior to analysis with mass spectrometry.

#### nanoLC-MS and data analysis

The LC-MS/MS analysis was performed using an Easy-nLC 1200 system connected to an Orbitrap Exploris 480 mass spectrometer (Thermo Fisher Scientific, Waltham, MA, USA) equipped with an Easy Spray ESI source together with FAIMS for the ionization of eluting fractions. Peptide separation, collection of MS1 and MS2 profiles, identification and quantitation of protein abundances, and statistical data analyses were carried out as described by Schulte et al.^127^.

### Statistics and reproducibility

Statistical analysis was performed using Prism Version 9.4.0 (GraphPad, La Jolla, CA) and Excel Version 16.66.1. The statistical test used is reported in the figure legend. Data are represented as individual values and mean ± SEM or as mean ± SEM. The exact *P* value is reported in the Source Data file (given the limitation of Excel, very low *P* values are reported as 0). Information on the number of independent experiments performed is reported in Supplementary Table 2. All imaging experiments have been repeated independently at least two times as reported in Supplementary Table 2. All tc-PALM experiments were performed three times using biologically independent replicates, and the results were merged. Results reported in Fig. 1 derive from three independent experiments. All imaging of IR (immunofluorescence and live-cell imaging) in HepG2 were performed three times using biologically independent samples, unless otherwise stated in Supplementary Table 2. All imaging of pIRS1 in IR clusters have been performed twice using biologically independent samples. Imaging of NRF2 and ROS have been performed twice using biologically independent samples unless otherwise stated in Supplementary Table 2. Imaging of IR in human primary hepatocytes was performed once with biologically independent samples. Imaging of IR in human primary adipocytes was performed twice with biologically independent samples. Western blot, ELISA and RT-qPCR experiments have been performed ≥2 times using biologically independent samples. RNA-seq, metabolomics experiments, and proteomics experiments were performed once with biologically independent samples. Quantification of the number of IR molecules was performed twice. Survival assay was performed three times using biologically independent samples.

### Schematics

BioRender was used to make the graphics reported in the figures (BioRender.com).

## Supplementary information

Supplementary information

Peer Review File

Supplementary Table 2

## Data Availability

ChIP-sequencing and RNA-sequencing data generated in this study have been deposited in GEO: GSE181096. Source data are provided with this paper. Proteomics data have been deposited in MassIVE database: ID MSV000090743. Metabolomics data have been submitted to Metabolights, ID MTBLS6505.

## Source data

Source Data

## Code availability

All codes are available to researchers by the corresponding authors.
