# Supplementary material for: The dynamic clustering of insulin receptor underlies its signaling and is disrupted in insulin resistance
Source: Nat Commun. 2022 Dec 6;13:7522. doi: 10.1038/s41467-022-35176-7 (PMC9727033; doi:10.1038/s41467-022-35176-7)
Supplement: Supplementary file 1 — Supplementary information [file 41467_2022_35176_MOESM1_ESM.pdf]

Supplementary Material for:

The dynamic clustering of insulin receptor underlies its signaling and is disrupted in insulin resistance

Alessandra Dall'Agnese#, Jesse M. Platt#, Ming M. Zheng, Max Friesen, Giuseppe Dall'Agnese, Alyssa M. Blaise, Jessica B. Spinelli, Jonathan E. Henninger, Erin N. Tevonian, Nancy M. Hannett, Charalampos Lazaris, Hannah K Drescher, Lea M Bartsch, Henry R. Kilgore, Rudolf Jaenisch, Linda G. Griffith, Ibrahim I. Cisse, Jacob F. Jeppesen, Tong Ihn Lee*, Richard A. Young*

#these authors contributed equally

*Correspondance to young@wi.mit.edu and tlee@wi.mit.edu

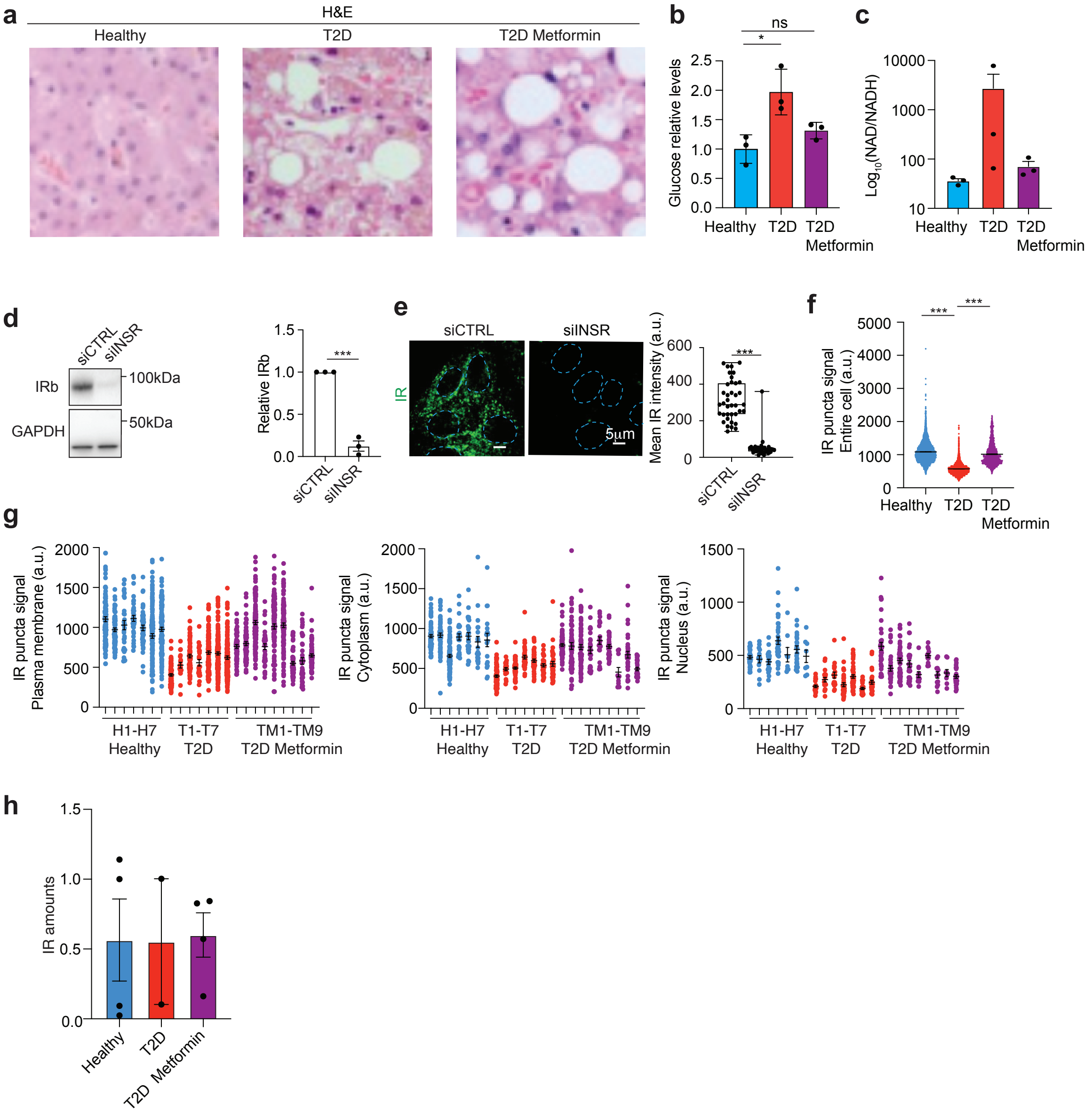

Supplementary Fig. 1. Human liver characterization and antibody validation. a, Representative hematoxylin and eosin (H&E) images of human livers from a healthy donor (Healthy), a donor with T2D (T2D) and a donor with T2D who had been treated with metformin (T2D Metformin). **b**, Quantification of relative glucose levels in livers from healthy donors (Healthy, light blue), donors with T2D (T2D, red) and donors with T2D who had been treated with metformin (T2D Metformin, purple) as determined by metabolomics. Data is represented as individual values and as mean +/- SEM. Liver samples from 3 donors were analyzed per condition. Unpaired two-sided t-test was used for statistical analysis. **c**, Quantification of NAD/NADH ratio in livers from healthy donors (Healthy, light blue), donors with T2D (T2D, red) and donors with T2D who had been

treated with metformin (T2D Metformin, purple) as determined by metabolomics. Data is represented as individual values and as mean +/- SEM. Liver samples from 3 donors were analyzed per condition. **d,e,** Validation of the antibody against IR by immunoblot (d) and immunofluorescence (e) and quantification. Data is represented as individual values and as mean +/- SEM. For immunoblot validation, 3 biological replicates were analyzed per condition. For immunofluorescence validation, 38 siCTRL cells and 36 siINSR cells were analyzed. Unpaired two-sided t-test was used for statistical analysis. siCTRL minima 142, maxima 518, centre 288, 25th percentile 235, 75th percentile 404; siINSR minima 13, maxima 360, centre 41, 25th percentile 31; 75th percentile 51. **f**, Automated quantification of IR signal in puncta in entire cells (without specifying cellular subcompartments) of healthy donors (Healthy, light blue), donors with T2D (T2D, red) and donors with T2D who had been treated with metformin (T2D Metformin, purple). Data is represented as mean +/- SEM. Number of IR puncta analyzed: Healthy 5891 puncta, T2D 3118 puncta, T2D Metformin 1271. Unpaired two-sided t-test was used for statistical analysis. **g**, Quantification of IR signal in puncta at the plasma membrane, cytoplasm and nucleus in healthy donors (H1-H7, Healthy, light blue), donors with T2D (T1-T7, T2D, red) and donors with T2D who had been treated with metformin (TM1-TM9, T2D Metformin, purple). Quantification for each individual donor is shown. Data is represented as mean +/- SEM. Number of puncta analyzed: Plasma membrane, going from left (sample H1) to right (sample TM9) along the x-axis = 97, 69, 31, 36, 45, 147, 135, 70, 19, 74, 24, 135, 161,135, 54, 77, 138, 37, 120, 118, 57, 41, 74; Cytoplasm, going from left (sample H1) to right (sample TM9) along the x-axis = 68, 55, 91, 26, 25, 21, 18, 69, 20, 52, 37, 38, 30, 37, 67, 57, 68, 28, 22, 36, 10, 38, 24; Nucleus, going from left (sample H1) to right (sample TM9) along the x-axis = 26, 13, 19, 36, 8, 27, 8, 21, 19, 17, 36, 44, 21, 29, 38, 31, 44, 27, 12, 13, 12, 9, 25. **h**, Quantification of relative IR levels by immunoblot. IR level was normalized to CK18 and represented relative to sample Healthy 3 (H3). Data is represented as mean +/- SEM. Number of liver samples analyzed: Healthy 4 liver samples, T2D 2 liver samples, T2D Metformin 4 liver samples. Source data are provided as a Source Data file.

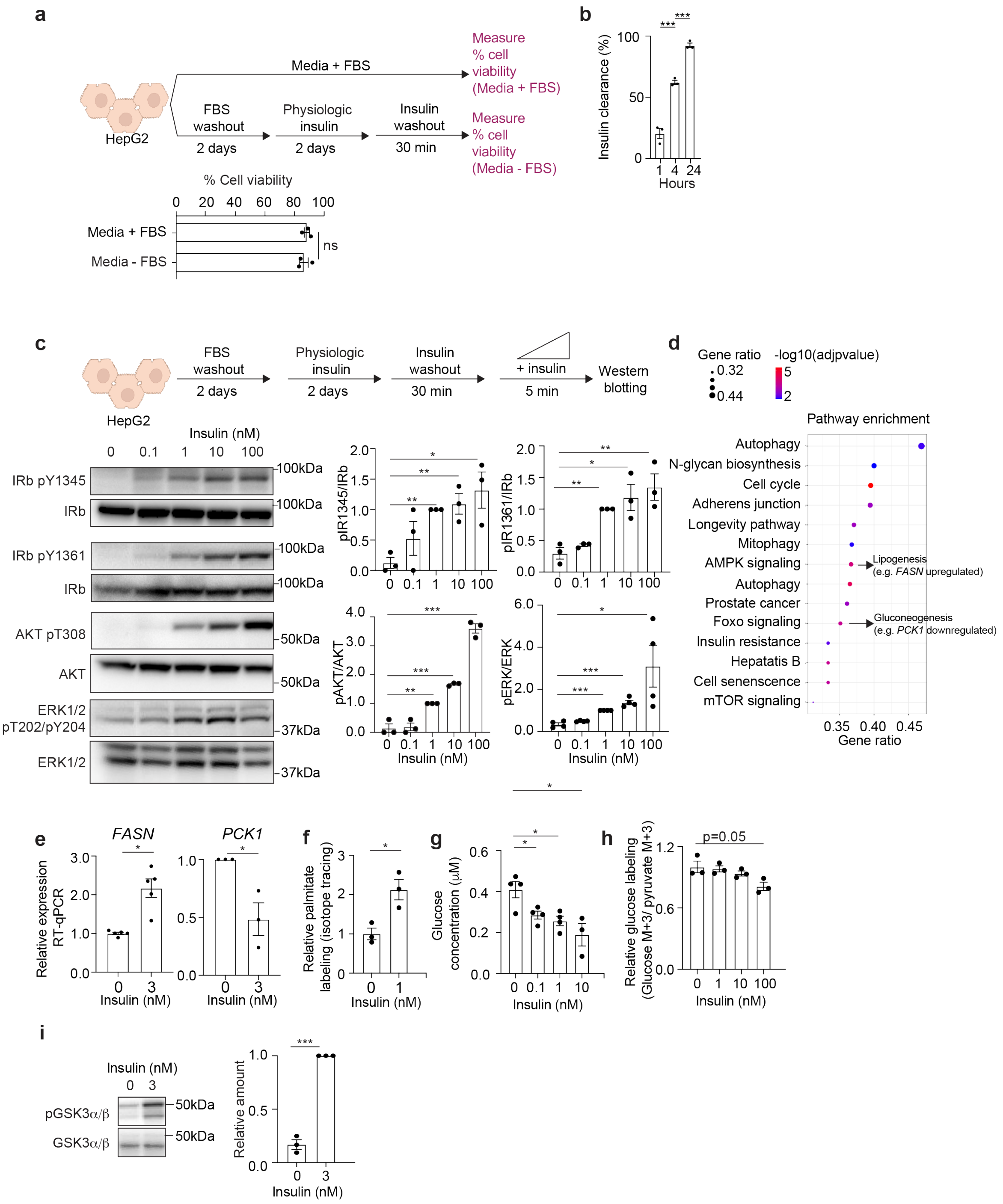

Supplementary Fig. 2. Validation of insulin-sensitive HepG2 cell model. a, Schematic of cell treatment (top). Percent viability of cells cultured in cell expansion media (Media + FBS, n=3 biological replicates) or in media containing physiological concentrations of insulin (Media – FBS, n=3 biological replicates) is reported in the graph (bottom). Data is reported as mean +/- SEM. Unpaired two-sided t-test was used for statistical analysis. **b**, Quantification of insulin clearance at 1, 5 or 24 hours in insulin-

sensitive cells treated with 3nM insulin (1 hours, n=3 biological replicates; 5 hours, n=3 biological replicates, 24 hours n=3 biological replicates). Data is reported as mean +/- SEM. Unpaired two-sided t-test was used for statistical analysis. **c**, Experimental protocol (top) and immunoblot with quantitation (bottom) to measure phosphorylated insulin signaling proteins (pIRb, pAKT, pERK) over total insulin signaling proteins (IRb, AKT, ERK). 3 biological replicates per condition were analyzed for pIR/IR and for pAKT/AKT, 4 biological replicates were analyzed for pERK/ERK. Data is represented as mean +/- SEM. Unpaired two-sided t-test was used for statistical analysis. **d**, Gene ontology of the differentially expressed genes after 4 hours of 3nM insulin stimulation (3 biological replicates). The y-axis corresponds to the KEGG pathways. The x-axis and the point size represent the "Gene Ratio" defined as the fraction of differentially expressed genes in each given ontology term (in this case KEGG pathway). The color corresponds to -$\log_{10}$(adjusted p-value). **e**, Relative expression of *FASN* (n=5 biological replicates) and *PCK1* (n=3 biological replicates) in HepG2 cells acutely stimulated with (3) or without (0) insulin for 4 hours. Data is represented as mean +/- SEM. Two-sided unpaired t-test was used for statistical analysis. **f**, Isotope tracing experiment showing relative palmitate labeling in HepG2 cells acutely stimulated with 0nM or 1nM insulin for 36 hours. Data is represented as mean +/- SEM. 3 biological replicates were analyzed per condition and two-sided unpaired t-test was used for statistical analysis. **g**, Quantification of glucose production in cells stimulated with 0, 0.1, 1 or 10nM insulin for 5 hours. Data is represented as mean +/- SEM. 4 biologically independent samples were analyzed for conditions 0nM insulin, 0.1nM insulin and 1nM insulin, while 3 biologically independent samples were analyzed for condition 10nM insulin. Two-sided unpaired t-test was used for statistical analysis. **h**, Isotope tracing experiment showing relative glucose labeling in HepG2 cells acutely stimulated with 0, 1, 10 or 100nM insulin for 24 hours. Data is represented as mean +/- SEM. 3 biological replicates were analyzed per condition and unpaired two-sided t-test was used for statistical analysis. **i**, Immunoblot to quantify phosphorylated GSKα/β (pGSKα/β) over total GSKα/β protein in HepG2 cells acutely stimulated with 0nM or 3nM insulin for 5 minutes. 3 biological replicates were analyzed per condition, data is represented as mean +/- SEM and unpaired two-sided t-test was used for statistical analysis. This is the same experiment as in Supplementary Fig. 6j. Source data are provided as a Source Data file.

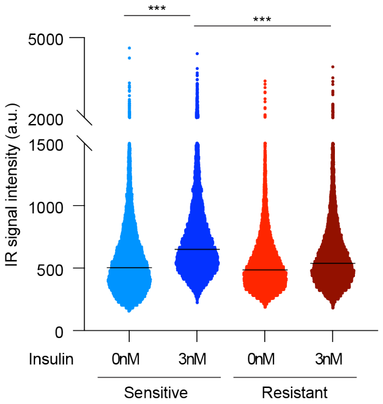

Supplementary Fig. 3. Automated quantification of IR signal intensity in puncta. a, Quantification of IR signal intensity in puncta in entire cells (without specifying cellular subcompartments), relative to Fig. 2b. Data is represented as individual values and mean +/- SEM. Number of IR puncta analyzed: Sensitive 0nM insulin (light blue) 16,984 puncta, Sensitive 3nM insulin (blue) 16,397 puncta, Resistant 0nM insulin (red) 15,296 puncta, Resistant 3nM insulin (dark red) 14,708 puncta. Unpaired two-sided t-test was used for statistical analysis. Source data are provided as a Source Data file.

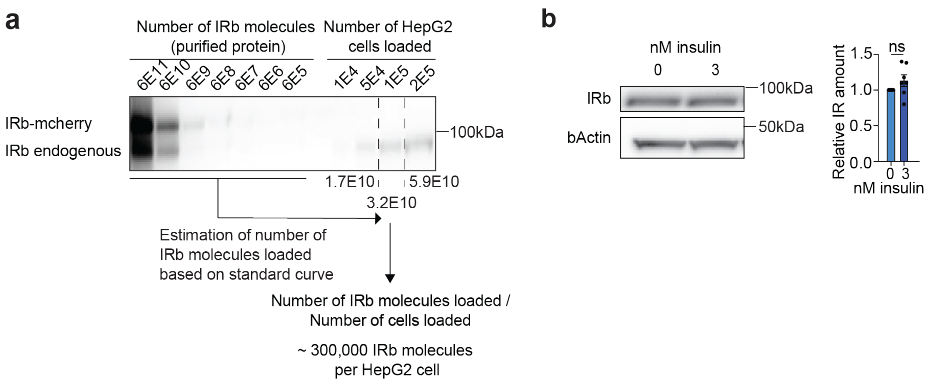

Supplementary Fig. 4. Quantification of the number of IR molecules in HepG2 cells. **a,** Quantitative western blot with standard curve of purified IRbeta mCherry fusion protein (IRb-mCherry; first 7 lanes) and cell lysate containing a specific number of cells (last four lanes). **b,** Immunoblot for IRbeta (IRb) and beta-actin (bActin) in cells treated acutely with 0nM or 3nM insulin (left). Quantification of relative IRb levels in HepG2 cells without (0nM, light blue) and with (3nM, dark blue) acute insulin stimulation (right). IRb level was normalized to beta-actin. 7 biological replicates were analyzed per condition. Data is represented as individual values and as mean +/- SEM. Unpaired two-sided t-test was used for statistical analysis. Source data are provided as a Source Data file.

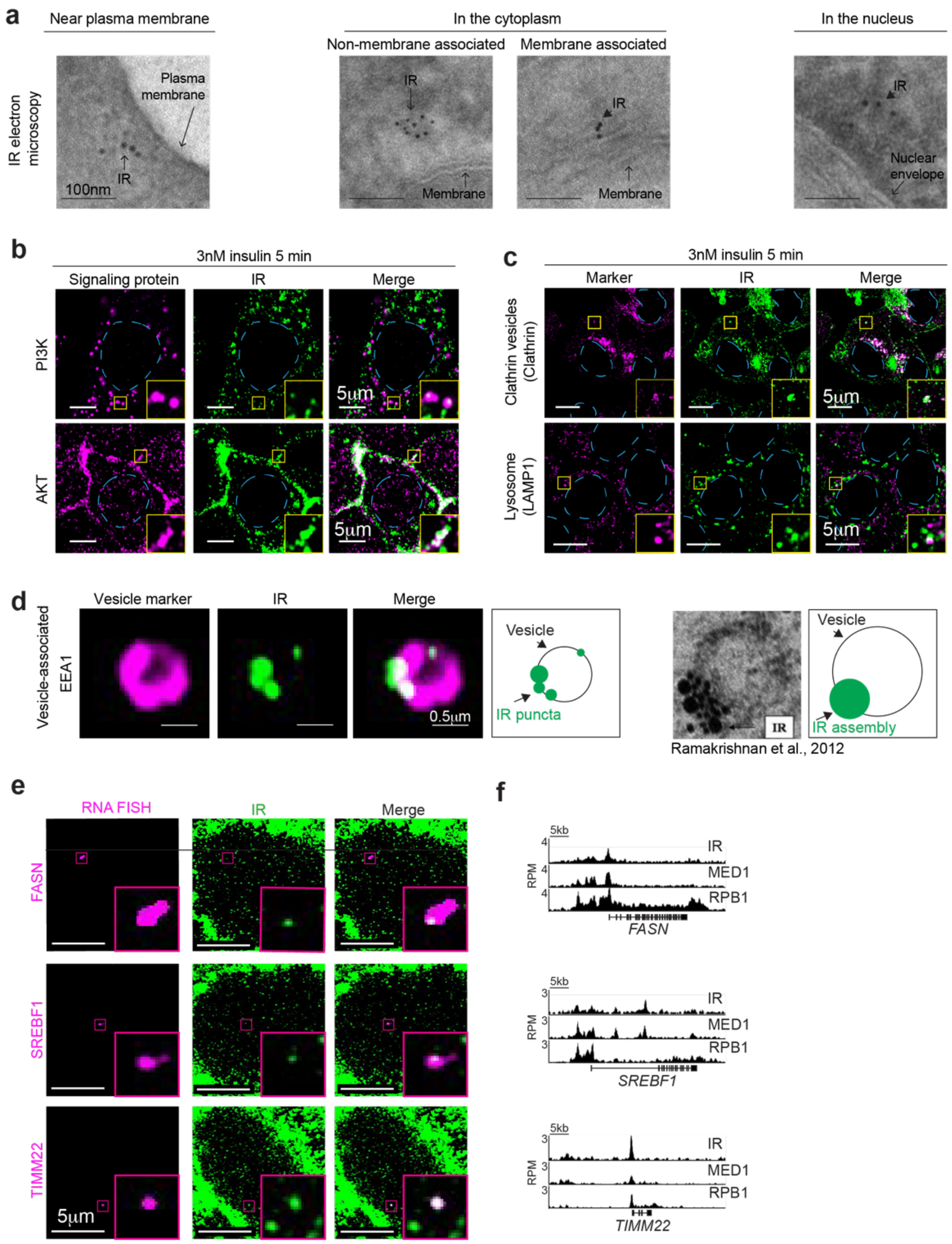

Supplementary Fig. 5. IR puncta in various cellular compartments in insulin-sensitive cells. a, Representative electron microscopy images for IR showing its presence near the plasma membrane, in the cytoplasm and in the nucleus. **b**, Representative immunofluorescence images for PI3K or AKT (magenta) together with IR (green) in insulin-sensitive HepG2 cells acutely stimulated with insulin for 5 minutes. Dashed light blue lines represent nuclear outline. Representative colocalization area (yellow box) is magnified at the bottom right corner of each image. **c**, Representative

immunofluorescence images for clathrin or LAMP1 together with IR (green) in insulin-sensitive HepG2 cells acutely stimulated with insulin for 5 minutes. IR was detected either by immunofluorescence or by imaging endogenous IR-GFP. Dashed light blue lines represent nuclear outline. Representative colocalization area (yellow box) is magnified at the bottom right corner of each image. **d**, Representative immunofluorescence images for EEA1 (endosome marker) and IR, with a schematic representation of IR puncta associated with a portion of the vesicle membrane (left). Published electron microscopy image of IR and another receptor associated with a portion of the membrane of a vesicle[1], with a schematic representation of IR puncta associated with the vesicle (right). Reuse of the published image[1] is granted under STM guidelines. **e,** Colocalization of IR and nascent RNA of *FASN, SREBF1* and *TIMM22* determined by imaging IR-GFP and *FASN, SREBF1* and *TIMM22* intronic RNA FISH in cells stimulated with 3nM insulin. Colocalization area (magenta box) is magnified at the bottom right corner of each image. Scale bars are indicated in the images. *FASN, SREBF1* and *TIMM22* are known insulin-responsive genes[2, 3, 4, 5, 6, 7]. If the fluorescence made the scale bar hard to see, a black box was added behind the scale bar. **f**, ChIP-seq tracks of IR, MED1 and RPB1 at *FASN, SREBF1* and *TIMM22* loci.

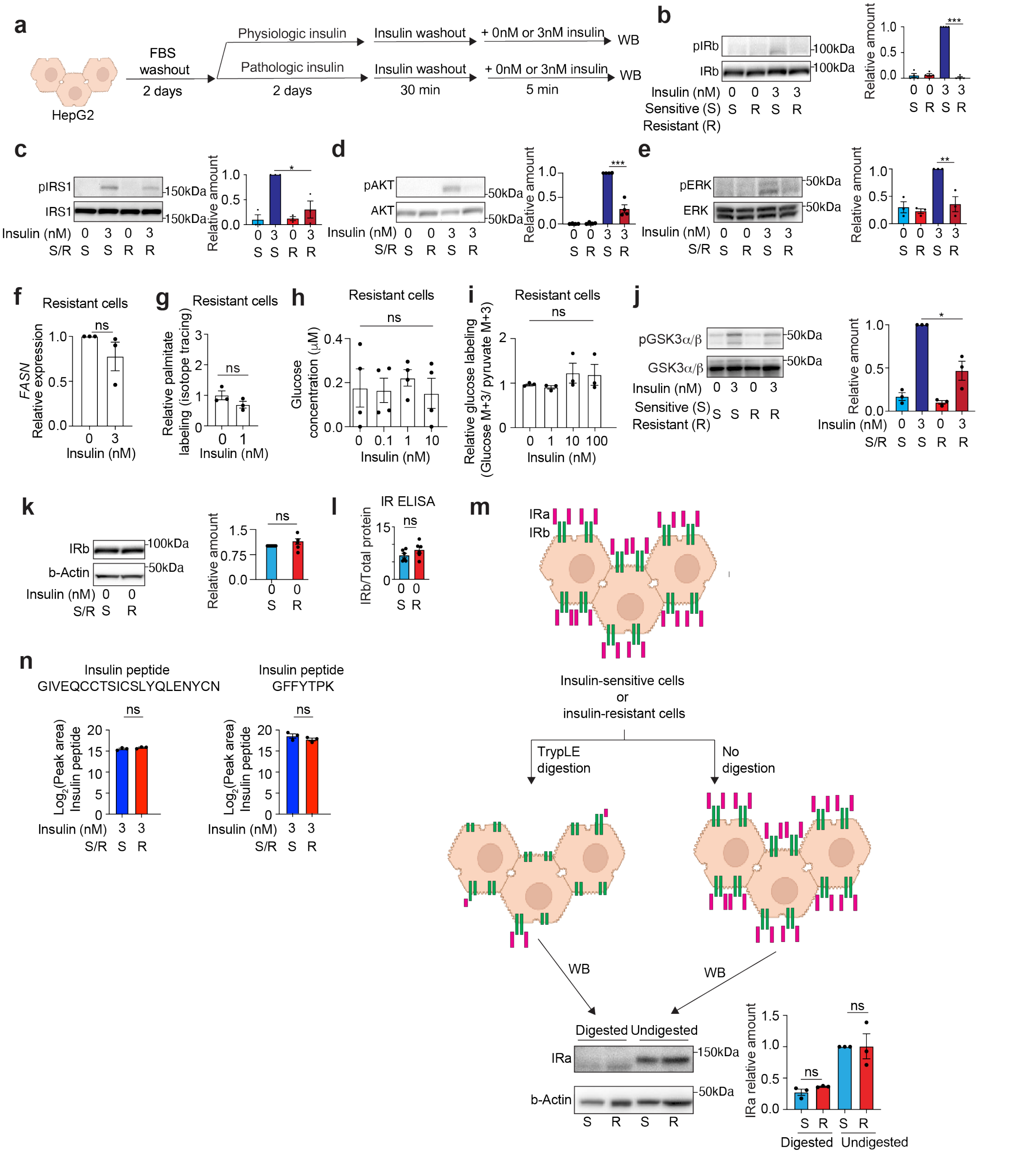

Supplementary Fig. 6. Validation of insulin-resistant HepG2 cell model. a, Schematic of cell treatments. **b-e**, Immunoblot with quantitation to measure phosphorylated insulin signaling proteins (pIRb, pIRS1, pAKT, pERK) over total insulin signaling proteins (IRb, IRS1, AKT, ERK) in insulin-sensitive (Sensitive, S, light blue) and insulin-resistant (Resistant, R, red) cells stimulated with 0nM (light color) or 3nM (dark color) insulin for 5 minutes. For figures b and d four biological replicates were analyzed, for figures c and e

three biological replicates were analyzed. Individual replicates are shown in the graphs and bar graphs represent mean +/- SEM. Unpaired two-sided t-test was used for statistical analysis. **f**, Relative expression of *FASN* in insulin-resistant HepG2 cells acutely stimulated with 0nM or 3nM insulin for 4 hours. 3 biological replicates were analyzed. Individual data points are represented as well as the mean +/- SEM. Unpaired two-sided t-test was used for statistical analysis. **g**, Isotope tracing experiment showing relative palmitate labeling in insulin-resistant HepG2 cells acutely stimulated with 0nM or 1nM insulin for 36 hours. 3 biological replicates were analyzed. Individual values are reported, bar graph represents mean +/- SEM. Unpaired two-sided t-test was used for statistical analysis. **h**, Quantification of glucose production in insulin-resistant HepG2 cells stimulated with 0, 0.1, 1 or 10nM insulin for 5 hours. 4 biological replicates were analyzed. Individual values are reported, bar graph represents mean +/- SEM. Unpaired two-sided t-test was used for statistical analysis. **i**, Isotope tracing experiment showing relative glucose labeling in insulin-resistant HepG2 cells acutely stimulated with 0, 1, 10 or 100nM insulin for 24 hours. 3 biological replicates were analyzed. Individual values are reported, bar graph represents mean +/- SEM. Unpaired two-sided t-test was used for statistical analysis. **j**, Immunoblot to quantify phosphorylated GSKα/β (pGSKα/β) over total GSKα/β protein in insulin-sensitive (Sensitive, S, blue) and insulin-resistant (Resistant, R, red) HepG2 cells acutely stimulated with 0nM (light color) or 3nM (dark color) insulin for 5 minutes. 3 biological replicates were analyzed. Individual values are reported, bar graph represents mean +/- SEM. Unpaired two-sided t-test was used for statistical analysis. **k**, Immunoblot for IRbeta (IRb) and beta-actin (b-Actin) in insulin-sensitive (light blue) and insulin-resistant (red) cells unstimulated with insulin (left). Quantification of relative IRb levels (right). 5 biological replicates were analyzed. Individual values are reported, bar graph represents mean +/- SEM. Unpaired two-sided t-test was used for statistical analysis. **l**, Enzyme-linked immunoassay (ELISA) for IRbeta (IRb) relative to total protein in insulin-sensitive (S, light blue) and insulin-resistant (R, red) cells unstimulated with insulin. 6 biological replicates were analyzed. Individual values are reported, bar graph represents mean +/- SEM. Unpaired two-sided t-test was used for statistical analysis. **m**, Schematic of proteolytic shaving experiment. Insulin-sensitive or resistant cells were either treated with TrypLE to digest the portions of proteins at the cell surface (Digested) or not (Undigested). Immunoblot with quantitation to measure IRalpha (IRa) and beta-

actin (b-Actin) in digested and undigested insulin-sensitive (S, light blue) and insulin-resistant (R, red) cells. 3 biological replicates were analyzed. Individual values are reported, bar graph represents mean +/- SEM. Unpaired two-sided t-test was used for statistical analysis. **n**, Proteomic quantification of insulin binding in insulin-sensitive (S, blue) and insulin-resistant (R, red) cells treated with 3nM insulin at 4°C. Peak area quantification is reported for two insulin peptides: GIVEQCCTSICSLYQLENYCN (insulin A-chain) and GFFYTPK (insulin B-chain). 3 biological replicates were analyzed. Individual values are reported, bar graph represents mean +/- SEM. Unpaired two-sided t-test was used for statistical analysis. Source data are provided as a Source Data file.

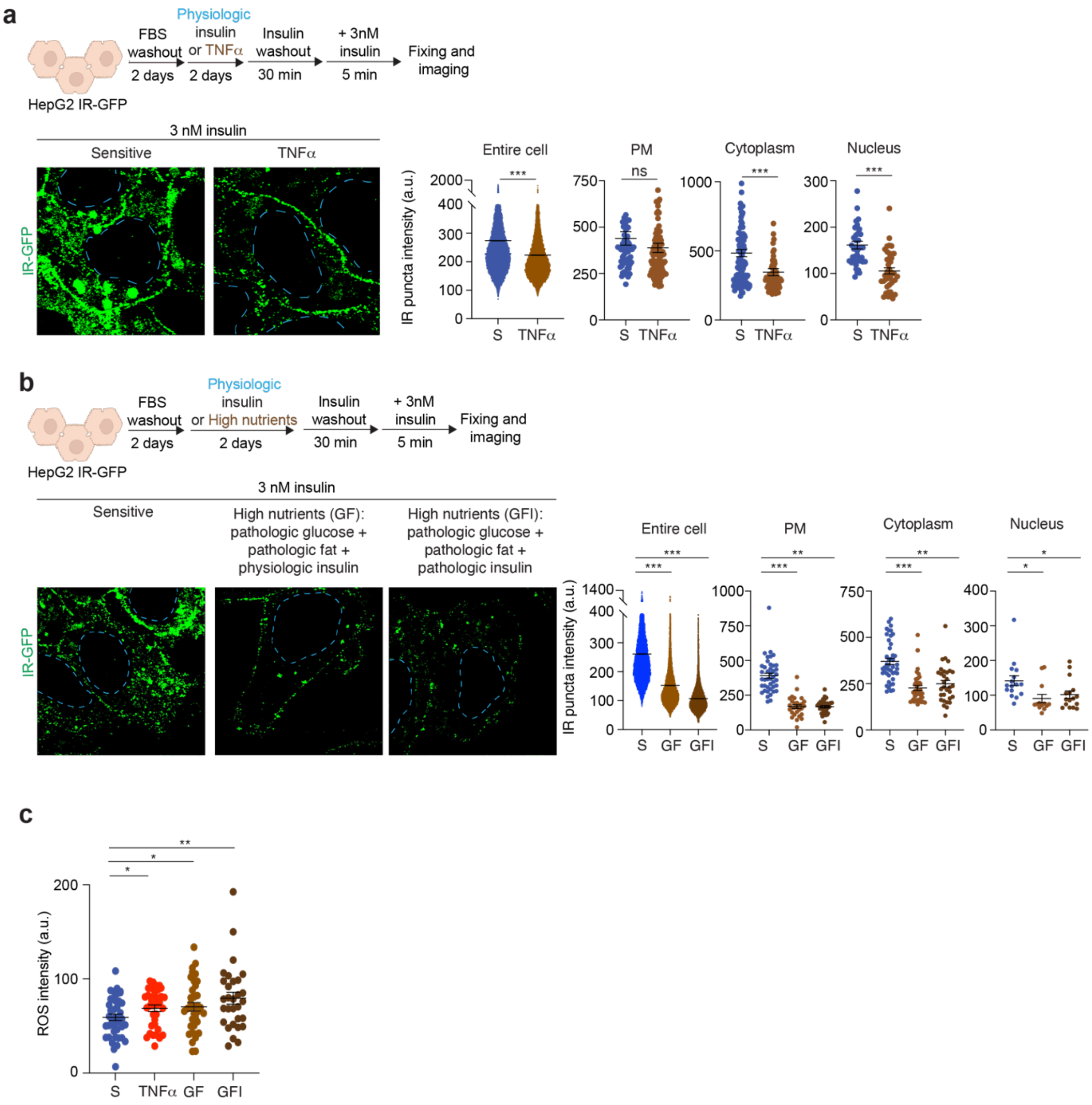

Supplementary Fig. 7. Other models of insulin resistance. a, Schematic of cell treatments (top). Imaging of IR-GFP in HepG2 cells treated with physiological concentrations of insulin (Sensitive, S, blue) or pathological concentration of TNFα (TNFα, brown) and acutely stimulated with 3nM insulin for 5 minutes (bottom left). Quantification of IR signal intensity in IR puncta in the entire cell (automated quantification, without specifying cellular subcompartments), at the plasma membrane (PM), cytoplasm or nucleus of cells (bottom right). In the graph individual values and the mean +/- SEM are reported. Number of IR puncta analyzed: Entire cell Sensitive 29,398 puncta, TNFα 31,083 puncta; Plasma membrane Sensitive 66 puncta, TNFα 112 puncta;

Cytoplasm Sensitive 109 puncta, TNFα 76 puncta; Nucleus Sensitive 40 puncta, TNFα 40 puncta. Unpaired two-sided t-test was used for statistical analysis. **b**, Schematic of cell treatments (top). Imaging of IR-GFP in HepG2 cells treated with physiological concentrations of insulin (Sensitive, S, blue) or with high nutrients (either 1) pathological concentrations of glucose and fat and physiological concentration of insulin (high nutrients, GF, brown) or 2) pathological concentrations of glucose, fat and insulin (high nutrients, GFI, dark brown) and acutely stimulated with 3nM insulin for 5 minutes (bottom left). Quantification of IR signal intensity in IR puncta in the entire cell (automated quantification, without specifying cellular subcompartments), at the plasma membrane (PM), cytoplasm or nucleus of cells (bottom right). In the graph, individual values and the mean +/- SEM are reported. Number of IR puncta analyzed: Entire cell Sensitive 21,382 puncta, GF 17,715 puncta, GFI 20,514 puncta; Plasma membrane Sensitive 42 puncta, GF 29 puncta, GFI 32 puncta; Cytoplasm Sensitive 44 puncta, GF 41 puncta, GFI 31 puncta; Nucleus Sensitive 16 puncta, GF 13 puncta, GFI 16 puncta. Unpaired two-sided t-test was used for statistical analysis. **c**, ROS intensity in insulin-sensitive HepG2 cells (blue), in cells treated with TNFα (red), or in cells treated with high nutrients (either 1) pathological concentrations of glucose and fat and physiological concentration of insulin (GF, brown) or 2) pathological concentrations of glucose, fat and insulin (GFI, dark brown). Physiological concentration of insulin corresponds to 0.1nM, pathological concentration of insulin corresponds to 3nM, pathological concentration of TNFα corresponds to 100pg/ml, pathological concentration of fat corresponds to 30μM palmitic acid and 45μM oleic acid, pathological concentration of glucose corresponds to 10mM. In the graph, individual values and the mean +/- SEM are reported. Number of cells analyzed: Sensitive 42 cells, TNFα 35 cells, GF 36 cells, GFI 30 cells. Unpaired two-sided t-test was used for statistical analysis. Source data are provided as a Source Data file.

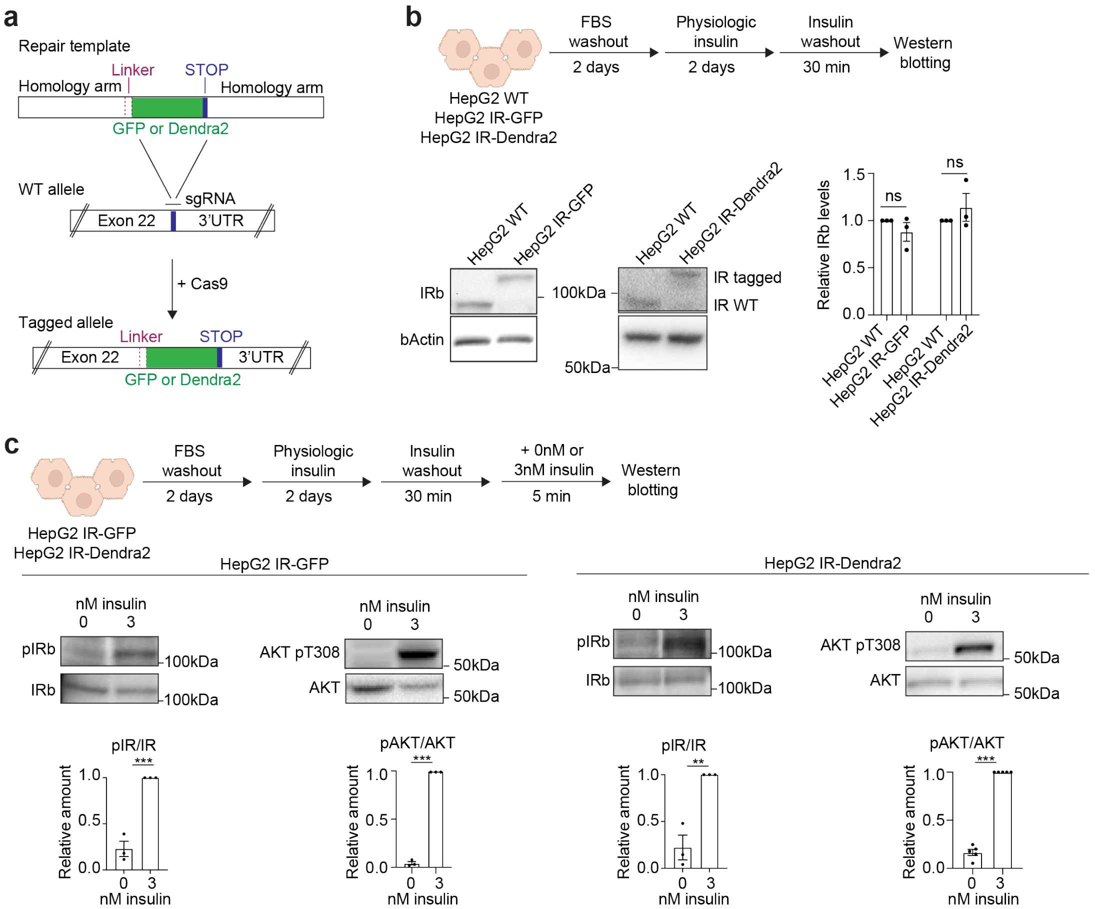

Supplementary Fig. 8. Homozygous HepG2 cell lines expressing functional endogenous IR tagged with GFP or Dendra2. a, Schematic of knock-in strategy. **b**, Schematic of cell treatments (top). Immunoblot for IRbeta (IRb) and beta-actin (bActin) control in WT, IR-GFP and IR-Dendra2 cell lines (bottom left). The shift in molecular weight is the expected size for the GFP or Dendra2 fusion with IR. Quantitation of IRb levels (bottom right). Individual values are reported and the bar graphs represent mean +/- SEM. 3 biological replicates were analyzed and unpaired two-sided t-test was used for statistical analysis. **c,** Schematic of cell treatments (top). Immunoblot with quantitation to measure phosphorylated insulin signaling proteins (pIRb and pAKT) over total insulin signaling proteins (IRb and AKT) in IR-GFP and IR-Dendra2 cells stimulated with 0nM or 3nM insulin for 5 minutes (bottom). Individual values are reported and the bar graphs represent mean +/- SEM. 3 biologically independent replicates were analyzed for pIRb/IRb in HepG2 IR-GFP cells and HepG2 IR-Dendra2 cells. 3 biologically independent replicates were analyzed for pAKT/AKT in IR-GFP cells and 4 biologically independent

replicates were analyzed for pAKT/AKT in IR-Dendra2 cells. Unpaired two-sided t-test was used for statistical analysis. Source data are provided as a Source Data file.

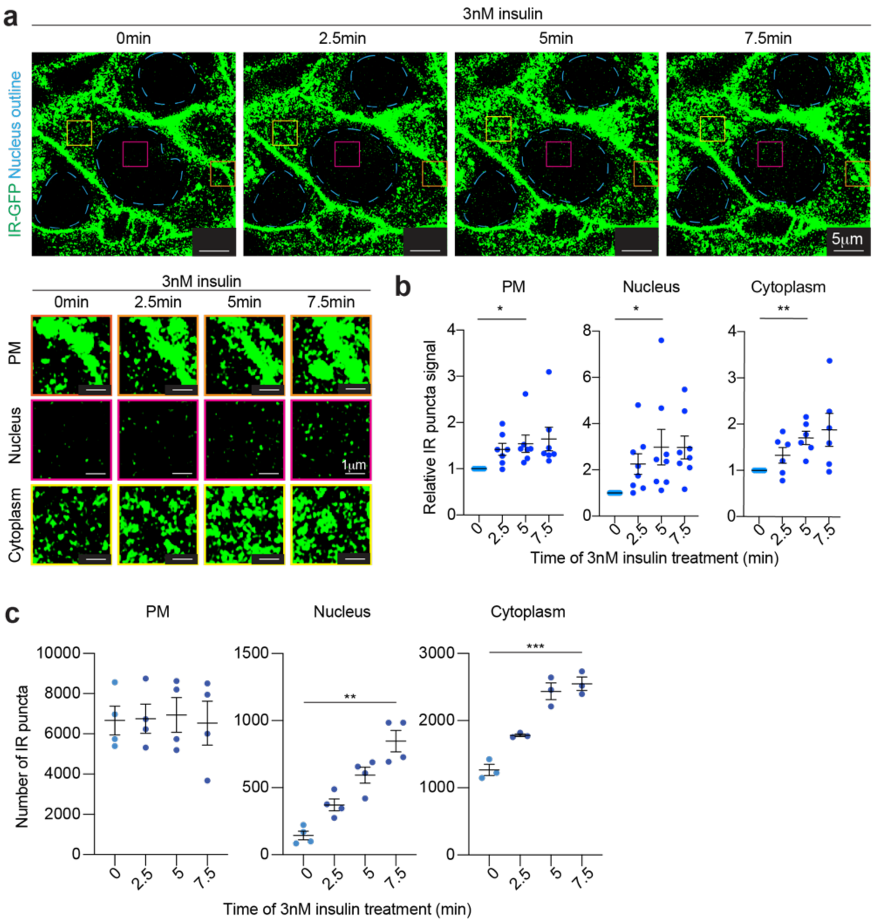

Supplementary Fig. 9. Live-cell imaging of IR puncta in HepG2 cells. a, Live imaging time course of HepG2 cells expressing endogenous IR tagged with GFP during insulin stimulation. Time of acquisition is reported above images. Dashed light blue lines represent nuclear outline and scale bar are indicated in the images. Representative images of three cells (top). Orange, magenta and yellow boxes represent regions at the plasma membrane (PM), nucleus and cytoplasm, respectively, that are magnified at the bottom. If the fluorescence made the scale bar hard to see, a black box was added behind the scale bar. **b,** Quantification of IR puncta signal at the plasma membrane (PM), nucleus and cytoplasm of IR-GFP cells stimulated with 3nM insulin for 0, 2.5, 5 and 7.5 minutes. Data is represented as “relative to 0 minutes”. In the graphs, individual values and the mean +/- SEM are reported. Number of regions analyzed: Plasma membrane 7 regions, Cytoplasm 6 regions, Nucleus 8 regions. Unpaired two-sided t-test was used for statistical analysis. **c**, Quantification of number of IR puncta at the plasma membrane (PM), nucleus and cytoplasm of IR-GFP cells stimulated with 3nM insulin for 0, 2.5, 5 and 7.5 minutes. In the graphs, individual values and the mean +/- SEM are reported. Number of cells

analyzed: Plasma membrane 4 cells, Cytoplasm 3 cells, Nucleus 4 cells. Unpaired two-sided t-test was used for statistical analysis. Source data are provided as a Source Data file.

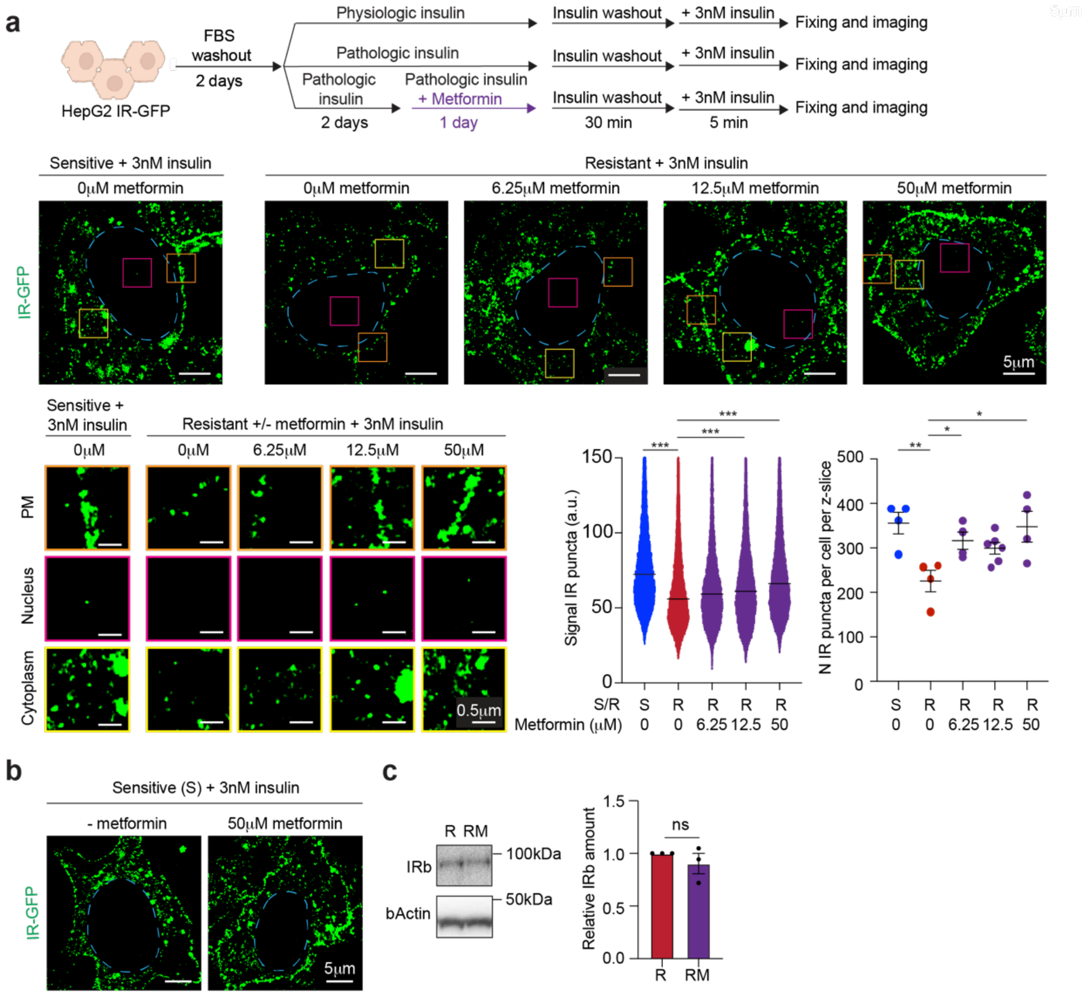

Supplementary Fig. 10. Metformin effect on IR puncta. a, Schematic of cell treatments (top). Imaging of IR-GFP in insulin-sensitive and insulin-resistant cells treated with or without metformin (middle). Metformin concentration is reported above the images. IR-GFP fluorescence signal is shown in green. Dashed light blue lines represent nuclear outline. Orange, magenta and yellow boxes represent regions at the plasma membrane (PM), nucleus and cytoplasm, respectively, that are magnified (bottom left). Scale bars are indicated in the images. This is the same experiment as in Fig. 2d and thus the same images for insulin-sensitive cells, insulin-resistant cells and insulin-resistant cells treated with 12.5μM metformin are reported in Fig. 2d. Quantification of IR signal in puncta (automated quantification) and the number of IR puncta in insulin-sensitive (blue) or insulin-resistant cells treated with (purple) or without metformin (red) (bottom right). In the graphs, individual values and the mean +/- SEM are reported. Number of IR puncta analyzed: Sensitive 13,128 puncta, Resistant 14,327 puncta, Resistant 6.25μM

Metformin 12,948 puncta, Resistant 12.5μM Metformin 13,867 puncta, Resistant 50μM Metformin 20,817 puncta. Number of cells analyzed to quantitate the number of IR puncta per cell: Sensitive 4 cells, Resistant 4 cells, Resistant 6.25μM Metformin 4 cells, Resistant 12.5μM Metformin 6 cells, Resistant 50μM Metformin 4 cells. Unpaired two-sided t-test was used for statistical analysis. **b**, Imaging of IR-GFP in insulin-sensitive cells treated with or without 50μM metformin and acutely stimulated with 3nM insulin for 5 minutes. Dashed light blue lines represent nuclear outline. **c,** Immunoblot for IRbeta (IRb) and beta-actin (bActin) in cells cultured in pathologic levels of insulin treated with (RM) or without (R) 12.5μM metformin (left). Quantification of relative levels of IRb in insulin-resistant cells (red) and insulin-resistant cells treated with metformin (purple) (right). 3 biological replicates were analyzed. In the graph, individual values and the mean +/- SEM are reported. Unpaired two-sided t-test was used for statistical analysis. Source data are provided as a Source Data file.

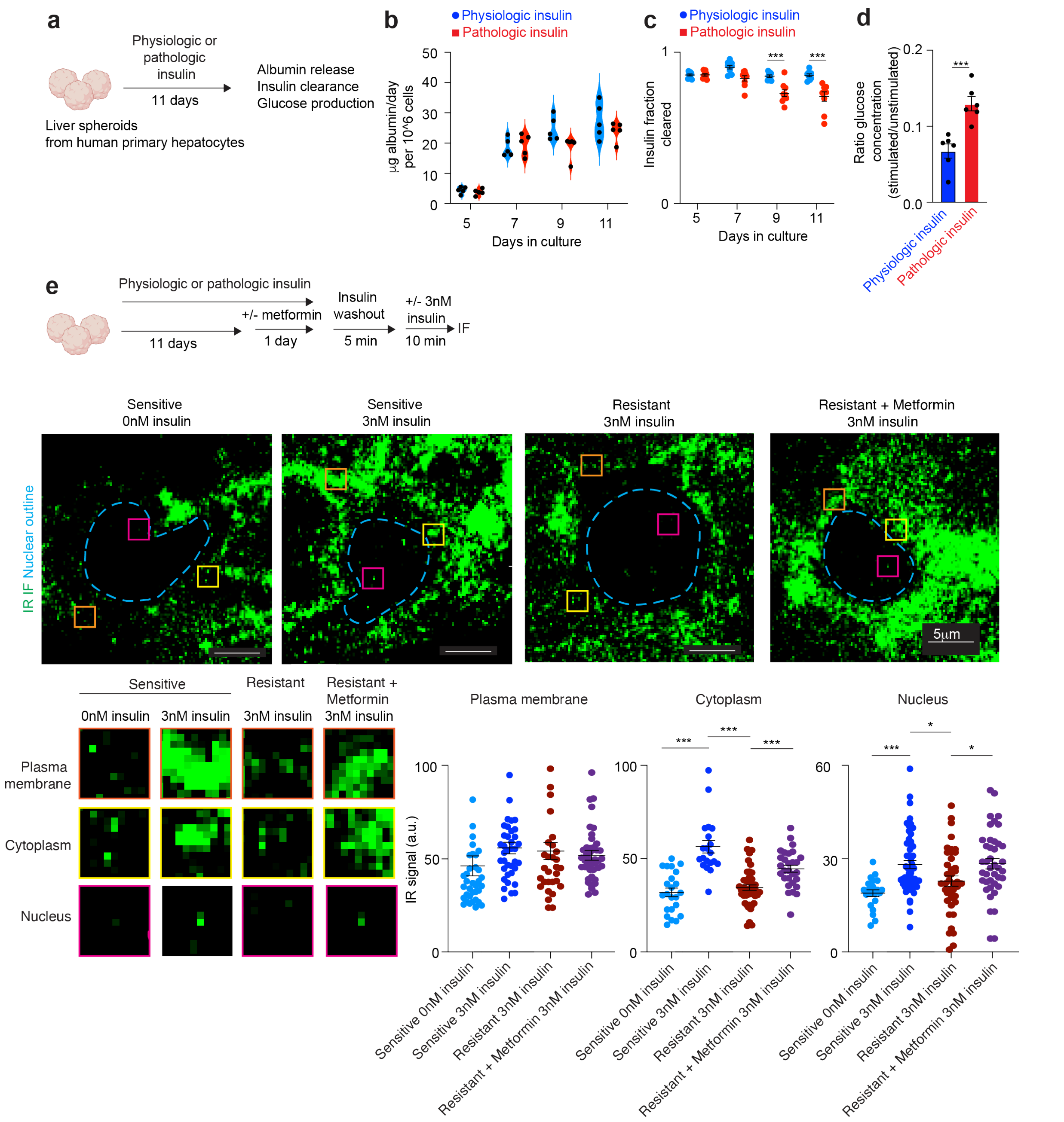

Supplementary Fig. 11. IR puncta in human primary hepatocytes, a, Schematic of cell treatments. **b,** Enzyme-linked immunoassay (ELISA) quantification of albumin production by human liver spheroids cultured with physiologic (blue) or pathologic (red) concentrations of insulin. Individual values are reported in the graph. 5 biological replicates were analyzed. **c,** Enzyme-linked immunoassay (ELISA) quantification of insulin clearance by human liver spheroids cultured with physiologic (blue) or pathologic (red) concentrations of insulin. Individual values and the mean +/- SEM are reported in

the graph. 9 biological replicates were analyzed and unpaired two-sided t-test was used for statistical analysis. **d,** Quantification of glucose production in human liver spheroids cultured with physiologic (blue) or pathologic (red) concentrations of insulin. Individual values and the mean +/- SEM are reported in the graph. 6 biological replicates were analyzed and unpaired two-sided t-test was used for statistical analysis. **e,** Schematic of cell treatments (top). Immunofluorescence for IR in insulin-sensitive, insulin-resistant and metformin-treated insulin-resistant human liver spheroids acutely treated with 0nM or 3nM insulin for 10 minutes (middle). Dashed light blue lines represent nuclear outline. Orange, yellow and magenta boxes represent regions at the plasma membrane, cytoplasm and nucleus, respectively, that are magnified (bottom left). Quantification of IR signal at IR puncta at the plasma membrane, cytoplasm and nucleus of insulin-sensitive hepatocytes (light blue), insulin-sensitive hepatocytes acutely stimulated with insulin (blue), insulin-resistant hepatocytes acutely stimulated with insulin (red) and insulin-resistant hepatocytes treated with metformin and acutely stimulated with insulin (purple) (bottom right). Individual values and the mean +/- SEM are reported in the graph. Number of IR puncta analyzed: Sensitive 0nM insulin Plasma membrane 37 puncta, Cytoplasm 23 puncta, Nucleus 21 puncta; Sensitive 3nM insulin Plasma membrane 38 puncta, Cytoplasm 20 puncta, Nucleus 55 puncta; Resistant 3nM insulin Plasma membrane 32 puncta, Cytoplasm 46 puncta, Nucleus 41 puncta; Resistant + Metformin 3nM insulin Plasma membrane 45 puncta, Cytoplasm 29 puncta, Nucleus 41 puncta. Unpaired two-sided t-test was used for statistical analysis. If the fluorescence made the scale bar hard to see, a black box was added behind the scale bar. Source data are provided as a Source Data file.

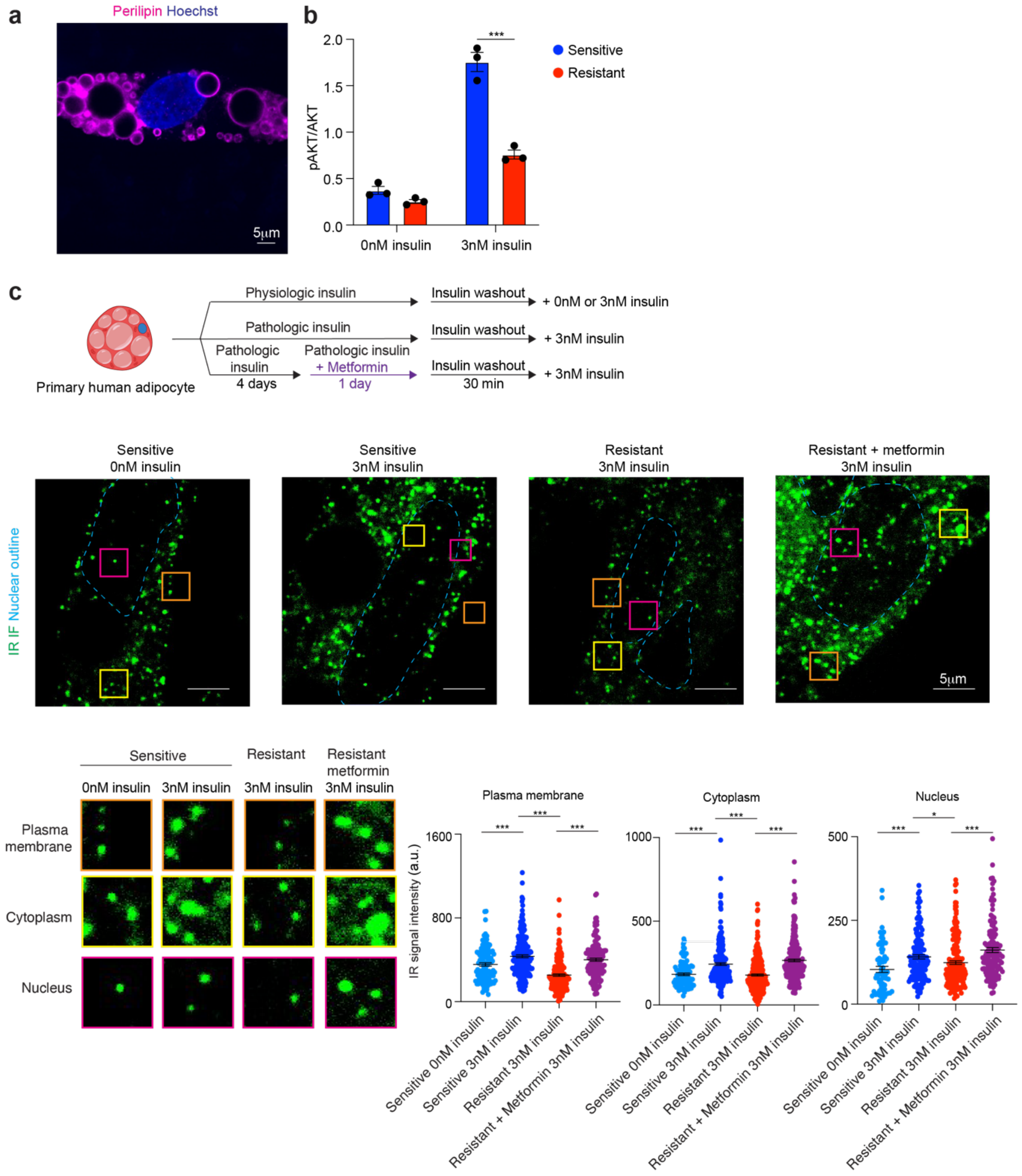

Supplementary Fig. 12. IR puncta in human primary adipocytes. a, Representative immunofluorescence image of perilipin (magenta) in human primary adipocyte. Nucleus is counterstained using Hoechst. **b**, Enzyme-linked immunoassay (ELISA) quantification of pAKT over AKT in human primary adipocytes treated with physiological (Sensitive, blue) or pathological (Resistant, red) concentrations of insulin for 5 days and acutely stimulated (3nM insulin) or not (0nM insulin) with insulin for 15 minutes. 3 biological replicates were analyzed. Individual values are reported in the graph and the bar graph

represent mean +/- SEM. Unpaired two-sided t-test was used for statistical analysis. **c,** Schematic of cell treatments (top). Immunofluorescence for IR in insulin-sensitive (Sensitive), insulin-resistant (Resistant) and metformin-treated insulin-resistant (Resistant + Metformin) human primary adipocytes acutely treated with 0nM or 3nM insulin for 5 minutes (middle). Orange, yellow and magenta boxes represent regions at the plasma membrane, cytoplasm and nucleus, respectively, that are magnified at the bottom left. Quantification of IR signal at IR puncta at the plasma membrane, cytoplasm and nucleus of insulin-sensitive adipocytes (light blue), insulin-sensitive adipocytes acutely stimulated with insulin (blue), insulin-resistant adipocytes acutely stimulated with insulin (red) and insulin-resistant adipocytes treated with metformin and acutely stimulated with insulin (purple) (bottom right). (bottom right). Individual values and the mean +/- SEM are reported in the graph. Number of IR puncta analyzed: Sensitive 0nM insulin Plasma membrane 107 puncta, Cytoplasm 91 puncta, Nucleus 67 puncta; Sensitive 3nM insulin Plasma membrane 209 puncta, Cytoplasm 238 puncta, Nucleus 135 puncta; Resistant 3nM insulin Plasma membrane 178 puncta, Cytoplasm 274 puncta, Nucleus 187 puncta; Resistant + Metformin 3nM insulin Plasma membrane 148 puncta, Cytoplasm 279 puncta, Nucleus 129 puncta. Unpaired two-sided t-test was used for statistical analysis. Source data are provided as a Source Data file.

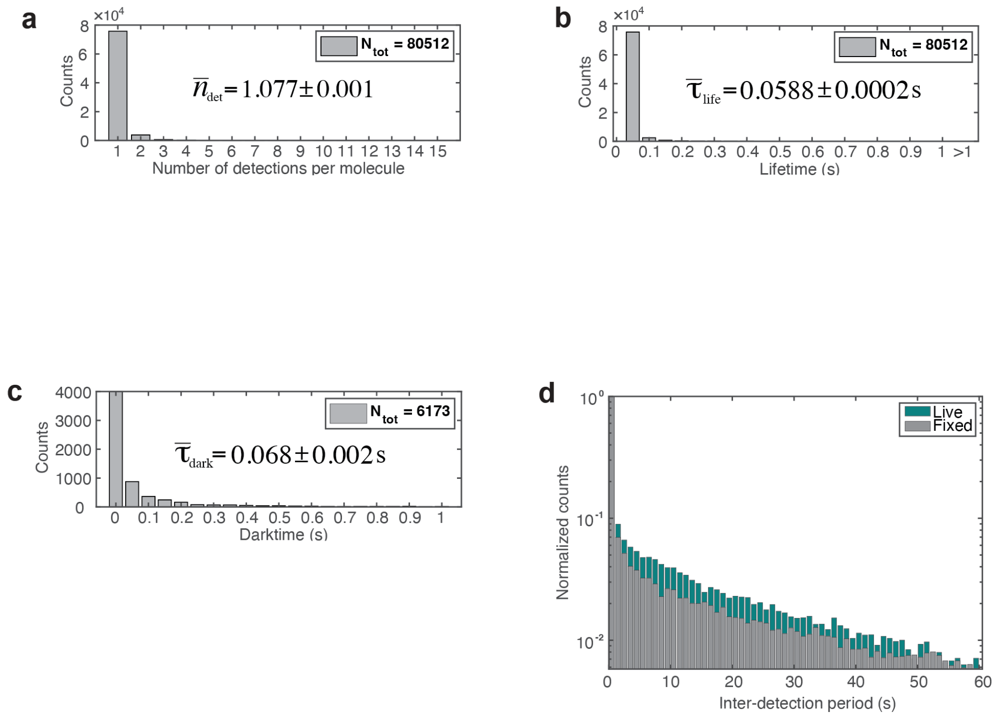

e

Statistics of overall single molecule (SM)	Live	Fixed	Units
Avg. number of detections per SM	1.077 ± 0.001	1.204 ± 0.004	counts
Avg. duration time of overall SM	0.059 ± 0.000	0.075 ± 0.001	second
Avg. inter-detection period of multi-detection SM	0.068 ± 0.002	0.079 ± 0.002	second

f

Statistics of identified multimolecule bursts	AVG.		STD.	Units
Number of identified bursts per 10,000 detections	67.02	±	2.26	counts
Avg. duration time ("lifetime") of bursts	14.52	±	0.23	second
Avg. inter-detection period of bursts	1.43	±	0.02	second
Avg. number of detections per burst	22.46	±	0.78	counts
Lifetime of bursts — 0.05 quantile	0.85	±	0.03	second
Number of detections per burst — 0.05 quantile	4.00	±	0.00	counts

Statistics of outlier SM (t_min=0.85s, n_min=4)	AVG.		STD.	Units
Number of false positive SM per 10,000 detections	4.67	±	0.66	counts
Avg. duration time of false positive SM	1.21	±	0.06	second
Avg. inter-detection period of false positive SM	0.24	±	0.02	second
Avg. number of detections per false positive SM	5.65	±	0.37	counts

TPR>90%

Supplementary Fig. 13. Single-molecule statistics and validation of tc-PALM analysis. a, Distribution of the number of detections of single molecules in live cells. Total number of 80512 single molecules are collected for plotting the histogram. **b**, Distribution of the lifetime of single molecules. **c,** Distribution of the inter-detection period (dark-time) of single molecules with more than one detection. Total number of 6173 multi-detection

single molecules are collected for plotting the histogram. **d,** Histogram of inter-detection period of identified transient clusters in live cells and pseudo-transient clusters in fixed cells selected with the same procedure as in live cells. The counts of each bin are normalized to the first bin, which mostly consists of counts of blinking events from single molecules (given that most single molecules have a lifetime span shorter than 1s). **e,** Statistics of single molecules in live and fixed samples. **f,** Statistics of identified multimolecule bursts and outlier single molecules. Ideally, the true positive rate (TPR) can go beyond 90% based on the estimation of cut-offs (0.05 quantile) from real bursts. Source data are provided as a Source Data file.

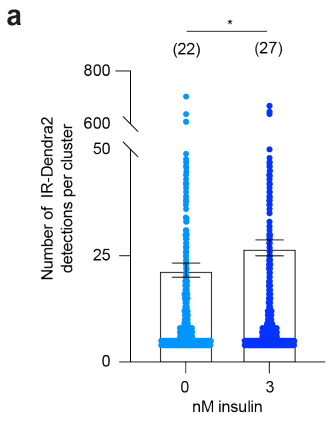

Supplementary Fig. 14. IR-Dendra2 detections in clusters throughout the cell. a, Quantification of the number of IR-Dendra2 detections per IR cluster in insulin-sensitive cells stimulated with (3nM, dark blue) and without (0nM, light blue) insulin for 5 minutes. Average number of IR-Dendra2 detections per IR cluster is reported in parenthesis on top of each histogram. Histograms represent mean +/- SEM. Number of clusters analyzed: Sensitive 0nM insulin 908 clusters, Sensitive 3nM insulin 1,116 clusters. Unpaired two-sided t-test was used for statistical analysis. Source data are provided as a Source Data file.

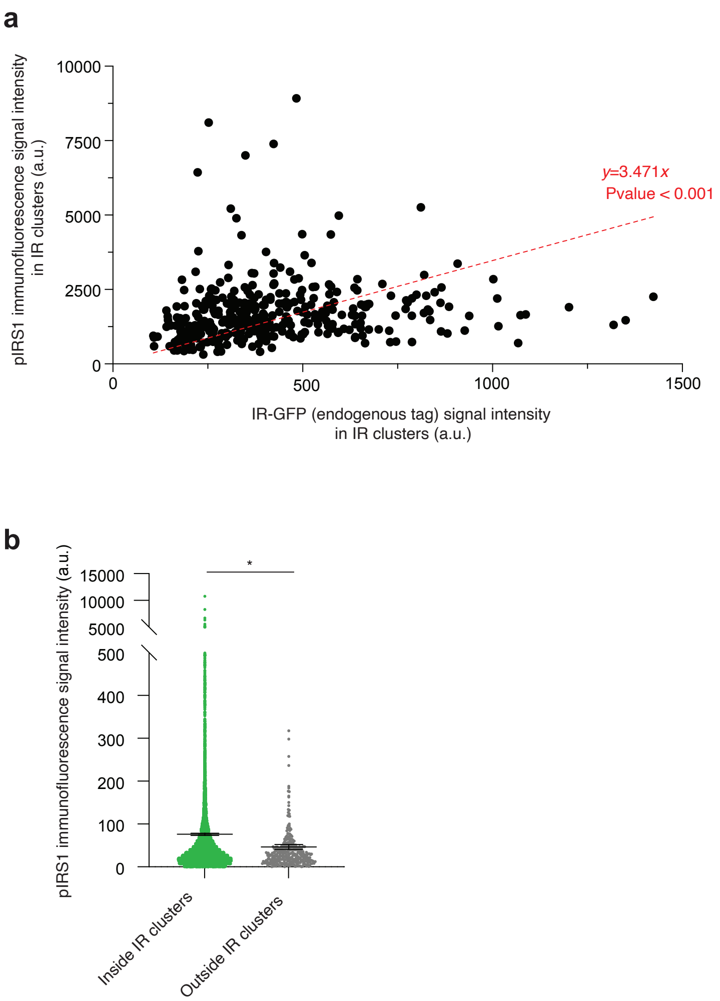

Supplementary Fig. 15. Correlation between IR and pIRS1 signal intensity in clusters. a, Quantification of pIRS1 and IR signal in clusters. To obtain IR clusters with different levels of IR molecules, HepG2 cells expressing endogenous IR tagged with GFP (IR-GFP) were treated with siControl or siRNA for INSR for 18 hours or 24 hours. 391 IR clusters were analyzed. Linear regression was used to generate trendline. Equation: y=3.47x. **b,** Quantification of pIRS1 signal inside (green) and outside (grey) IR clusters. Individual values and the mean +/- SEM are reported in the graph. 12,447 IR clusters and 397 regions outside of IR clusters were analyzed. Unpaired two-sided t-test was used for statistical analysis. Source data are provided as a Source Data file.

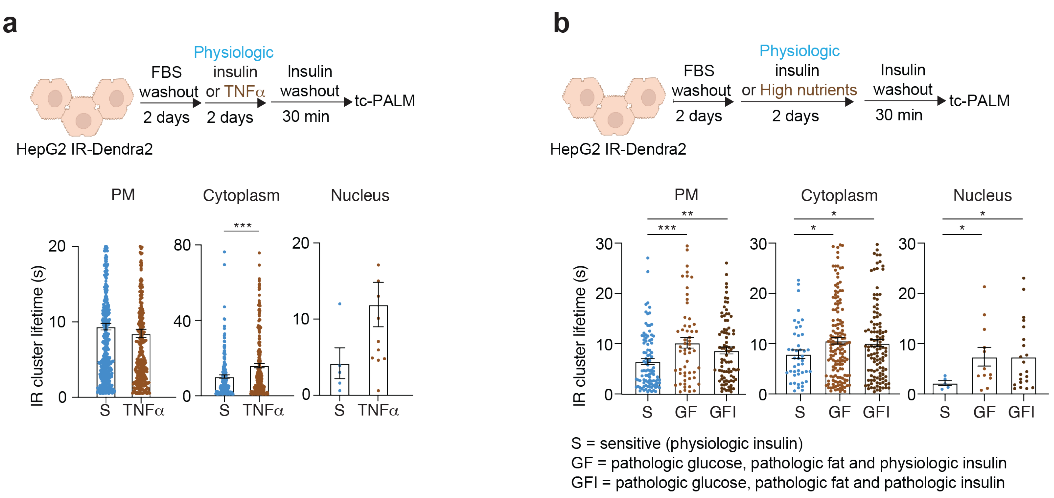

Supplementary Fig. 16. Increased IR cluster lifetime by inflammation and high nutrients. a, Schematic of cell treatments (top). Tc-PALM quantification of IR cluster lifetime at the plasma membrane (PM), cytoplasm and nucleus in HepG2 cells expressing IR-Dendra2 treated with physiological concentrations of insulin (Sensitive, S, light blue) or pathological concentration of TNFα (TNFα, brown). Individual values and the mean +/- SEM are reported in the graph. Number of short-lived clusters analyzed: Plasma membrane Sensitive 440 clusters, TNFα 307 clusters; Cytoplasm Sensitive 170 clusters, TNFα 212 clusters; Nucleus Sensitive 5 clusters, TNFα 22 clusters. Unpaired two-sided t-test was used for statistical analysis. **b**, Schematic of cell treatments (top). Tc-PALM quantification of IR cluster lifetime at the plasma membrane (PM), cytoplasm and nucleus in HepG2 cells expressing IR-Dendra2 treated with physiological concentrations of insulin (Sensitive, S, light blue) or with high nutrients either 1) pathological concentrations of glucose and fat and physiological concentration of insulin (high nutrients, GF, brown) or 2) pathological concentrations of glucose, fat and insulin (high nutrients, GFI, dark brown). Physiological concentration of insulin corresponds to 0.1nM, pathological concentration of insulin corresponds to 3nM, pathological concentration of TNFα corresponds to 100pg/ml, pathological concentration of fat corresponds to 30μM palmitic acid and 45μM oleic acid, pathological concentration of glucose corresponds to 10mM. Number of short-lived clusters analyzed: Plasma membrane Sensitive 97 clusters, GF 56 clusters, GFI 85 clusters; Cytoplasm Sensitive 44 clusters, GF 148 clusters, GFI 117 clusters; Nucleus Sensitive 5 clusters, GF 12 clusters, GFI 21 clusters. Unpaired two-sided t-test was used for statistical analysis (Plasma membrane Sensitive vs GF, Sensitive vs GFI; Cytoplasm

Sensitive vs GF) or unpaired one-sided t-test was used for statistical analysis for cytoplasm Sensitive vs GFI and nucleus Sensitive vs GF, Sensitive vs GFI. Source data are provided as a Source Data file.

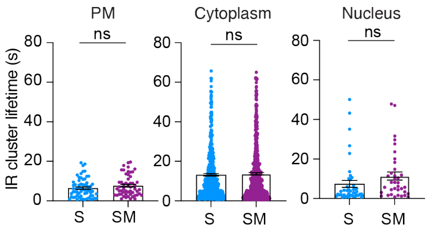

Supplementary Fig. 17. Metformin does not decrease IR cluster lifetime in insulin-sensitive cells. Tc-PALM quantification of IR cluster lifetime at the plasma membrane (PM), cytoplasm and nucleus in insulin-sensitive HepG2 cells expressing IR-Dendra2 treated with (purple) and without 12.5μM metformin (light blue) for 1 day. Data is represented as mean +/- SEM. Number of IR short-lived clusters analyzed: Sensitive (S) Plasma membrane 310 clusters, Cytoplasm 456 clusters, Nucleus 43 clusters; Sensitive + Metformin (SM) Plasma membrane 447 clusters, Cytoplasm 609 clusters, Nucleus 36 clusters. Unpaired two-sided t-test was used for statistical analysis.

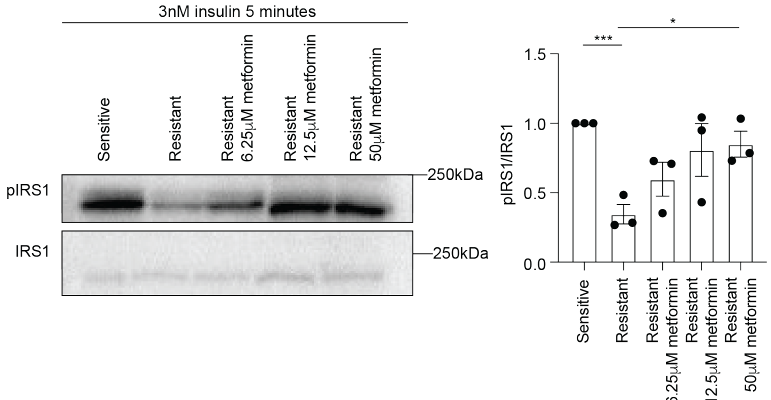

Supplementary Fig. 18. Metformin partially rescues phosphorylation of IRS1. Immunoblot and quantification of pIRS1 over total IRS1 in insulin-sensitive, insulin-resistant and metformin-treated insulin-resistant cells. Metformin concentrations used in the experiment are reported in the image. Data is represented as single values and bar graphs (mean +/- SEM). Three biological replicates were analyzed. Unpaired two-sided t-test was used for statistical analysis. Source data are provided as a Source Data file.

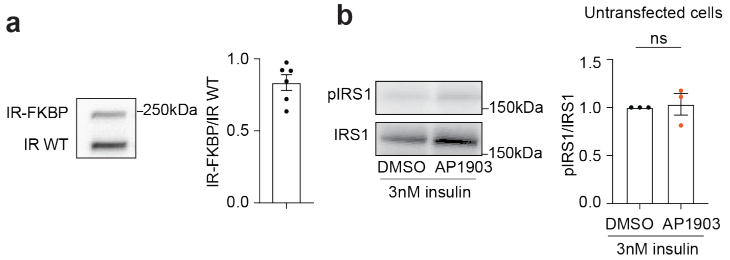

Supplementary Fig. 19. Effect of AP1903 on HepG2 cells. a, Immunoblot and quantification of the relative levels of expression of WT IR (IR WT) and IR-GFP-FKBP (IR-FKBP). Data is represented as single values and a bar graph (mean +/- SEM). 6 biological replicates were analyzed. **b**, Immunoblot and quantification of the relative levels of phosphorylated IRS1 and total IRS1 in untransfected, wildtype HepG2 cells treated with DMSO or AP1903. 3 biological replicates per condition were analyzed. Data is represented as single values and bar graphs (mean +/- SEM). Unpaired two-sided t-test was used for statistical analysis. Source data are provided as a Source Data file.

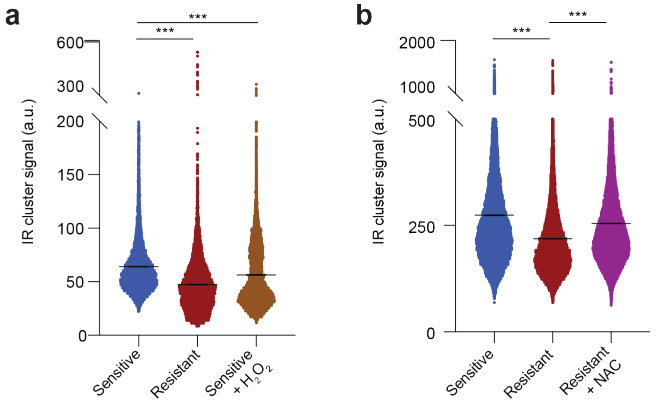

Supplementary Fig. 20. **Oxidative stress effect on IR incorporation into clusters. a**, Quantification of IR signal intensity in IR clusters in the entire cell (automated quantification, without specifying cellular subcompartments) relative to Fig. 5c. Data points from sensitive cells are represented in blue, data points from resistant cells are represented in dark red, data points from resistant cells treated with NAC are represented in dark red. Single values and mean +/- SEM are shown. Number of clusters analyzed: Sensitive 11,110 clusters, Resistant 8,861 clusters, Sensitive + H_2O_2 8,068 clusters. Unpaired two-sided t-test was used for statistical analysis. **b**, Quantification of IR signal intensity in IR clusters in the entire cell (automated quantification, without specifying cellular subcompartments) relative to Fig. 5e. Single values and mean +/- SEM are

shown. Number of clusters analyzed: Sensitive 29,398 clusters, Resistant 30,600 clusters, Resistant + NAC 46,992 clusters). Unpaired two-sided t-test was used for statistical analysis. Source data are provided as a Source Data file.

Healthy/T2D	Specimen ID	Case ID	Age At Excision	Sex	Etnicity	Biosample Diagnosis	BMI	Metformin Treatment	Source
Healthy	1208572F	87808	77	Female	Caucasian	Normal	23.14	No	BioIVT
Healthy	1208568F	87808	77	Female	Caucasian	Normal	23.14	No	BioIVT
Healthy	FHU-L-102319	N/A	37	Female	Caucasian	Normal	23.82	No	BioIVT
Healthy	AM-092	N/A	29	Female	Caucasian	Normal	24	No	MGH
Healthy	20-018 / OL-001	N/A	67	Male	Caucasian	Normal	23	No	MGH
Healthy	21-015/OL-035	N/A	50	Male	N/A	Normal	25	No	MGH
Healthy	AM-019/OL-028	N/A	50	Female	Caucasian	Normal	28	No	MGH
T2D	1214825F	99117	53	Female	Caucasian	Steatosis	27.18	No	BioIVT
T2D	1137920F	47111	70	Male	Caucasian	Steatosis	34.42	No	BioIVT
T2D	1143147F	49893	76	Female	N/A	Congestion	37.6	No	BioIVT
T2D	AM-026/OL-038	N/A	70	Male	Caucasian	Steatosis	38	No	MGH
T2D	20-024 / OL-004	N/A	73	Male	Caucasian	Unkown	23	No	MGH
T2D	OL-019	N/A	73	Male	Caucasian	Steatosis	22	No	MGH
T2D	20210519	N/A	62	Male	Hispanic	Congestion	36.6	No	MGH
T2D	1096575F	48612	66	Male	Caucasian	Steatosis	30.86	Yes	BioIVT
T2D	1153543F	52473	77	Female	Native American or Alaskan Native	Steatosis	25.7	Yes	BioIVT
T2D	27534H1	14426	59	Female	Caucasian	Steatosis	20.9	Yes	BioIVT
T2D	AM-011 / OL-025	N/A	54	Male	Caucasian	Steatosis	42	Yes	MGH
T2D	OL-013	N/A	72	Male	Caucasian	Steatosis	33.5	Yes	MGH
T2D	21-006/OL-029	N/A	69	Male	Caucasian	Steatosis	31.5	Yes	MGH
T2D	21-134/OL-063	N/A	67	Male	Caucasian	Steatosis	31	Yes	MGH
T2D	20-054 / OL-024	N/A	41	Male	Caucasian	Steatosis	36	Yes	MGH
T2D	21-088/OL-052	N/A	69	Female	Caucasian	Steatosis	28	Yes	MGH

Supplementary Table 1. Donor characteristics.

SUPPLEMENTAL TEXT

Photochemistry of single Dendra2 molecules

Given that there could be an ambiguous mapping from the number of detections to the number of Dendra2 molecules, several control analyses of the single molecule photochemistry have to be done to validate the statistics of the real clusters (which ideally consist of colocalized, time-correlated, multimolecule bursts). Imaging of IR in either fixed or live IR-Dendra2 cells was performed in L-15 medium using the same laser setups as described above. After the same ROI was imaged for a long time, most Dendra2 molecules were photo-converted and bleached, whereupon the rest of intact single molecules were sparsely photo-converted and recorded, and the consequent colocalized detections from the same molecule can be well spatiotemporally isolated and grouped. The statistics of live-cell Dendra2 single molecules are shown in Supplementary Figure 13a-c. The comparisons of Dendra2 single molecules in live and fixed samples are shown in Supplementary Figure 13e. 94% of the single molecules only generate one detection (Supplementary Fig. 13a), which results in the average number of detections per molecule being close to one ($\bar{n}_{\mathrm{det}} \approx 1.077$). The average lifetime of single molecules is 0.059s, and only 1% of them has a lifetime longer than 0.25s (Supplementary Fig. 13b). Among those multiple-detection molecules, 65% of them result in the same emitting event occupying two adjacent frames (Supplementary Fig. 10c), and the real average dark-time between blinking events is around 0.2s.

Validation of the existence of dynamic clustering in live cells

We identified pseudo-transient clusters in fixed cells with the exact procedures and criteria as for searching transient clusters in live cells. For spatially clustered structures, significantly larger dark times in live cells, compared to fixed cells under identical condition, is a sign of the bursting dynamics in live cells [8]. This is exactly what we observed (Supplementary Fig. 13d), and such larger dark times of clusters in live cells cannot be explained by longer intrinsic inter-detection period of Dendra2 single molecules in live-cell samples (Supplementary Fig. 13e). Furthermore, we normalized the number of tc-PALM identified bursts by the total number of detections of the same ROI, thus are able to estimate the number of identified bursts per 10,000 detections as 67.02 (Supplementary Fig. 13f). Meanwhile, among the tc-PALM identified bursts, we obtained the number of detections and lifetime of the 0.05 quantile at the lower-bound side as

4 and 0.85s, respectively (Supplementary Fig. 13f). If we use these two numbers as the cut-off for the set of Dendra2 single molecules we measured in live samples, only 4.67 molecules among 10,000 detections can pass the threshold. This indicates that the true positive rate (TPR) can easily go beyond 90%: 67.02÷(67.02+4.67)=93.5%; even in the worst case (all the bursts below the 0.05 quantile were single molecules), the corresponding TPR is 93.5%×95%≈89%. Even for the outlier single molecules that pass the cut-off, their statistics (including duration time, inter-detection period, and number of detections) are still quite different from the of tc-PALM identified bursts (Supplementary Fig. 13f). In another extreme test, we applied several additional high cut-offs to the tc-PALM identified bursts (in some cases, the TPR was pushed to 98%), whereupon we are still able to recapitulate all the significant trends of lifetime-shifting in cytoplasm and nuclei after different perturbations. This observation is reasonable: given that IR molecules are much less abundant in the cytoplasm and nuclei, un-clustered background of randomly bound IR molecules can be safely ignored. Therefore, any time-corelated, multi-detection events inside in the cytoplasm or nuclei are very likely to result from real clusters, which are insensitive to the FPR cut-off. Gathering all these evidences together, we are able to validate the existence of multi-molecule dynamical clustering of IR molecules in live cells, which yields transient bursting dynamics with distinct properties than single molecules and are robustly, physiologically responsive.

SUPPLEMENTARY REFERENCES

1. Ramakrishnan G, Arjuman A, Suneja S, Das C, Chandra NC. The association between insulin and low-density lipoprotein receptors. *Diab Vasc Dis Res* **9**, 196-204 (2012).

2. Hancock ML*, et al.* Insulin Receptor Associates with Promoters Genome-wide and Regulates Gene Expression. *Cell* **177**, 722-736 e722 (2019).

3. Foretz M, Guichard C, Ferre P, Foufelle F. Sterol regulatory element binding protein-1c is a major mediator of insulin action on the hepatic expression of glucokinase and lipogenesis-related genes. *Proc Natl Acad Sci U S A* **96**, 12737-12742 (1999).

4. Hillgartner FB, Salati LM, Goodridge AG. Physiological and molecular mechanisms involved in nutritional regulation of fatty acid synthesis. *Physiol Rev* **75**, 47-76 (1995).

5. Fortez M*, et al.* ADD1/SREBP-1c is required in the activation of hepatic lipogenic gene expression by glucose. *Mol Cell Biol* **19**, 3760-3768 (1999).

6. Claycombe KJ*, et al.* Insulin increases fatty acid synthase gene transcription in human adipocytes. *Am J Physiol* **274**, R1253-1259 (1998).

7. Dif N, Euthine V, Gonnet E, Laville M, Vidal H, Lefai E. Insulin activates human sterol-regulatory-element-binding protein-1c (SREBP-1c) promoter throug h SRE motifs. *Biochem J* **400**, 179-188 (2006).

8. Cisse, II*, et al.* Real-time dynamics of RNA polymerase II clustering in live human cells. *Science* **341**, 664-667 (2013).
