# Supplementary material for: The dynamic clustering of insulin receptor underlies its signaling and is disrupted in insulin resistance
Source: Nat Commun. 2022 Dec 6;13:7522. doi: 10.1038/s41467-022-35176-7 (PMC9727033; doi:10.1038/s41467-022-35176-7)
Supplement: Supplementary file 4 — Source Data [file 41467_2022_35176_MOESM4_ESM.zip › Source_Data_files/Source_Data_WB.pdf]

Extended Data Figure 21: Western blots

Related to Figure 3i

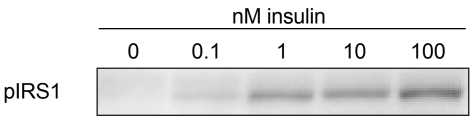

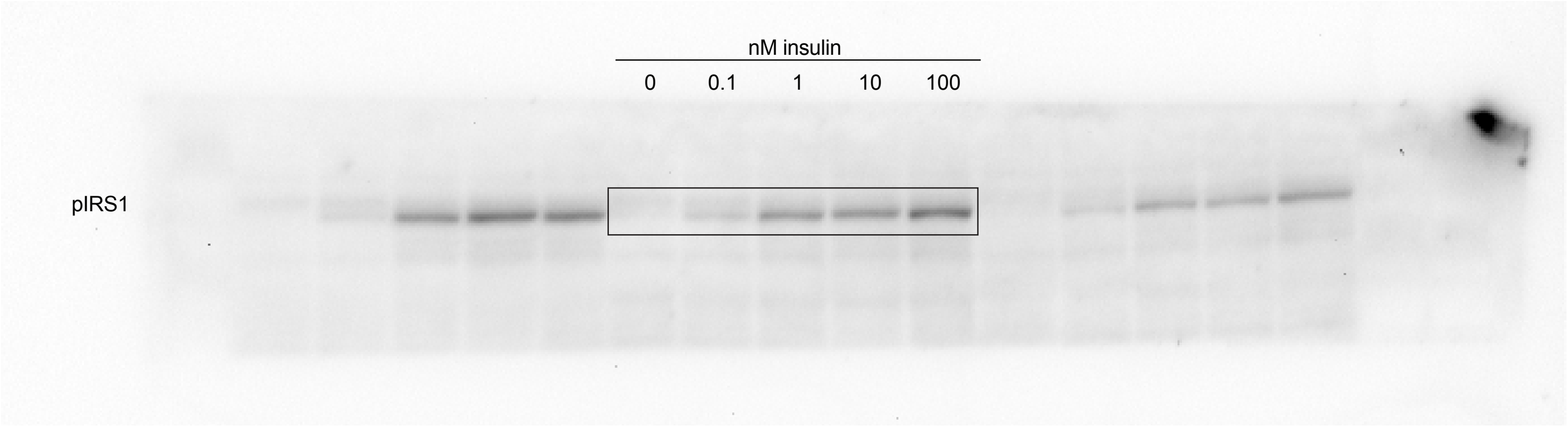

Related to Figure 3i

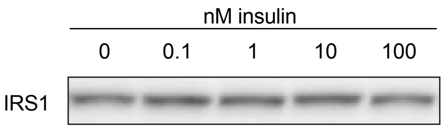

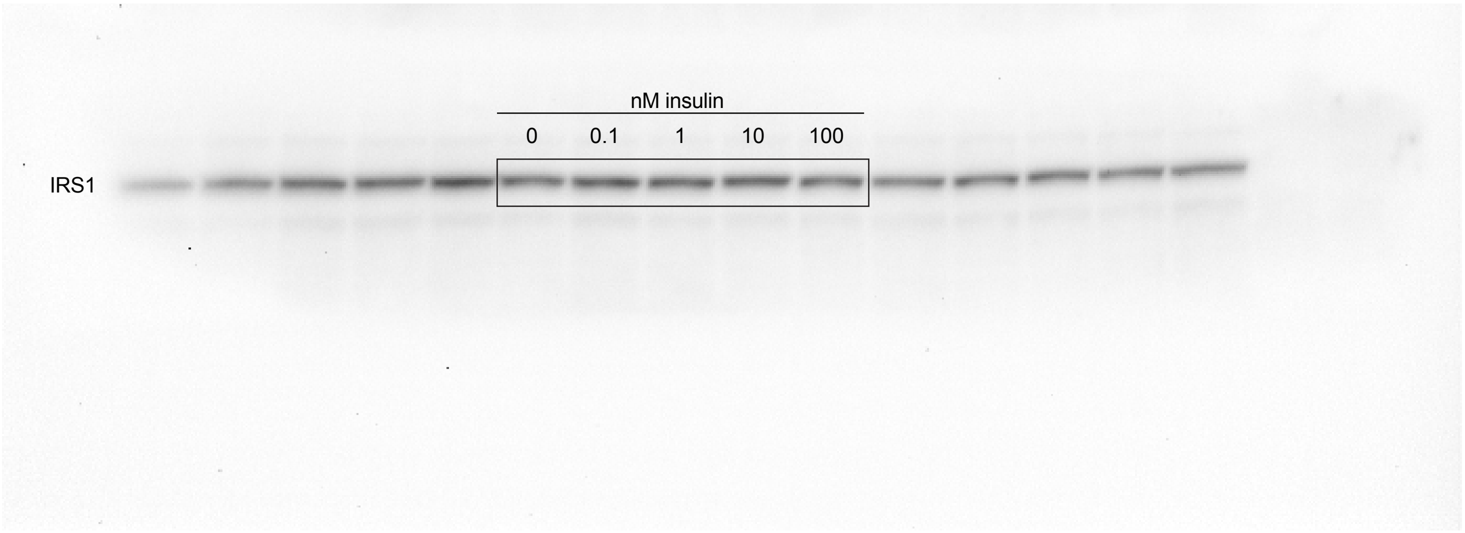

Related to Figure 4g

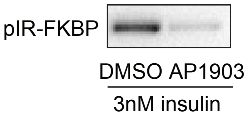

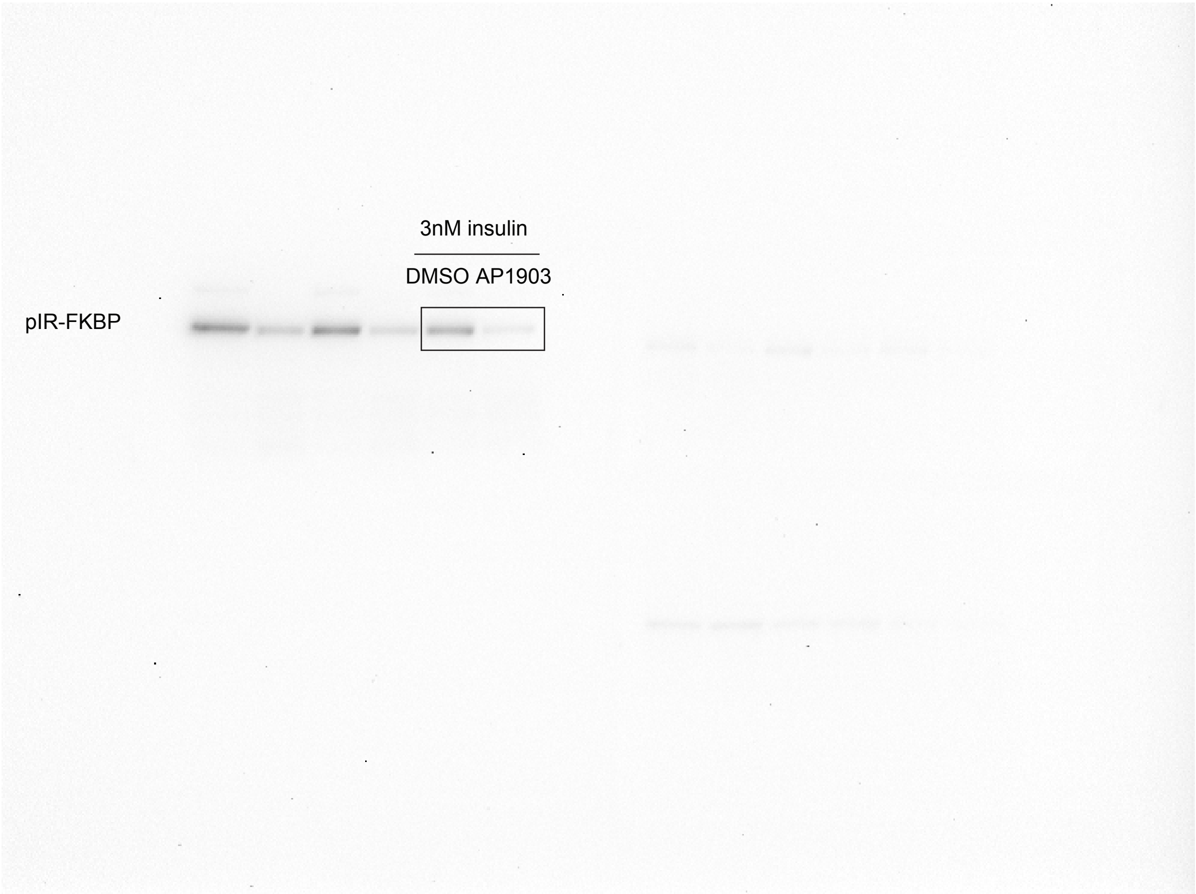

Related to Figure 4g

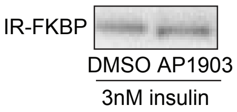

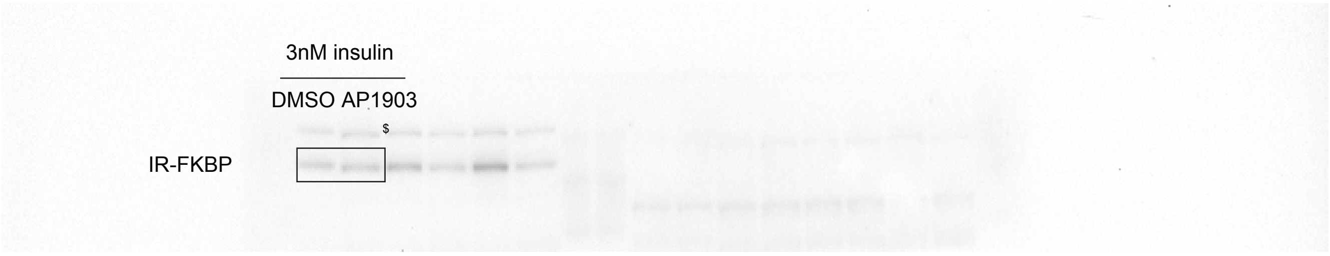

$IR-FKBP proform

Related to Figure 4g

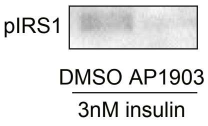

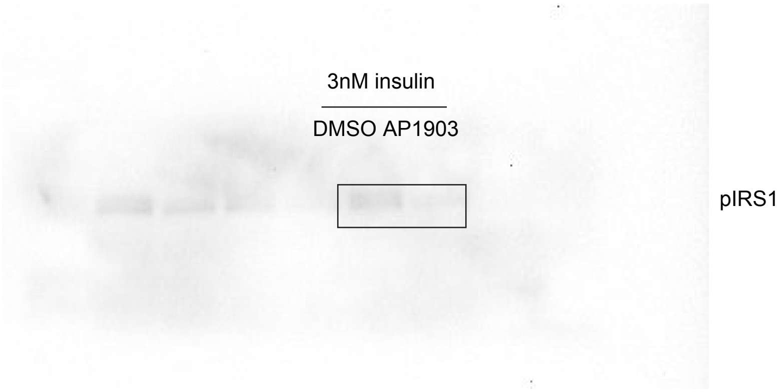

Related to Figure 4g

IRS1 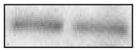

DMSO AP1903

3nM insulin

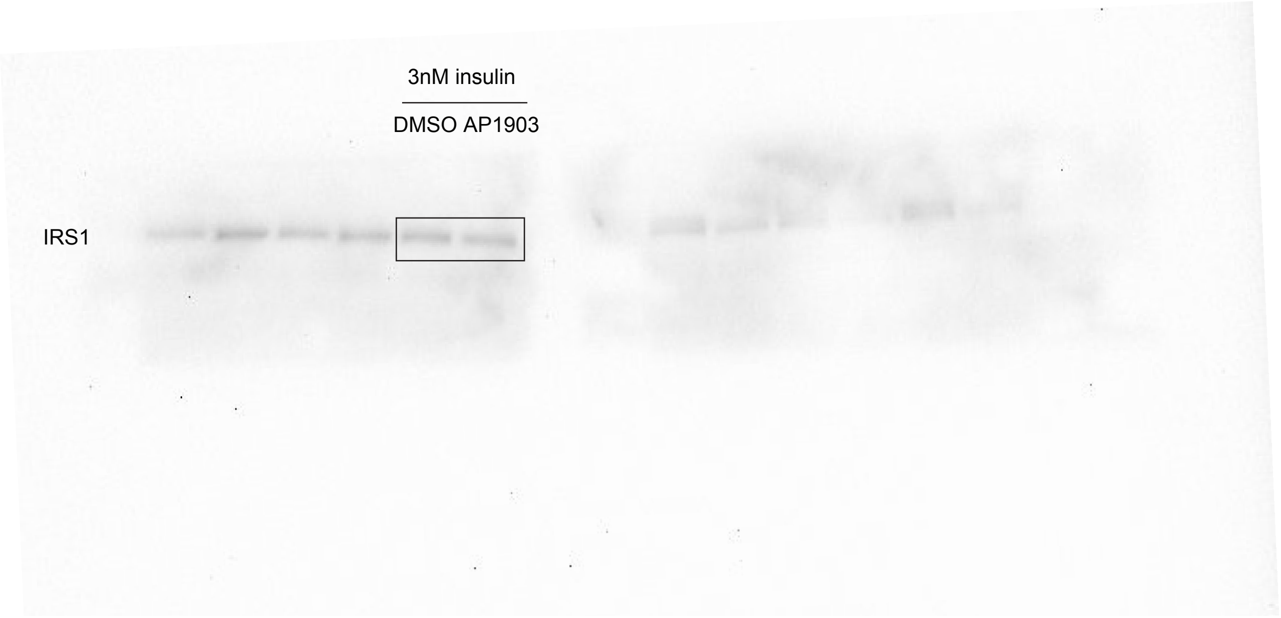

Related to Extended Data Figure 1d

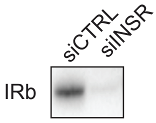

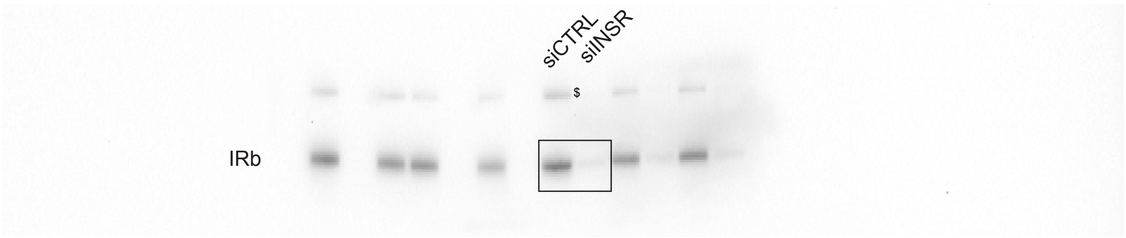

$IR proform

Related to Extended Data Figure 1d

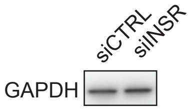

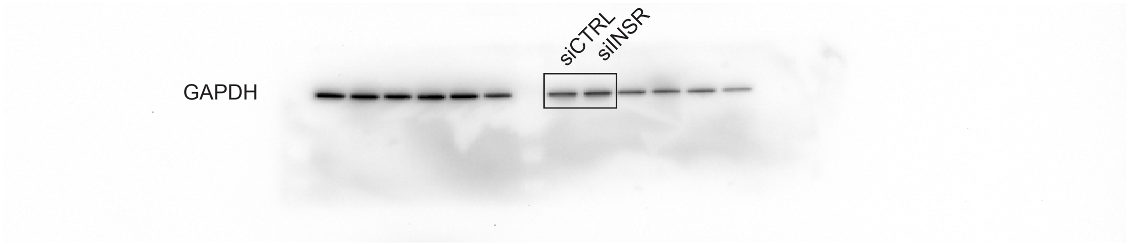

Related to Extended Data Figure 2c

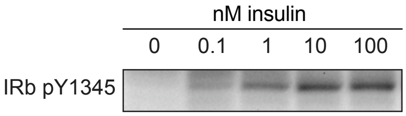

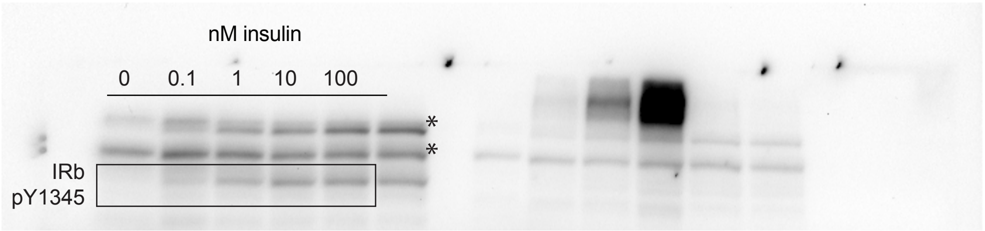

*Unspecific bands

Related to Extended Data Figure 2c

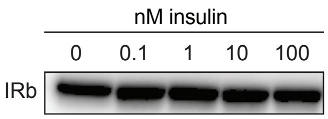

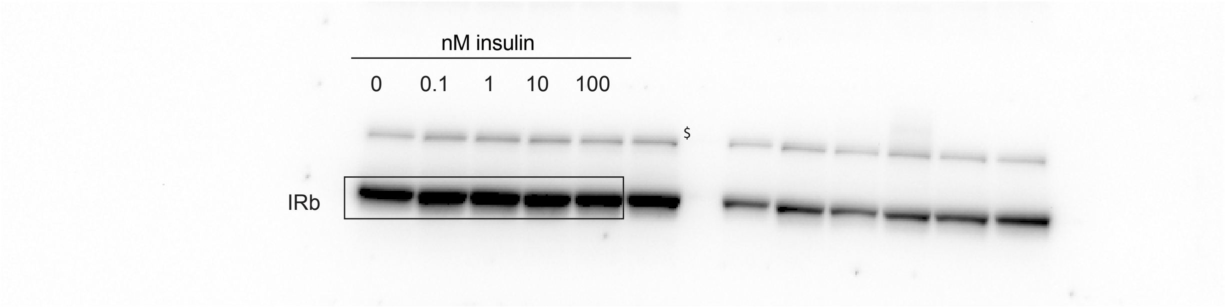

$ IR proform

Related to Extended Data Figure 2c

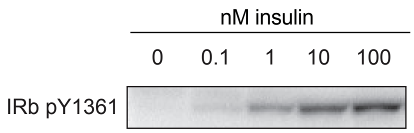

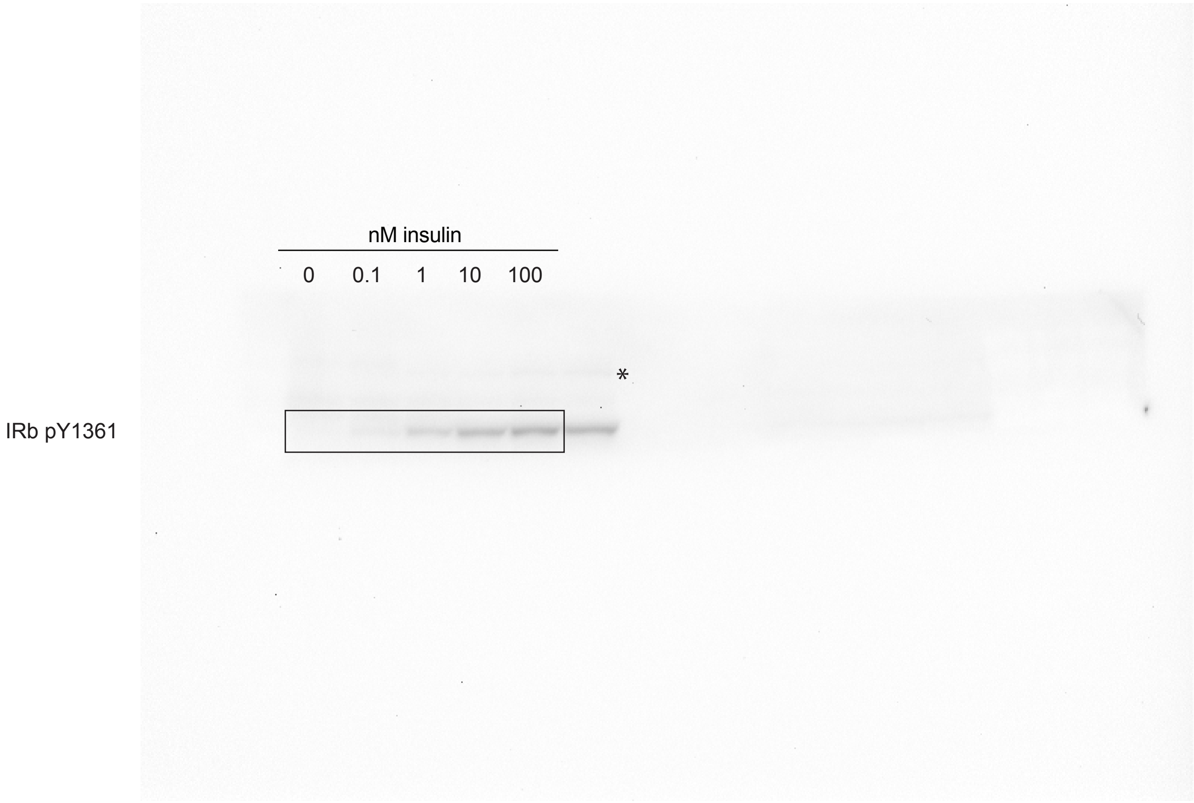

*Unspecific bands

Related to Extended Data Figure 2c

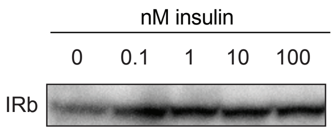

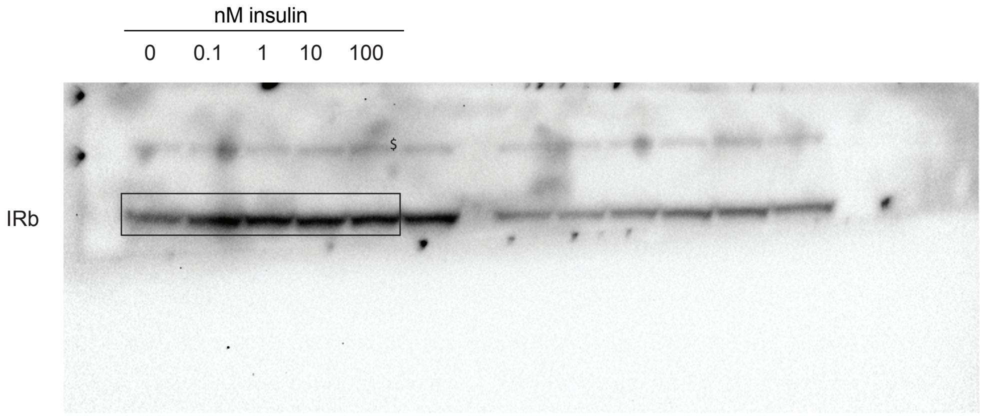

$ IR proform

Related to Extended Data Figure 2c

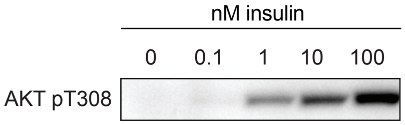

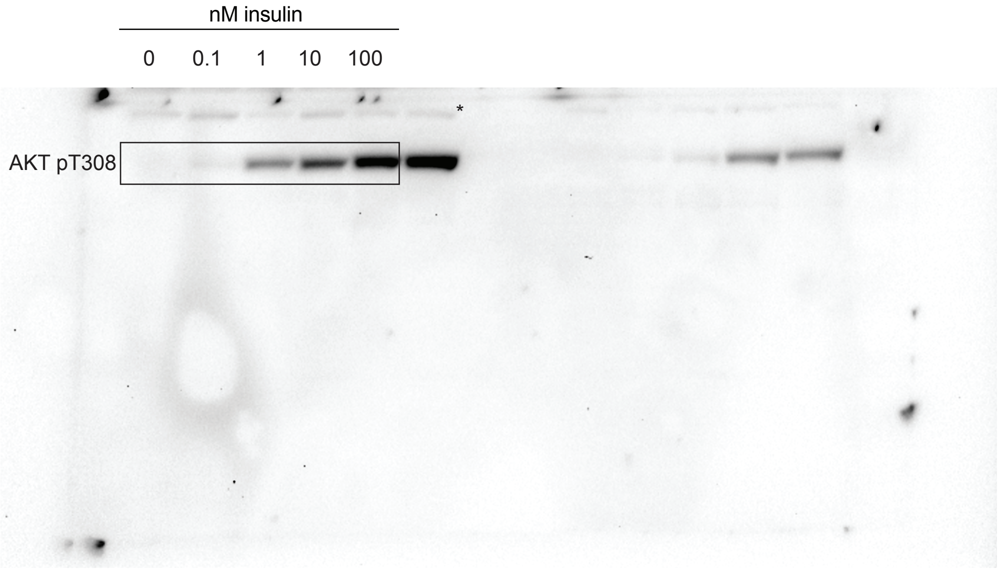

* Unspecific band

Related to Extended Data Figure 2c

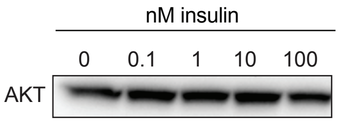

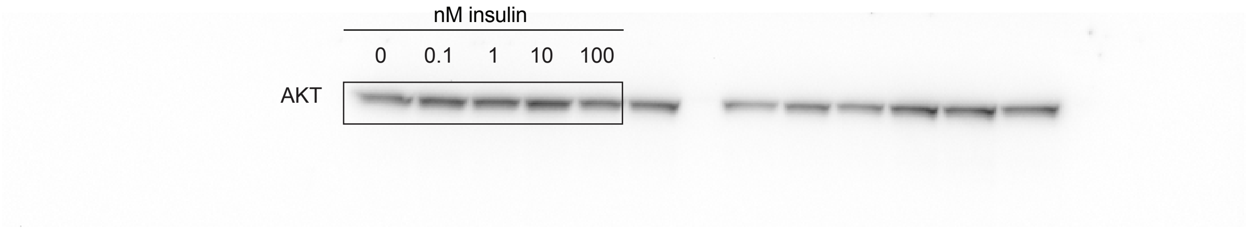

Related to Extended Data Figure 2c

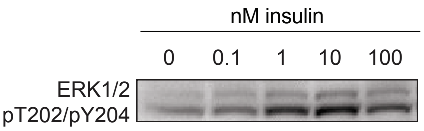

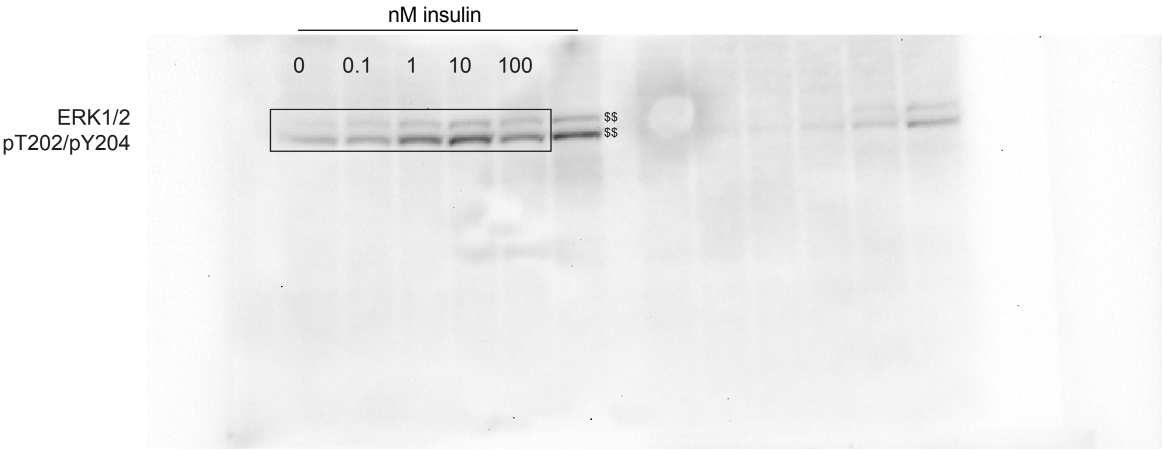

$$ Both bands are pERK1/2

Related to Extended Data Figure 2c

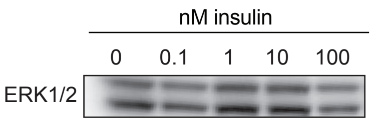

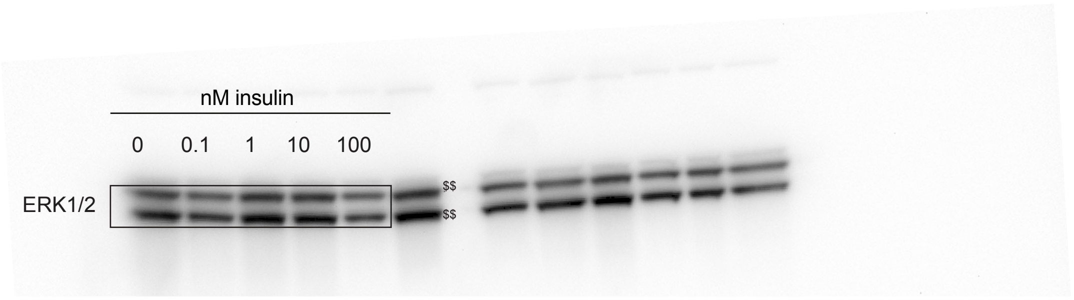

$$ Both bands are ERK1/2

Related to Extended Data Figure 2i

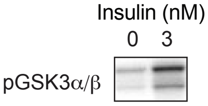

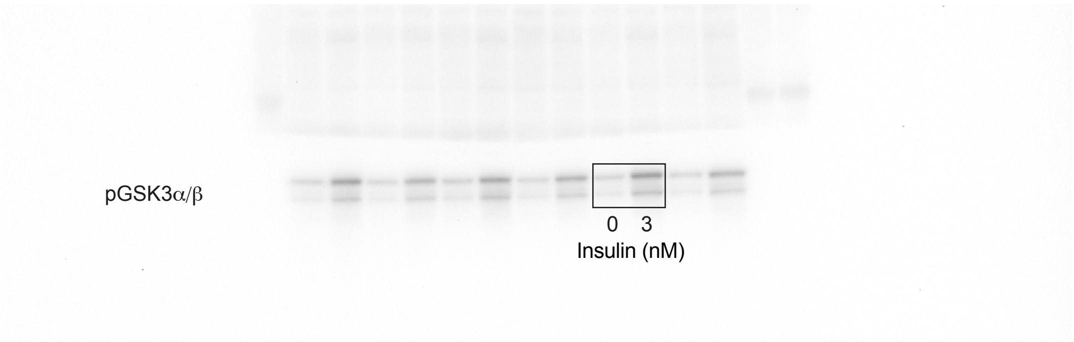

Related to Extended Data Figure 2i

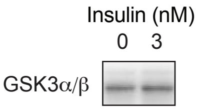

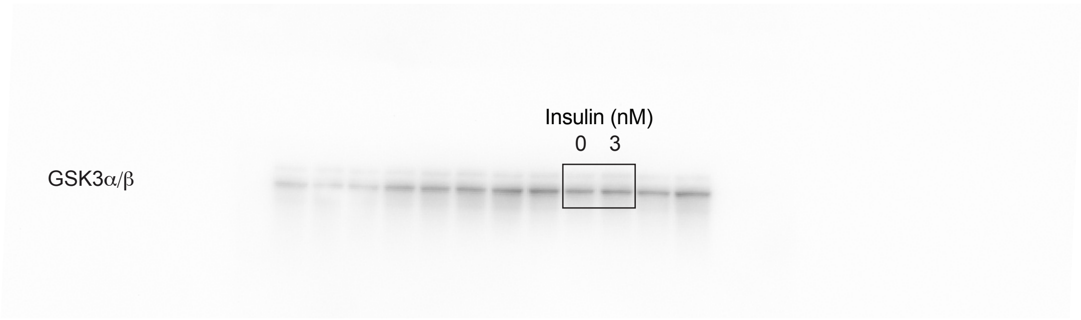

Related to Extended Data Figure 4a

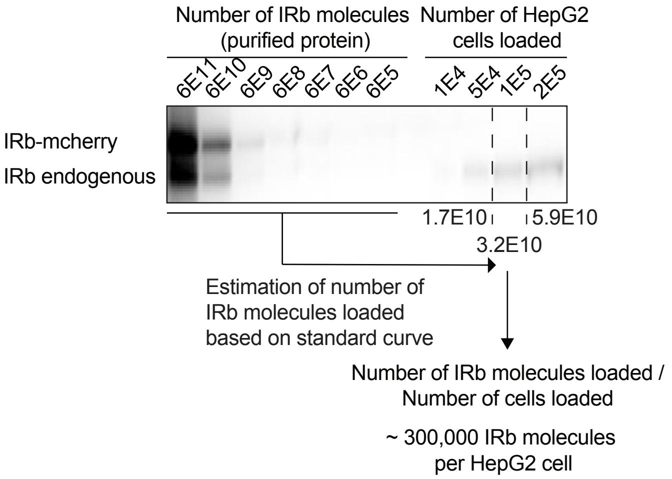

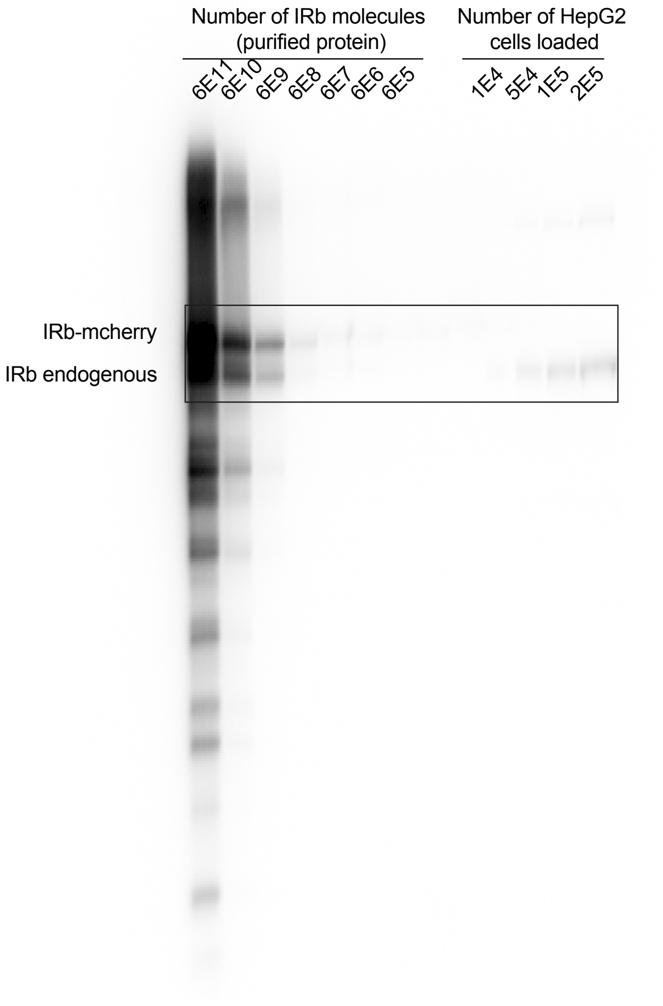

Related to Extended Data Figure 4b

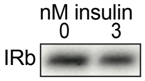

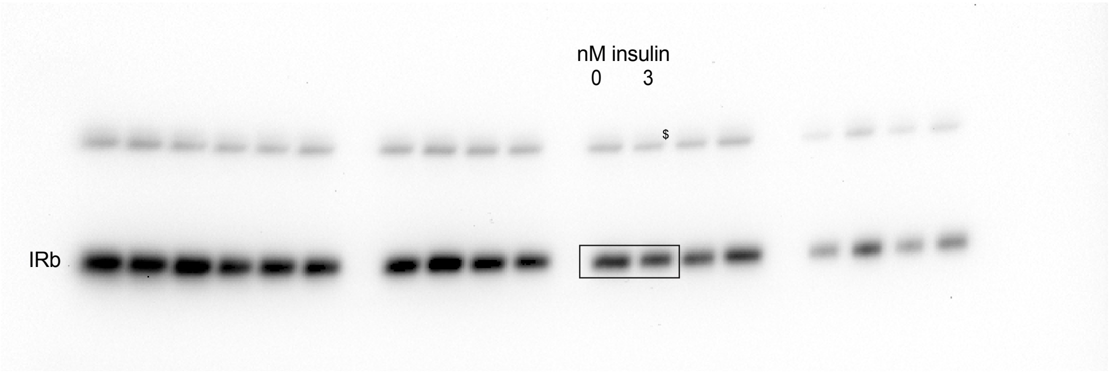

$ IR proform

Related to Extended Data Figure 4b

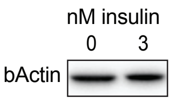

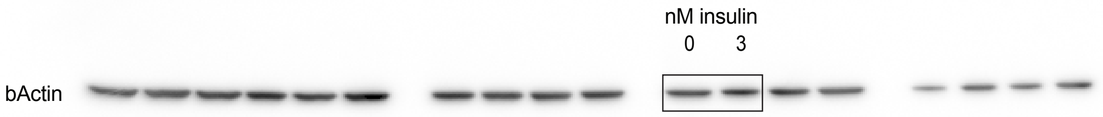

Related to Extended Data Figure 6b

pIRb

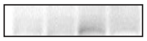

Insulin (nM) 0 0 3 3
Sensitive (S) S R S R
Resistant (R)

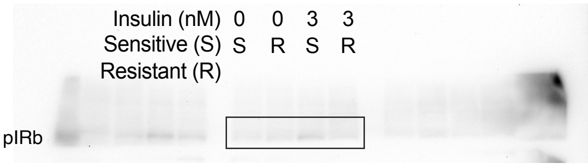

Related to Extended Data Figure 6b

IRb
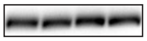
Insulin (nM) 0 0 3 3
Sensitive (S) S R S R
Resistant (R)

Insulin (nM) 0 0 3 3
Sensitive (S) S R S R
Resistant (R)

IRb
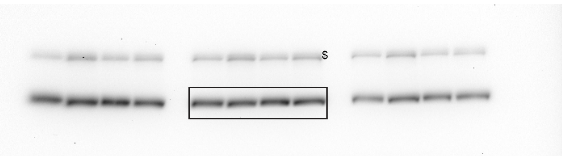
$ IR proform

Related to Extended Data Figure 6c

pIRS1

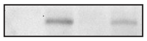

Insulin (nM) 0 3 0 3
S/R S S R R

Insulin (nM) 0 3 0 3
S/R S S R R

pIRS1

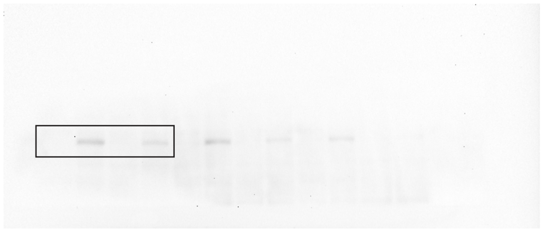

Related to Extended Data Figure 6c

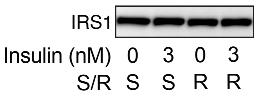

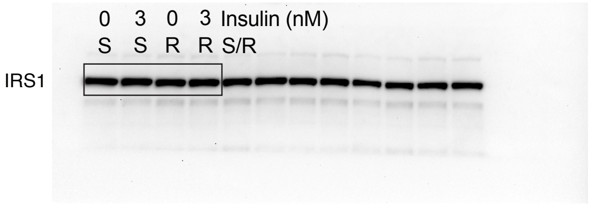

Related to Extended Data Figure 6d

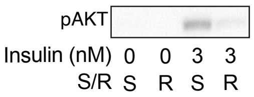

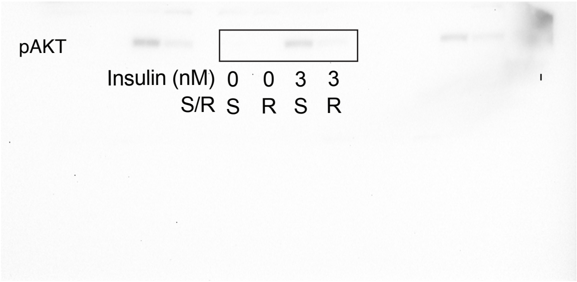

Related to Extended Data Figure 6d

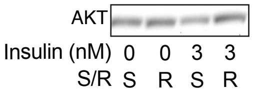

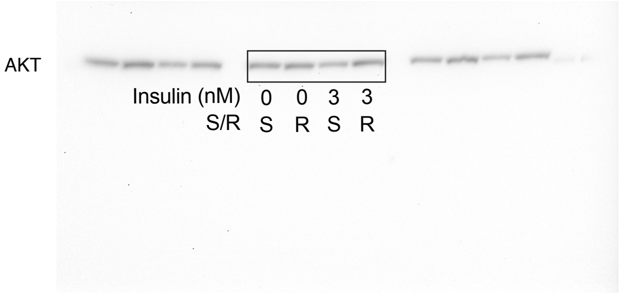

Related to Extended Data Figure 6e

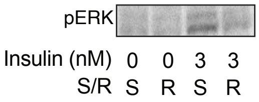

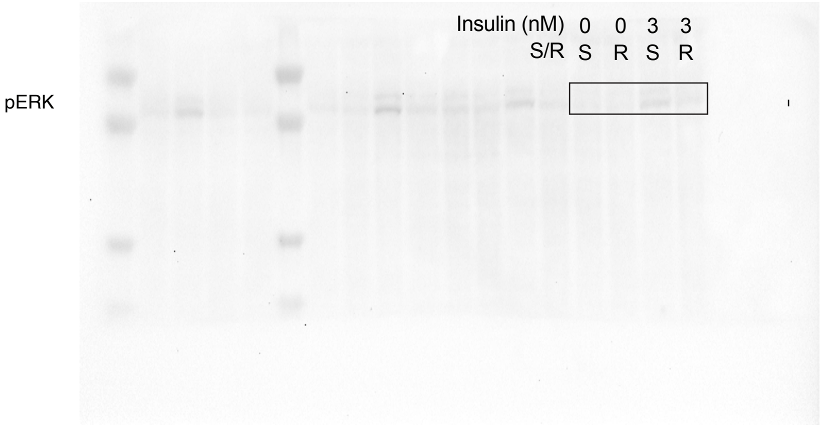

Related to Extended Data Figure 6e

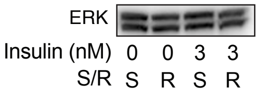

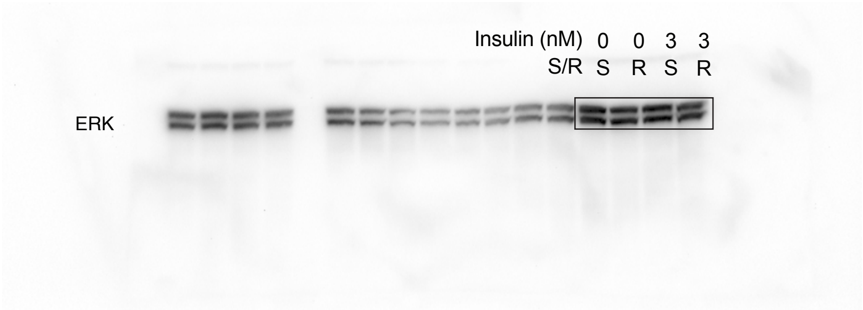

Related to Extended Data Figure 6j

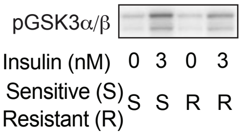

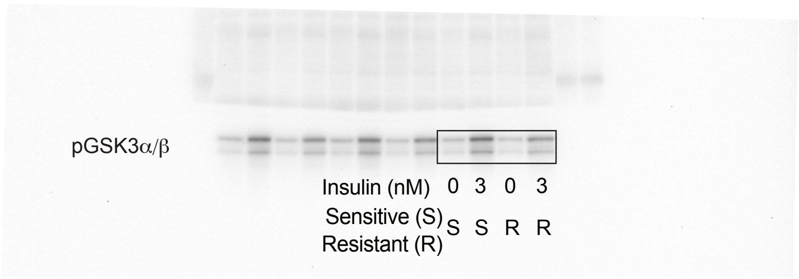

Related to Extended Data Figure 6j

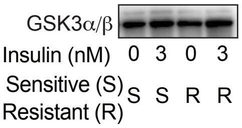

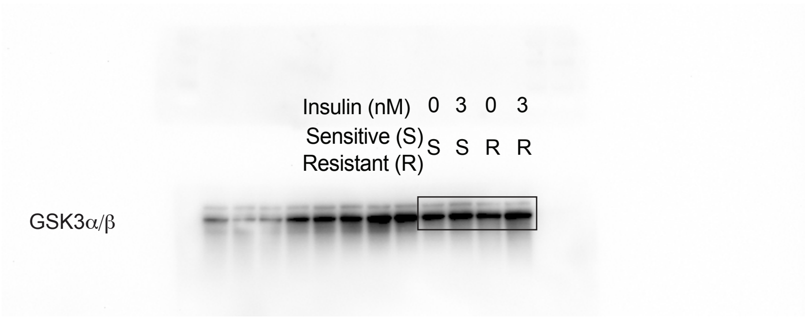

Related to Extended Data Figure 6k

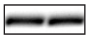

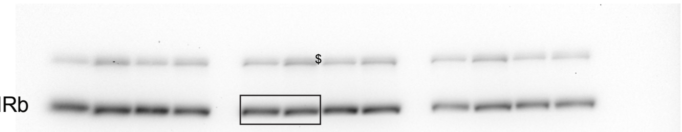

$ IR proform

Related to Extended Data Figure 6k

b-Actin 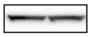
Insulin (nM) 0 0
S/R S R

b-Actin
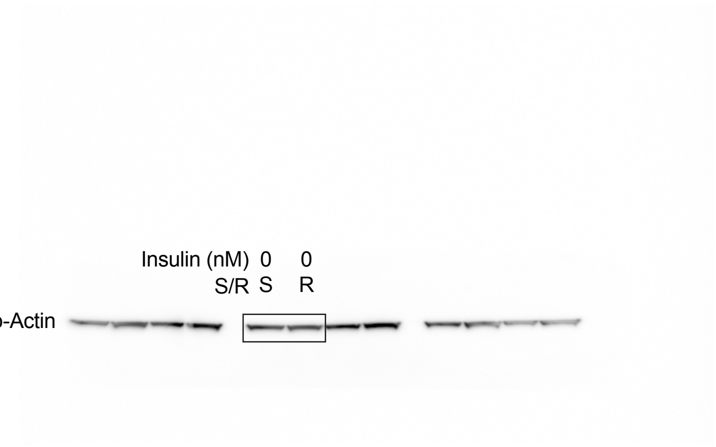

Related to Extended Data Figure 6m

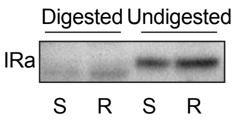

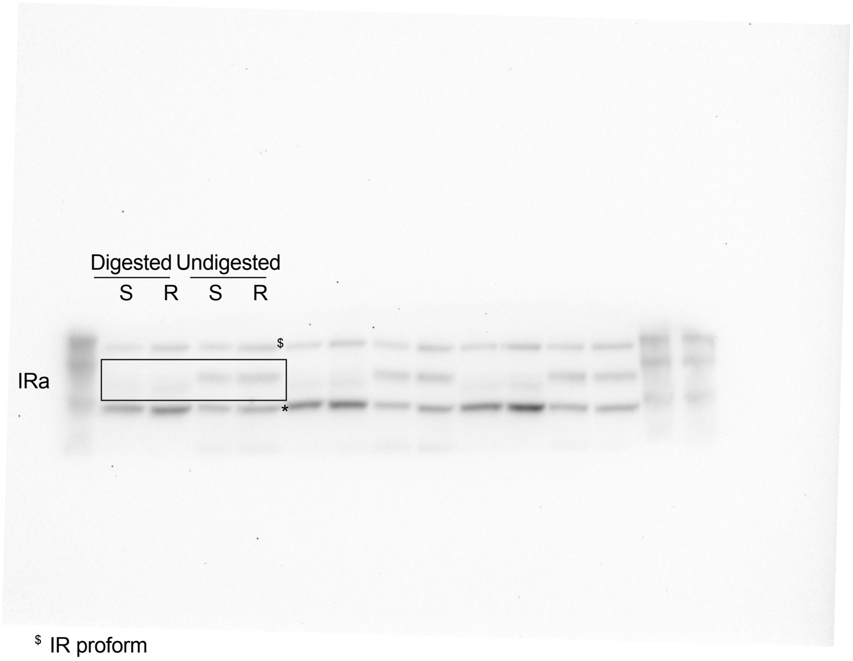

$ IR proform

* Unspecific band

Related to Extended Data Figure 6m

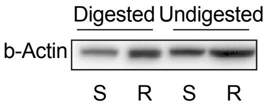

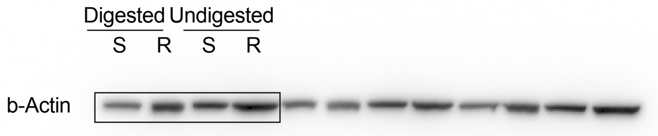

Related to Extended Data Figure 8b

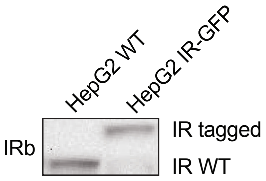

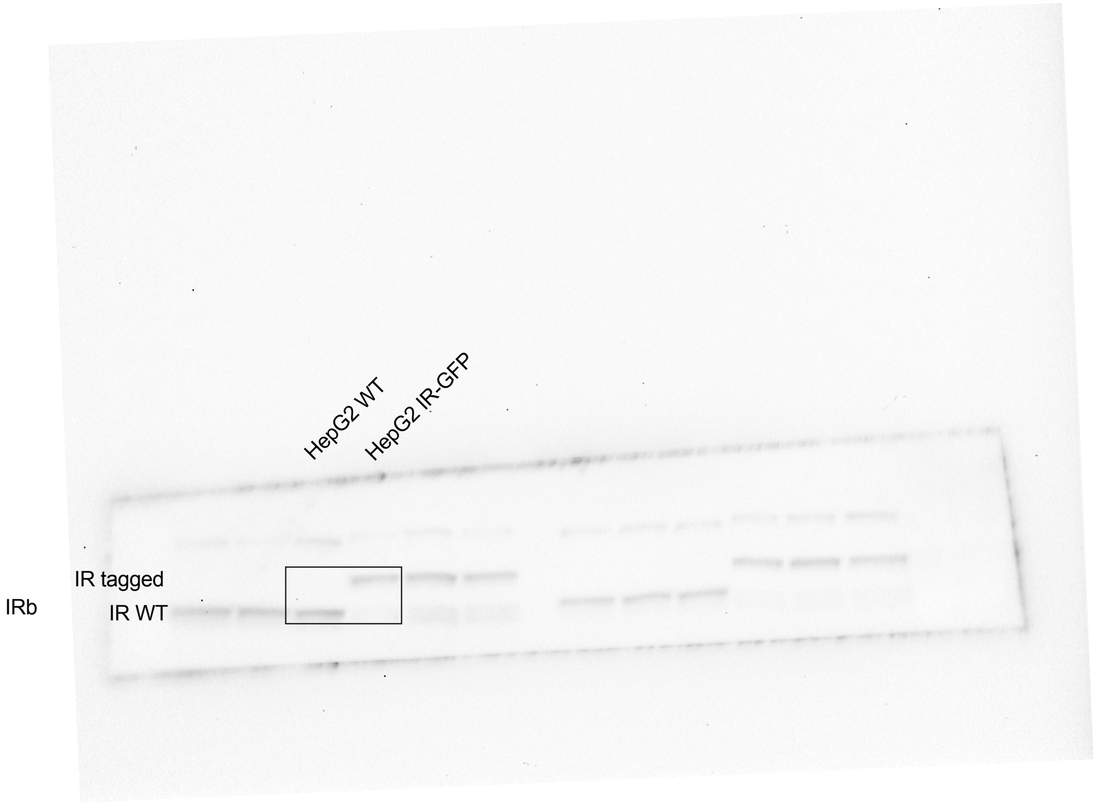

Related to Extended Data Figure 8b

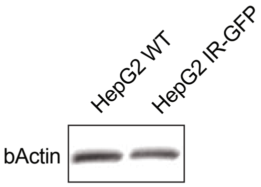

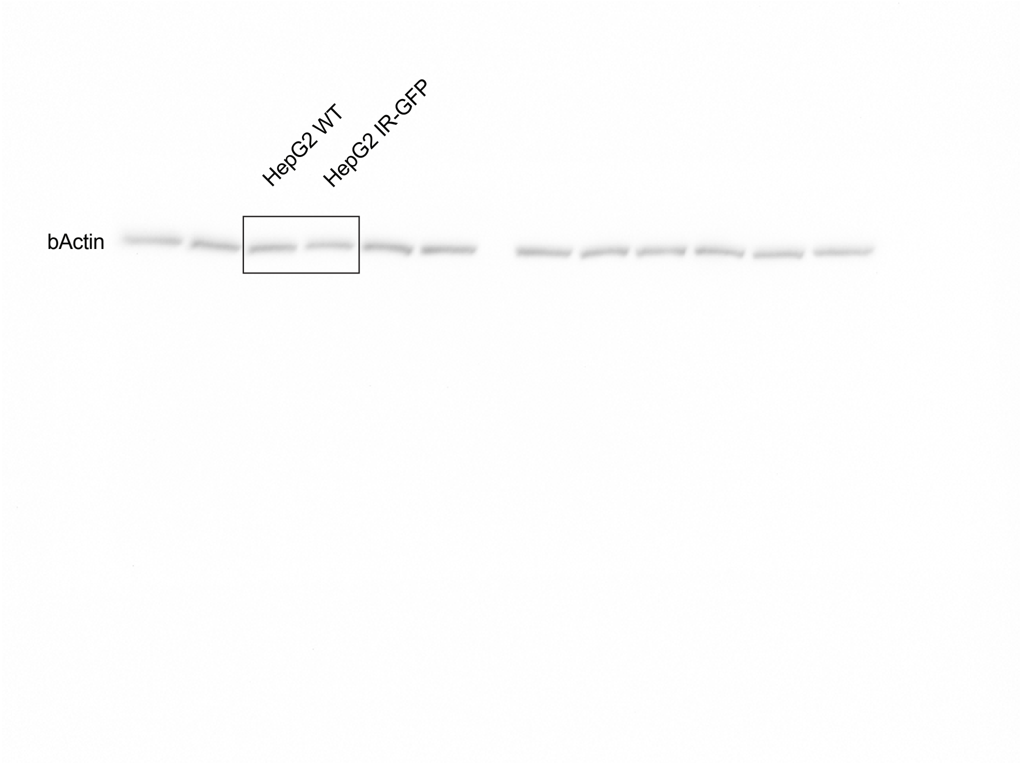

Related to Extended Data Figure 8b

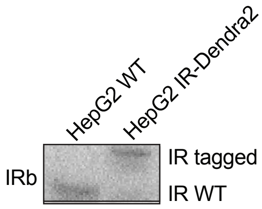

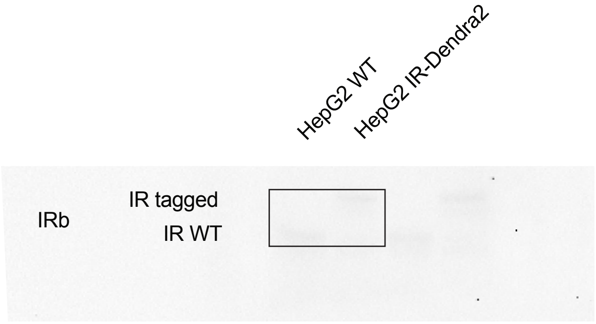

Related to Extended Data Figure 8b

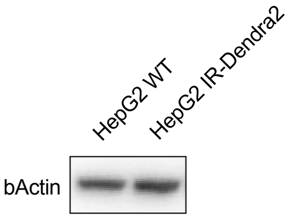

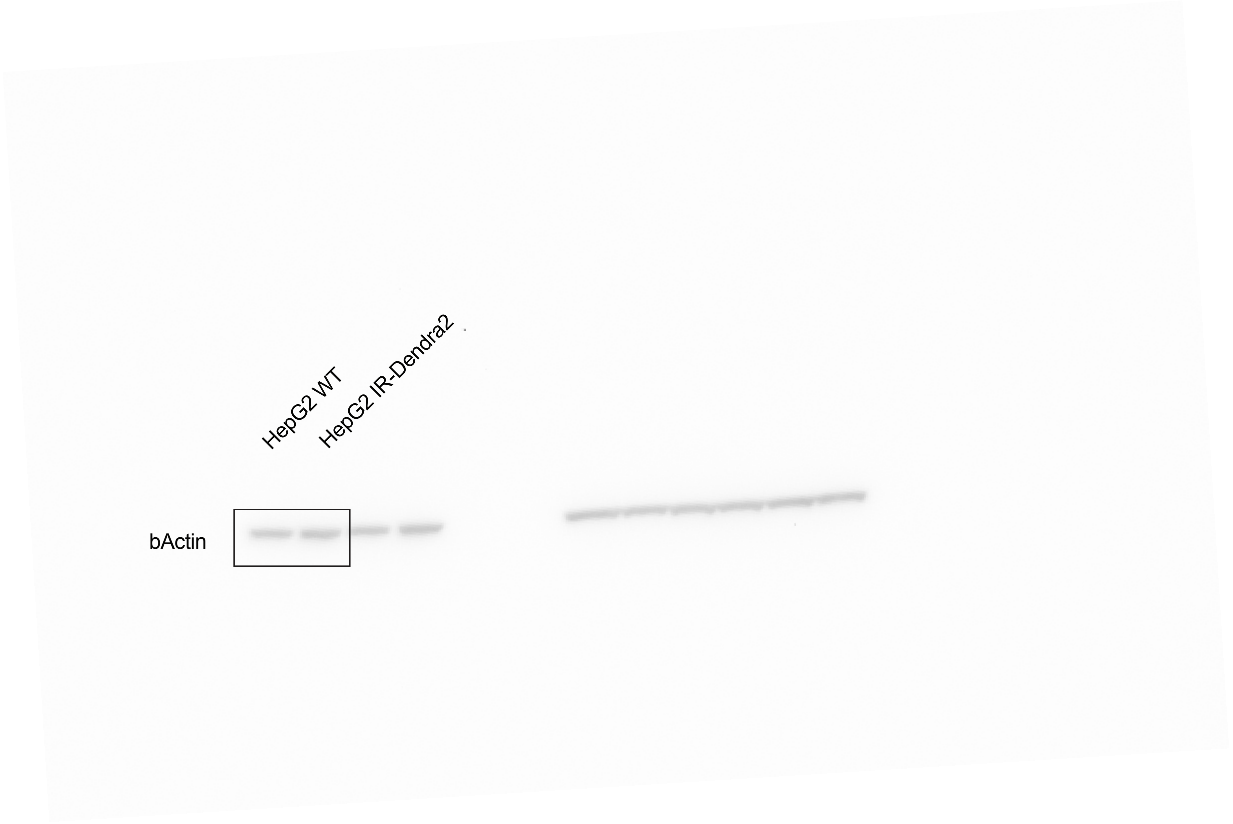

Related to Extended Data Figure 8c

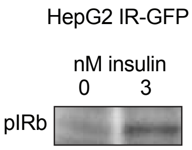

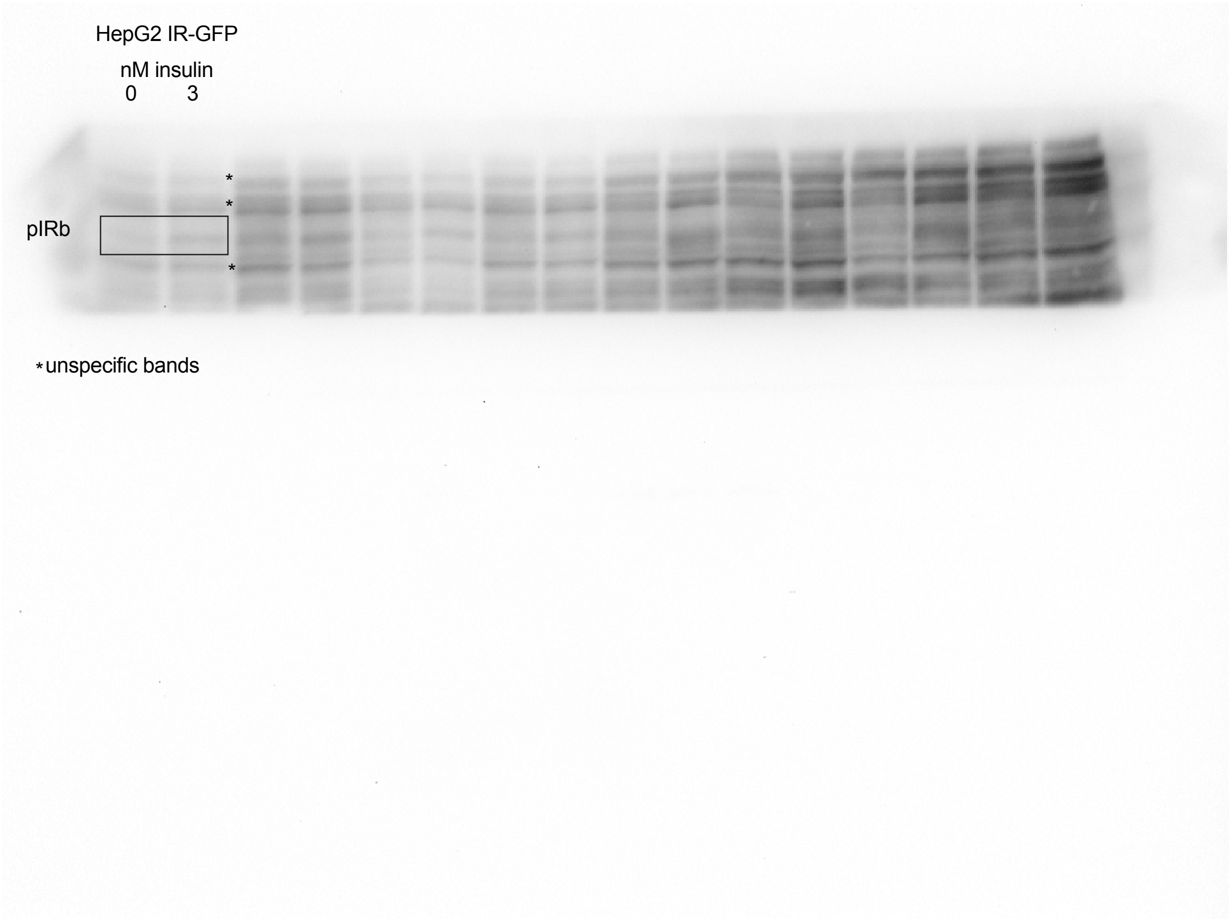

Related to Extended Data Figure 8c

HepG2 IR-GFP

nM insulin
0 3

IRb

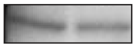

3

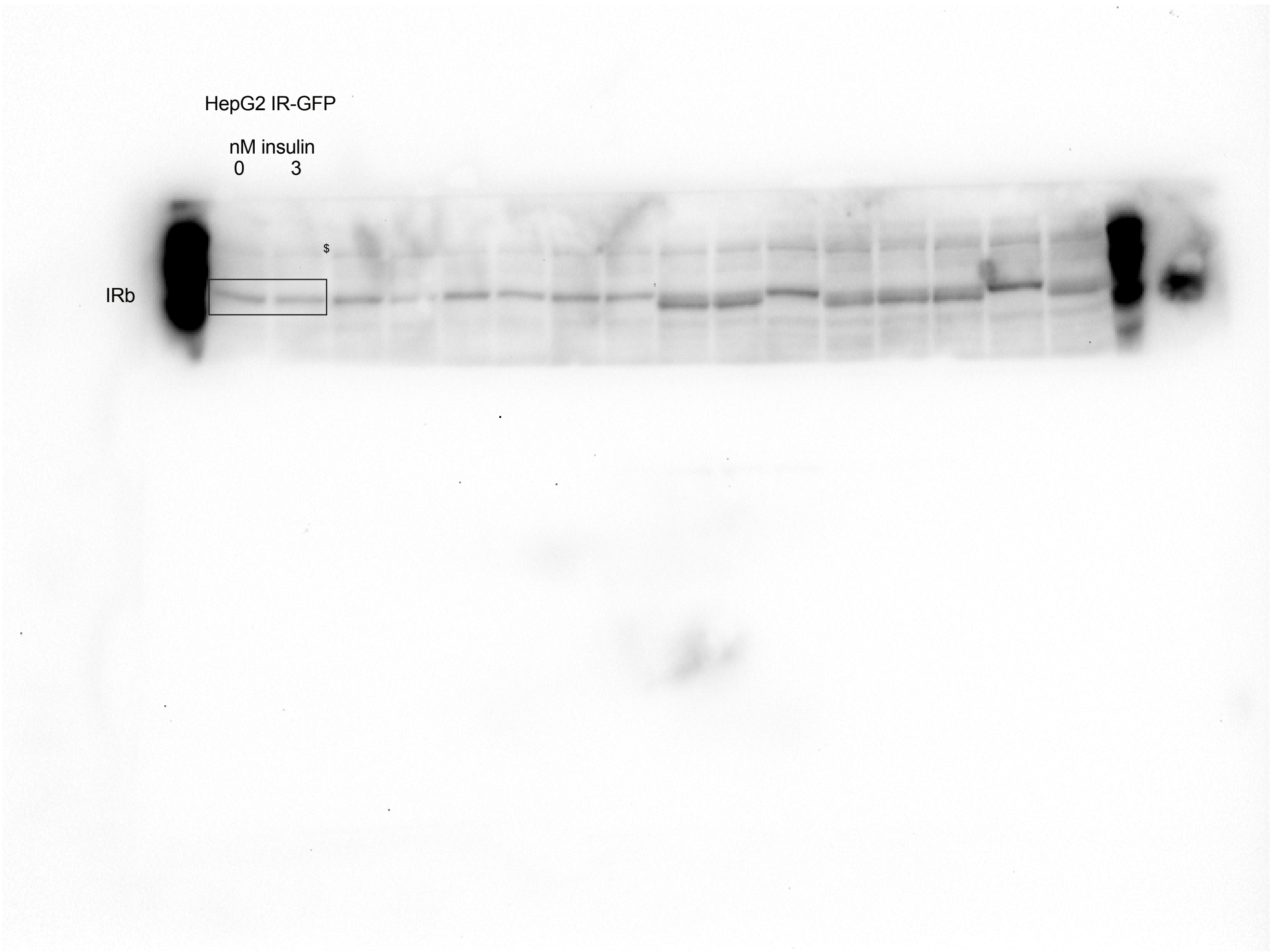

$ IR proform

Related to Extended Data Figure 8c

HepG2 IR-GFP

nM insulin

0 3

AKT pT308

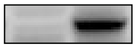

HepG2 IR-GFP

nM insulin

0 3

AKT pT308

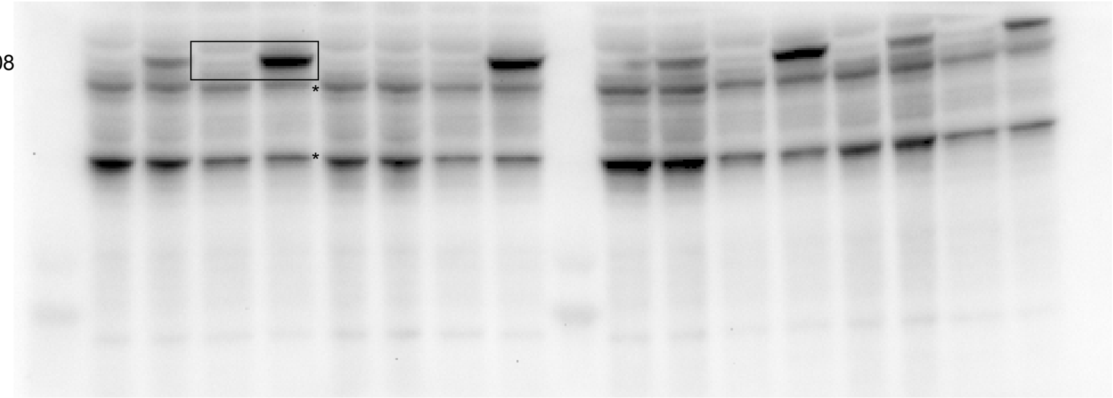

* Unspecific bands

Related to Extended Data Figure 8c

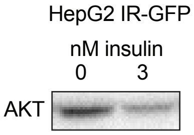

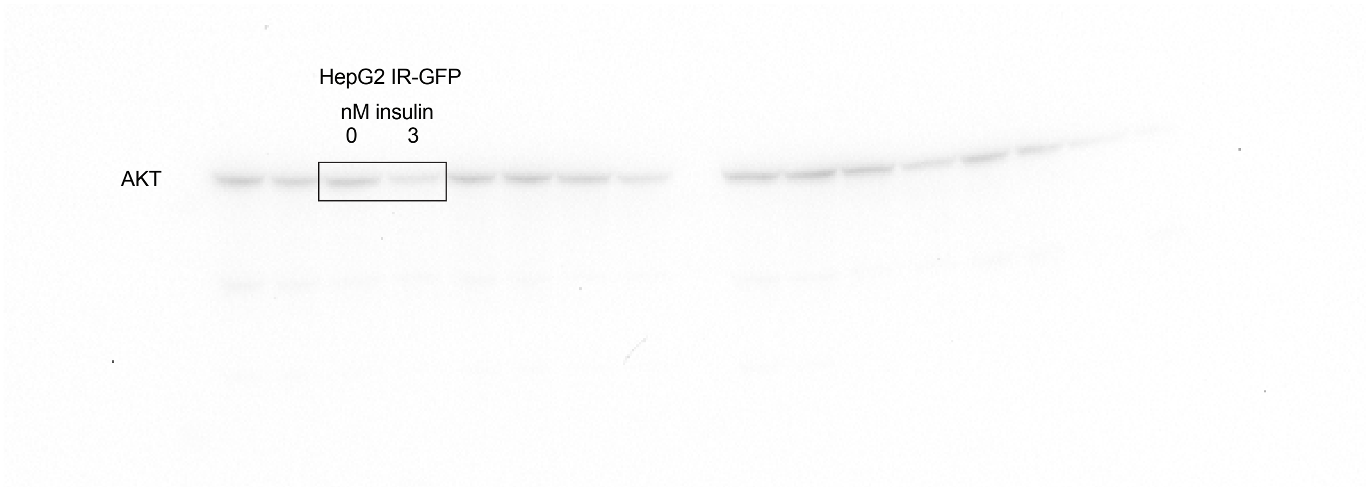

Related to Extended Data Figure 8c

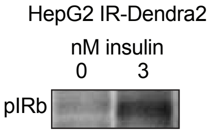

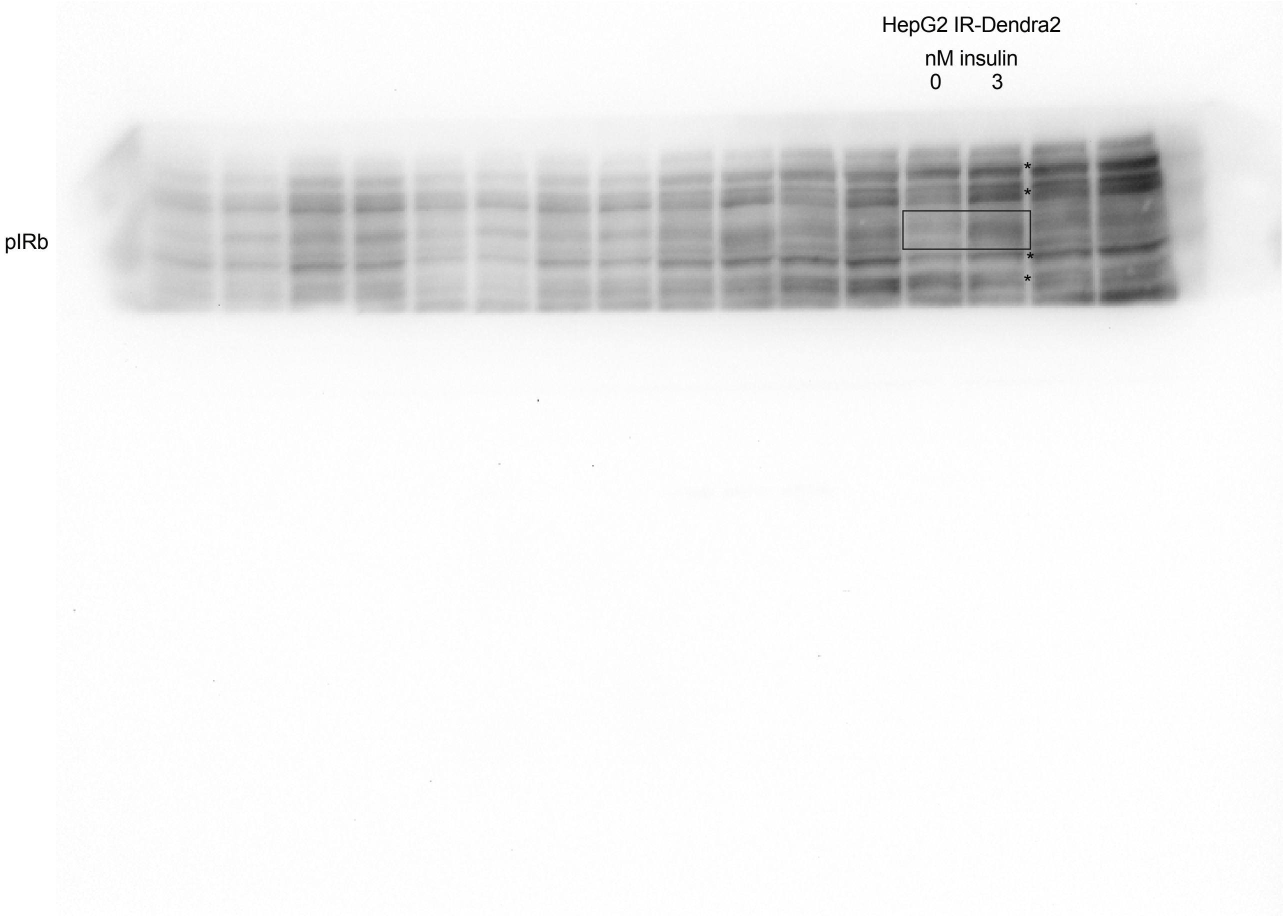

* unspecific bands

Related to Extended Data Figure 8c

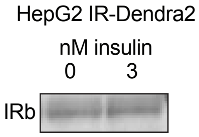

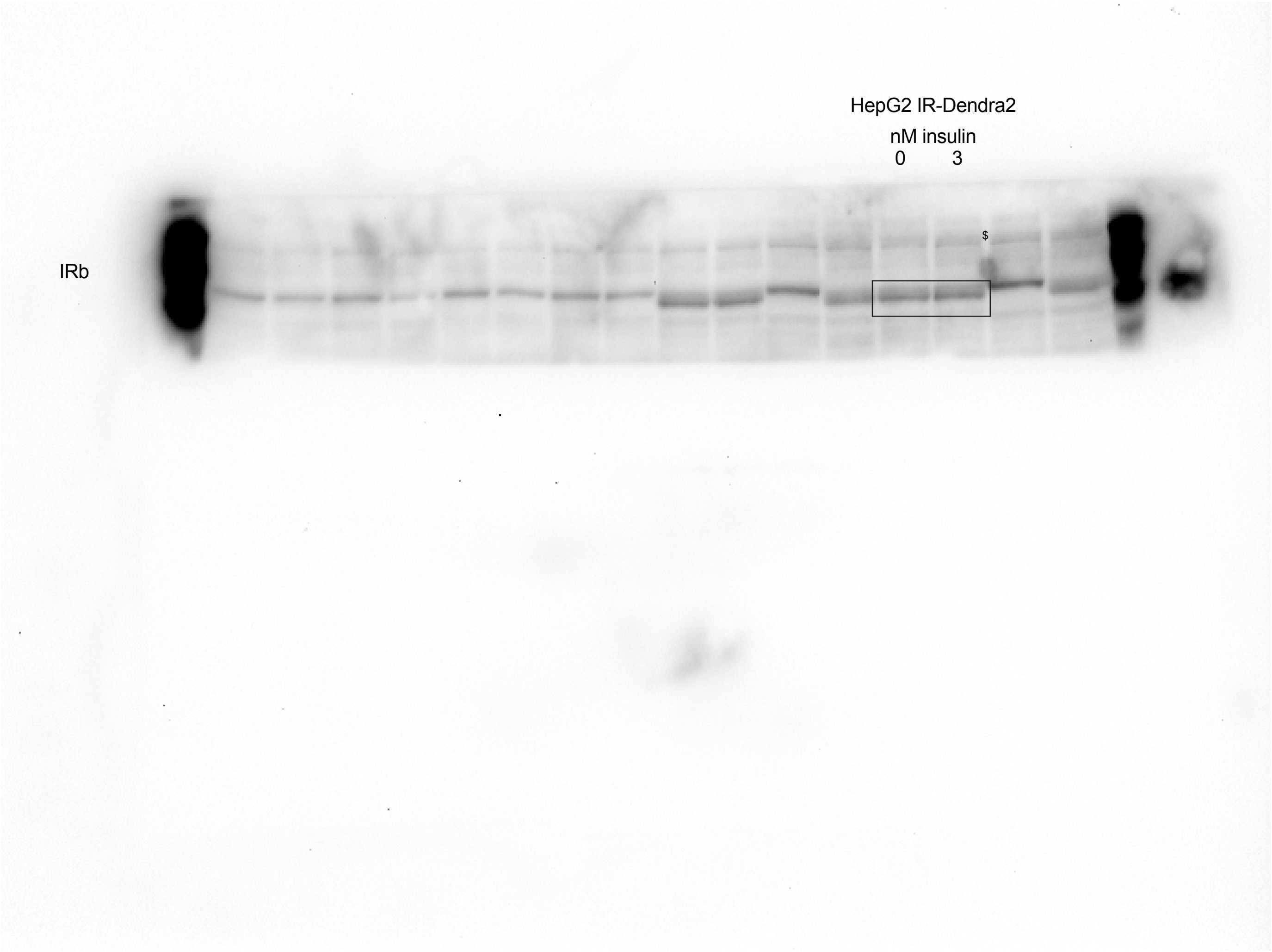

$ IR proform

Related to Extended Data Figure 8c

HepG2 IR-Dendra2

nM insulin
0 3

AKT pT308

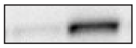

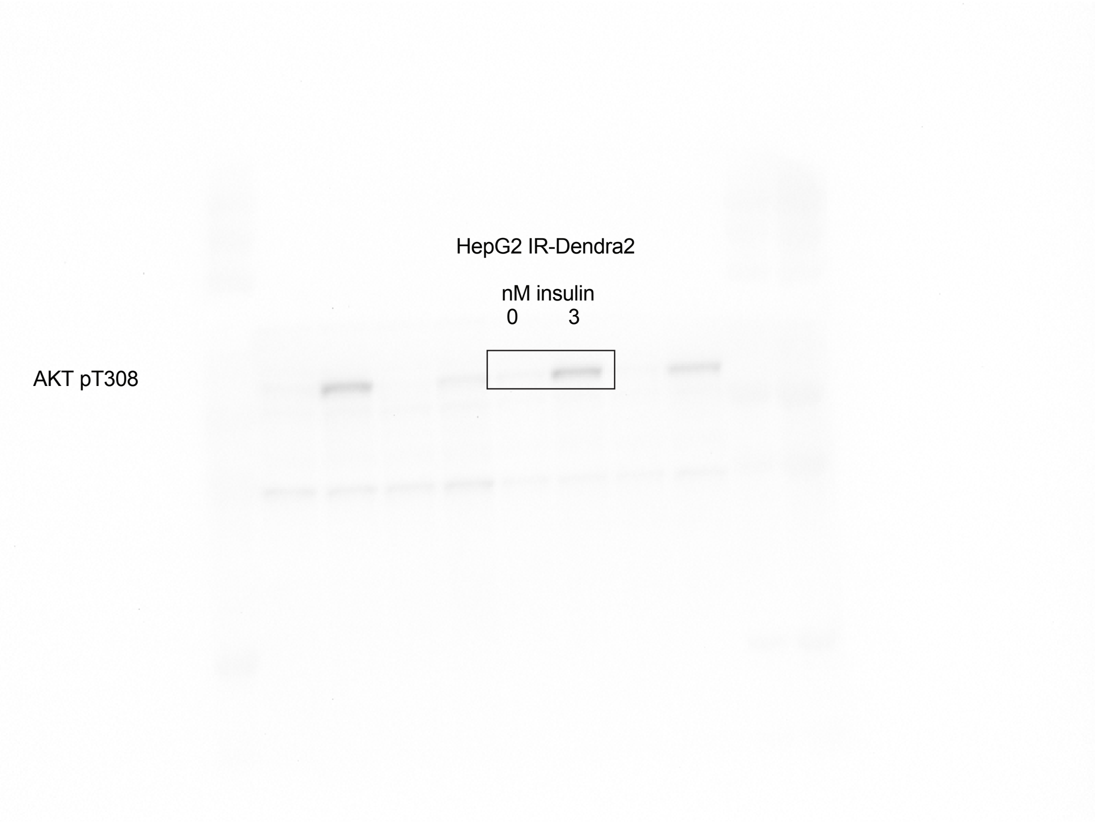

Related to Extended Data Figure 8c

HepG2 IR-Dendra2

nM insulin
0 3

AKT

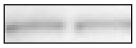

HepG2 IR-Dendra2

nM insulin
0 3

AKT

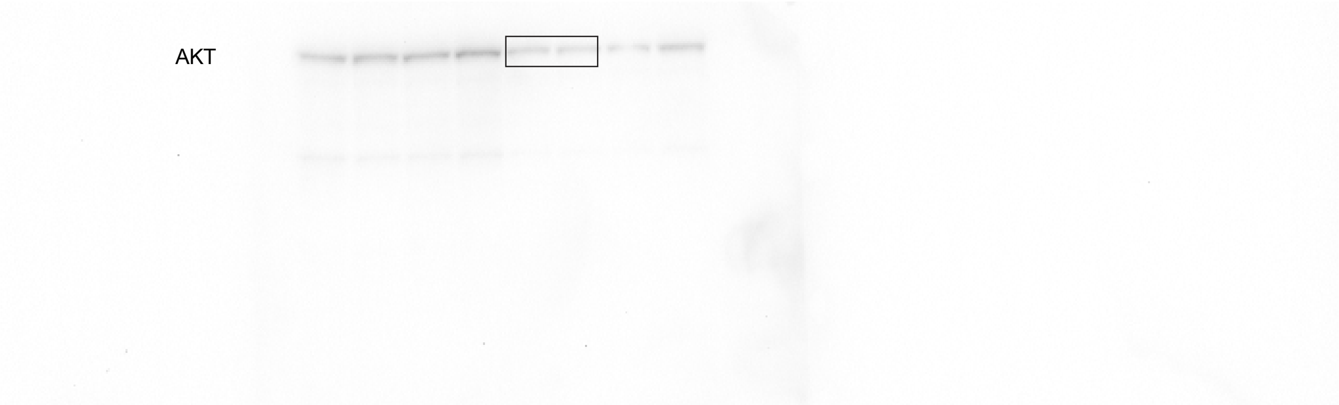

Related to Extended Data Figure 10c

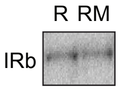

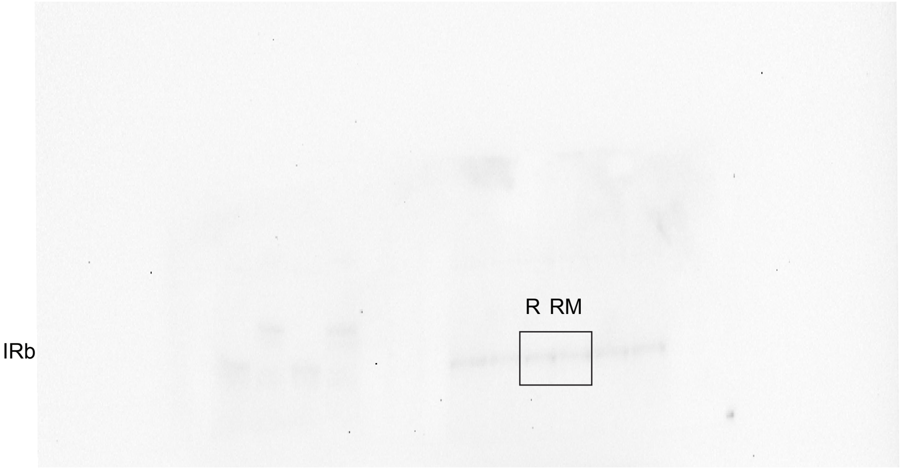

Related to Extended Data Figure 10c

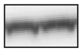

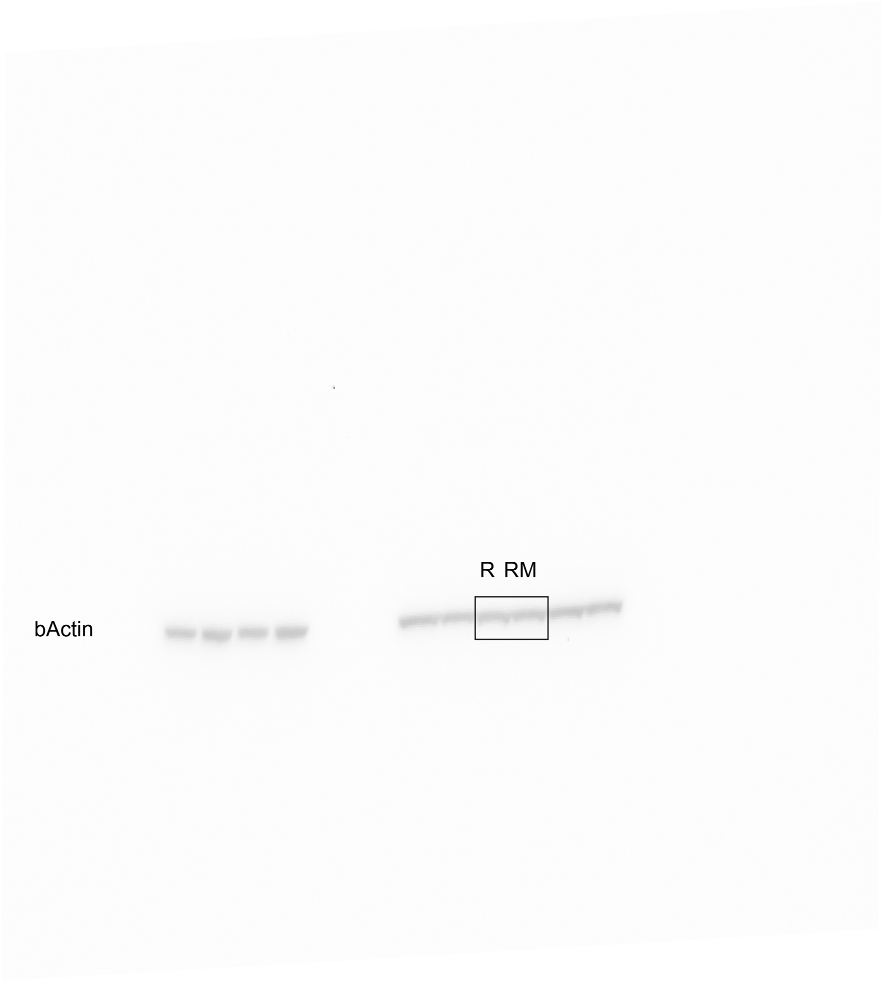

Related to Extended Data Figure 18

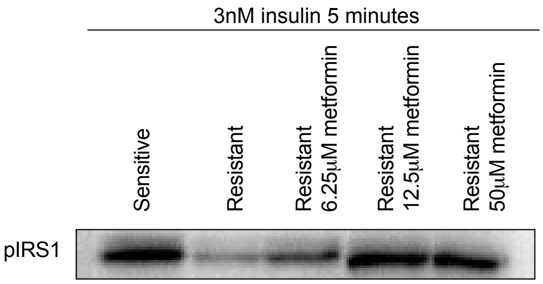

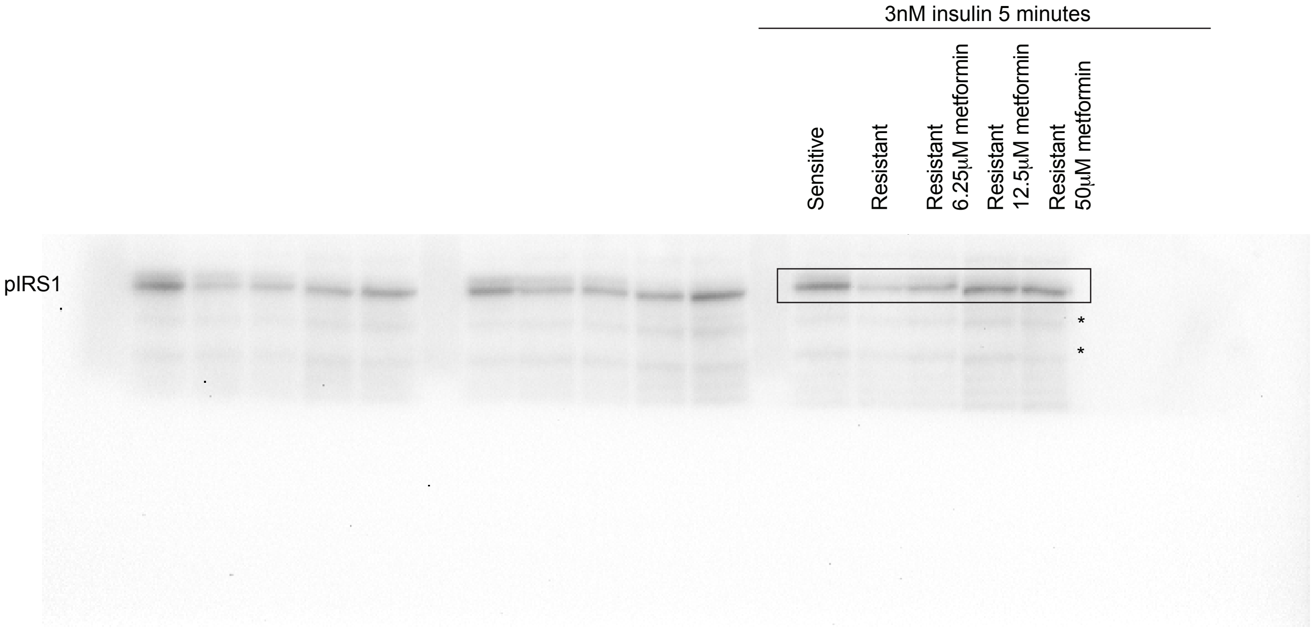

* Unspecific bands

Related to Extended Data Figure 18

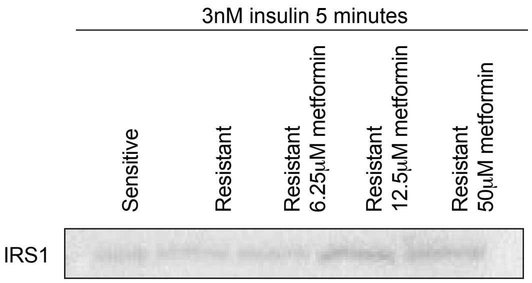

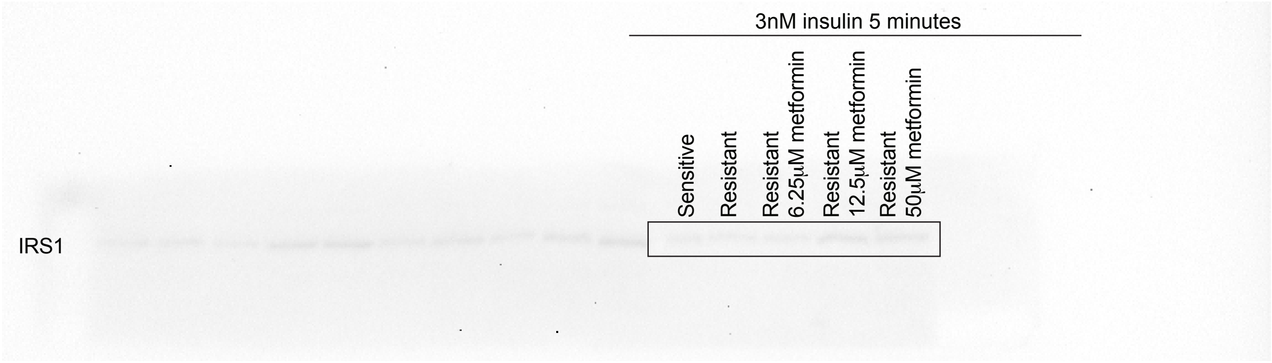

Related to Extended Data Figure 19a

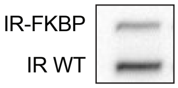

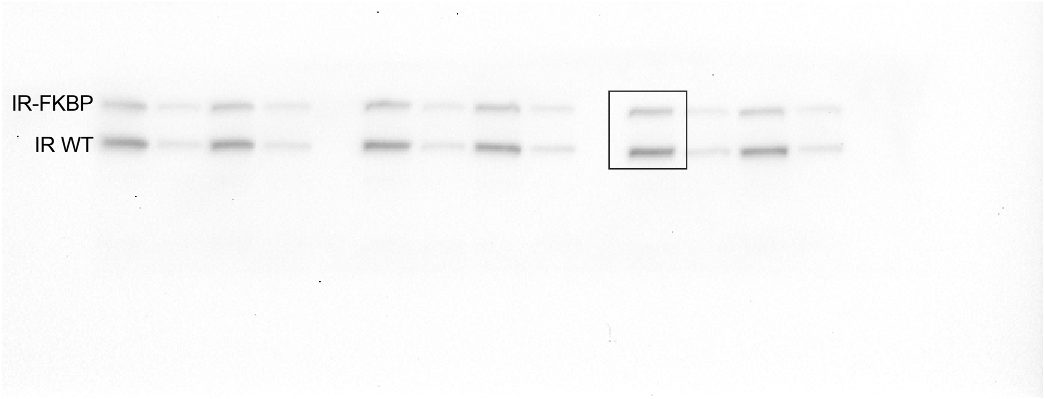

Related to Extended Data Figure 19b

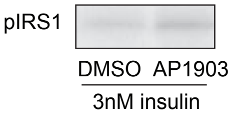

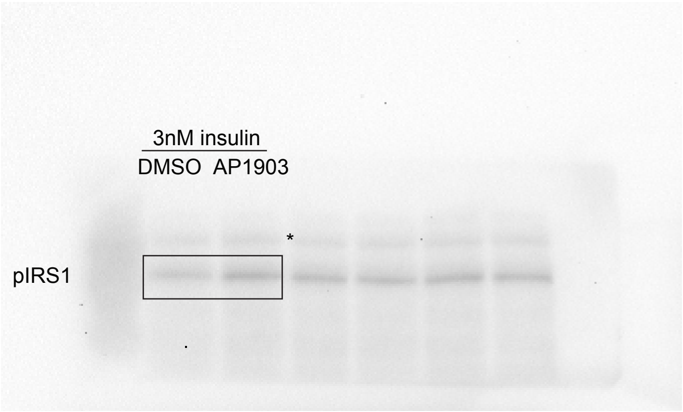

* Unspecific bands

Related to Extended Data Figure 19b

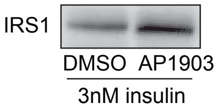

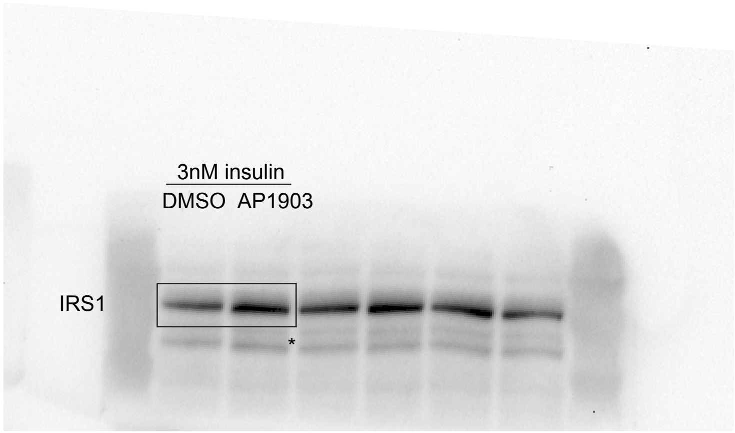

* Unspecific bands
